# Identification and comparative analysis of sixteen fungal peptidyl-prolyl *cis/trans *isomerase repertoires

**DOI:** 10.1186/1471-2164-7-244

**Published:** 2006-09-22

**Authors:** Trevor J Pemberton

**Affiliations:** 1 Institute for Genetic Medicine, Keck School of Medicine, University of Southern California, 2250 Alcazar Street, Los Angeles, CA 90033, USA

## Abstract

**Background:**

The peptidyl-prolyl *cis*/*trans *isomerase (PPIase) class of proteins is present in all known eukaryotes, prokaryotes, and archaea, and it is comprised of three member families that share the ability to catalyze the *cis*/*trans *isomerisation of a prolyl bond. Some fungi have been used as model systems to investigate the role of PPIases within the cell, however how representative these repertoires are of other fungi or humans has not been fully investigated.

**Results:**

PPIase numbers within these fungal repertoires appears associated with genome size and orthology between repertoires was found to be low. Phylogenetic analysis showed the single-domain FKBPs to evolve prior to the multi-domain FKBPs, whereas the multi-domain cyclophilins appear to evolve throughout cyclophilin evolution. A comparison of their known functions has identified, besides a common role within protein folding, multiple roles for the cyclophilins within pre-mRNA splicing and cellular signalling, and within transcription and cell cycle regulation for the parvulins. However, no such commonality was found with the FKBPs. Twelve of the 17 human cyclophilins and both human parvulins, but only one of the 13 human FKBPs, identified orthologues within these fungi. hPar14 orthologues were restricted to the Pezizomycotina fungi, and *R. oryzae *is unique in the known fungi in possessing an hCyp33 orthologue and a TPR-containing FKBP. The repertoires of *Cryptococcus neoformans, Aspergillus fumigatus*, and *Aspergillus nidulans *were found to exhibit the highest orthology to the human repertoire, and *Saccharomyces cerevisiae *one of the lowest.

**Conclusion:**

Given this data, we would hypothesize that: (i) the evolution of the fungal PPIases is driven, at least in part, by the size of the proteome, (ii) evolutionary pressures differ both between the different PPIase families and the different fungi, and (iii) whilst the cyclophilins and parvulins have evolved to perform conserved functions, the FKBPs have evolved to perform more variable roles. Also, the repertoire of *Cryptococcus neoformans *may represent a better model fungal system within which to study the functions of the PPIases as its genome size and genetic tractability are equal to those of *Saccharomyces cerevisiae*, whilst its repertoires exhibits greater orthology to that of humans. However, further experimental investigations are required to confirm this.

## Background

The peptidyl-prolyl *cis*/*trans *isomerase (PPIase) class of proteins is traditionally comprised of three distinct protein families, the cyclophilins (cyclosporin A binding proteins), FKBPs (FK506 binding proteins) and parvulins, that are linked by their shared ability to catalyse the bond preceding a proline residue between its *cis *and *trans *forms. However, the recent identification of a cyclophilin-FKBP hybrid protein (FCBP; FK506- and cyclosporin-binding protein) in the protozoan parasite *Toxoplasma gondii *[1] and the identification of three further members of this novel family in two distinct bacteria, *Flavobacterium johnsonii *&*Treponema denticola*, as well as in at least ten other bacteria in the sequence databases (TJP; unpublished data), may indicate a shared early evolutionary history for the cyclophilin and FKBP families. All families, with the exception of the FCBPs that appear to be confined to the bacterial and protist lineages (TJP; unpublished data), are found widely distributed in eukaryotes, prokaryotes and archaea [2-8], implying that their function is required in cellular processes from bacteria to man, and in all the major compartments of the cell [8-11].

Despite their shared conservation throughout nature, the three traditional PPIase families do not share a conserved role in cell viability. In bacteria, the four known periplasmic PPIases in *Escherichia coli *(FkpA, PpiA, PpiD, and SurA) have been reported not to be essential for growth under laboratory conditions [12]. However, two cytosolic PPIases, PpiB and trigger factor, in *Bacillus subtilis *have been shown not to possess an essential function under normal growth conditions, but they become essential for cell viability under starvation conditions [13]. All of the cyclophilins and FKBPs in the budding yeast *Saccharomyces cerevisiae *have been individually and collectively knocked out with no effect on cell viability [14,15]. Only Ess1, the *S. cerevisiae *orthologue of the human parvulin Pin1, has been reported to be essential within *S. cerevisiae *[16]. However, only a very low level is required for cell growth under normal conditions, but a higher level is required in the presence of environmental challenges [17]. This essential cellular role is shared with its orthologue in the pathogenic yeast *Candida albicans *[18], however not with their orthologues in their fellow fungi *Schizosaccharomyces pombe *[19] and *Cryptococcus neoformans *[20], or the fruit fly *Drosophila melanogaster *[21]. It therefore appears likely that the essential function of some Pin1 orthologues is limited to certain organisms or that there is a degree of redundancy present in these other organisms that compensates for its absence.

Recently, a mutation in the *D. melanogaster *cyclophilin CG3511 that severely truncates the protein has been reported to confer a synthetic lethal phenotype on cells that lack the retinoblastoma (Rbf) protein [22]. Mice lacking Pin1 [23], Cyclophilin A [24], FKBP12 [25], FKBP12.6 [26], and FKBP52 [27] have all been found to be viable and to develop normally, although the latter did result in partial embryonic lethality [27]. However, Pin1-deficient mice were found to be at a higher risk of developing Alzheimer's disease [28], and also to have cell-proliferative abnormalities that included decreased body weight, testicular and retinal atrophies, and the failure of the breast epithelial compartment to undergo the normal changes associated with pregnancy [23,29]. FKBP12-deficient mice were found to suffer from severe dilated cardiomyopathy and noncompaction of left ventricular myocardium, which mimics a human congenital heart disorder [25]. Cardiac hypertrophy was found in FKBP12.6-deficient male mice, but not in females, unless the protective effect of oestrogen was abrogated [26]. Finally, FKBP52-deficient male mice were found to have several defects in reproductive tissues which included ambiguous external genitalia and a dysgenic prostate [27].

It therefore appears that despite the high conservation of the PPIases throughout the eukaryotes and prokaryotes, they do not possess an essential function within many cells under normal growth conditions, but may become essential in the absence of other cellular factors or in response to environmental challenges. Also, whilst PPIases appear not to be essential for the viability of the mouse, their haploinsufficiency may result in abnormalities that impact the fitness of the animal and which could be models for human disease.

Whilst some fungi have found commercial applications, such as *S. cerevisiae*, *Sz. pombe *and *R. oryzae *in fermenting, others have been identified as both human [30-35] and plant [36-38] pathogens. PPIases within some bacterial pathogens have been found to have a key role in their pathogenicity [39-45], and a cyclophilin within the pathogenic fungus *Cryptococcus neoformans *has been reported as important for its virulence [46]. However, in most cases it is unknown what role, if any, fungal PPIases play in the pathogenicity of their host cell. Until we identify and understand the PPIase repertoires of these pathogens, we cannot begin to unravel their potential roles within the cell and their use as putative therapeutic targets.

Reported here is the identification and comparative analysis of the PPIase repertoires present in sixteen fungi that represent four different fungal taxa; Ascomycota (*Candida albicans*, *Candida glabrata*, *Debaryomyces hansenii*, *Eremothecium gossypii*, *Kluyveromyces lactis*, *Saccharomyces cerevisiae*, *Schizosaccharomyces pombe*, *Yarrowia lipolytica*, *Aspergillus fumigatus*, *Aspergillus nidulans*, *Gibberella zeae*, *Neurospora crassa*), Basidiomycota (*Cryptococcus neoformans*, *Ustilago maydis*), Microsporidia (*Encephalitozoon cuniculi*) & Zygomycota (*Rhizopus oryzae*). By comparing these fungal repertoires, we hope to identify key conserved PPIases that are found within all their repertoires as well as those that are specific to each fungal taxa or fungus. By compiling the known functions of these PPIases, we hope to better understand both those that function within a broad range of fungi as well as those that are specific to a particular fungal linage that may be linked to their specific characteristics. This comparison will also serve to aid our interpreting of the use of fungal model systems in furthering our understanding of the roles of PPIases within vastly different cell types.

## Results

### Identification of PPIase repertoires

The PPIase repertoires of the different fungi were identified by BLASTP and TBLASTN searches of their proteome and genome, respectively, maintained by the National Center for Biotechnology Information (NCBI) using the protein sequences of human cyclophilin A, FKBP12, and Pin1 as probes to identify the cyclophilin, FKBP, and parvulin families, respectively. The cyclophilins were all identified with an E-value between 10^-10^-10^-69 ^and a sequence identity between 28–73% with the exception of six proteins. These six proteins are members of two previously identified cyclophilin groups that possess a divergent PPIase domain [11] and they gave E-values <10^-10 ^and a sequence identify <30% when compared against hCypA. These cyclophilins were identified using the *Sz. pombe *and *S. cerevisiae *members of these groups [See Table 16 in Additional File 1]; Group K – DhCyp6, AfCyp8, & UmCyp6; Group L – YlCwc27, DhCwc27, & EgCwc27). The Group K members gave E-values between 10^-56^-10^-109 ^and a sequence identify between 40–60%, and the Group L members gave E-values between 10^-20^-10^-37 ^and a sequence identity between 27–31%. The FKBPs were all identified with E-values between 10^-13^-10^-38 ^and a sequence identity between 33–62%. The parvulins were all identified with E-values between 10^-19^-10^-49 ^and a sequence identity between 34–55% with the exception of four that gave E-values between 10^-6^-10^-8 ^but retained a sequence identity between 33–37% (AfPar1, AnPar1, GzPar1, & NcPar1). Upon inspection, these proteins possessed a clearly identifiable parvulin-like rotamase domain but lacked the characteristic WW domain of the metazoan Pin1 proteins. Searching of the fungal sequence databases using the human parvulin Par14 as a probe identified these four parvulins with E-values between 10^-28^-10^-32 ^and a sequence identity between 54–59%, confirming them as parvulins.

**Figure 1:**
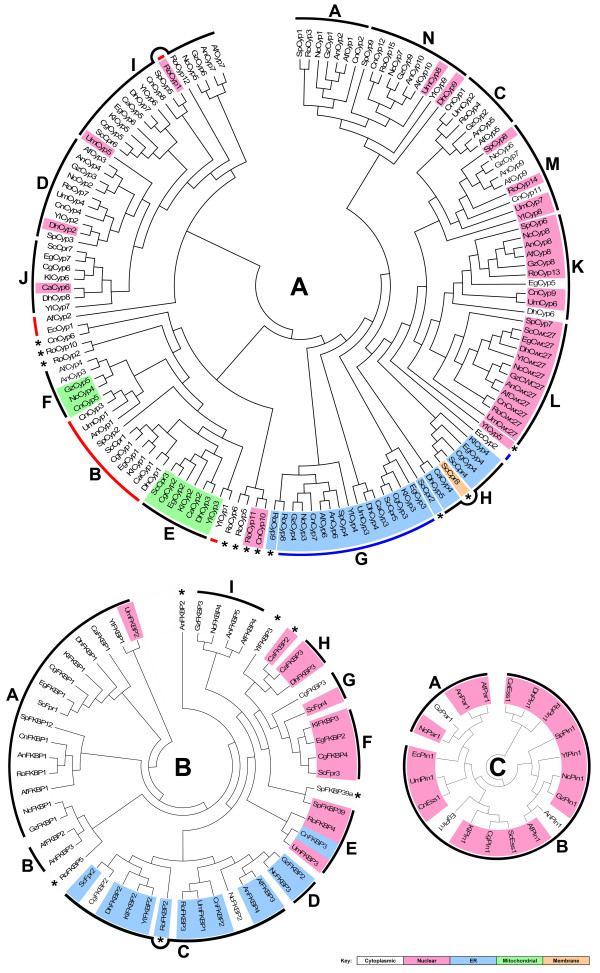
**Radial dendrograms depicting the predicted evolutionary history of the three fungal PPIase families: **(**A**) the cyclophilins, (**B**) the FKBPs and (**C**) the parvulins of *A. nidulans *(An), *A. fumigatus *(Af), *C. albicans *(Ca), *C. glabrata *(Cg), *C. neoformans *(Cn), *D. hansenii *(Dh), *E. cuniculi *(Ec), *E. gossypii *(Eg), *G. zeae *(Gz), *K. lactis *(Kl), *N. crassa *(Nc), *R. oryzae *(Ro), *S. cerevisiae *(Sc), *Sz. pombe *(Sp)*, U. maydis *(Um), &*Y. lipolyica *(Yl) based upon a comparison of their protein sequences by the ClustalX program version 1.83 [157] with the dendrogram visualized using MEGA version 3.1 [159]. Black bars indicate the locations of the groups identified in Table 16 [See Additional File 1] that cluster together at one location in the dendrogram and they are labelled with the appropriate group letter. In (**A**), red bars give the locations of the cyclophilin Group B members and blue bars give the locations of cyclophilin Group G members. The scales of the different dendrograms are not cross-comparable.

The identified PPIase repertoires within the fungal genomes investigated can be found in Tables 1, 2, 3, 4, 5, 6, 7, 8, 9, 10, 11, 12, 13, 14. Table 15 shows a comparison of the number of members of each PPIase family found within the different fungi. The repertoire orthology of the cyclophilins, FKBPs and parvulins as identified by BLAST analysis can be found in Table 16 [See Additional File 1]. Figure 1 shows the dendrograms generated for the cyclophilins (A), FKBPs (B), and parvulins (C). Pairwise E-values, bit scores, and percentage sequence similarity and identity between all members of each cyclophilin, FKBP, and parvulin group, as well as a global comparison for each family, can be found in Additional Files 2 &3 (cyclophilins), 4 &5 (FKBPs) and 6 &7 (parvulins). The multiple sequence alignments for each cyclophilin, FKBP, and parvulin group discussed herein can be found in Additional File 8.

### Comparison of PPIase repertoire sizes

The number of PPIases present within these fungi (Table 15) varies from three in *E. cuniculi *to 22 in *R. oryzae*. To compare the sizes of the different repertoires, the number of PPIases has been weighted by the number of genes in their genome. The average number of PPIases was 1.9 per 1000 genes in the genome (Table 15), with the most PPIase rich fungi being *Sz. pombe*, which has just over 2.5 per 1000 genes in its genome, and the most PPIase poor fungi being *G. zeae*, which has just over 1 per 1000 genes in its genome. These differences are also reflected in the taxa, with the Pezizomycotina fungi having an average number of PPIases of 1.54 per 1000 genes, compared to the Saccharomycotina fungi which have 2.02 per 1000 genes. Looking at the weighted number of each PPIase family in these two taxa, this difference appears due to a reduced number of cyclophilins and FKBPs within the Pezizomycotina fungi. It also appears that *Sz. pombe *is PPIase rich due to an above average number of cyclophilins and FKBPs, whilst *G. zeae *is PPIase poor due to a below average number of all three PPIase families.

Cyclophilin numbers in the different fungi vary predominantly between 6 and 11, however at the extremes *E. cuniculi *has only 2, and *R. oryzae *has 16 (Table 15). The most cyclophilin rich fungi is *C. neoformans *with 1.98 per 1000 genes, closely followed by *Sz. Pombe *with 1.87 per 1000 genes, and the most cyclophilin poor fungi is *G. zeae *with 0.71 per 1000 genes, followed by *N. crassa *with 0.89 per 1000 genes. The number of FKBPs in these fungi is typically 3 or 4, with the extremes being none in *E. cuniculi *and 5 in *R. oryzae*, which is in keeping with their respective genome sizes (Table 15). The most FKBP rich fungi is *C. glabrata *with 0.76 per 1000 genes, closely followed by *S. cerevisiae *with 0.68 per 1000 genes and *Sz. pombe *with 0.62 per 1000 genes, and the most FKBP poor fungi is *E. cuniculi *with none, followed by *G. zeae *with 0.21 per 1000 genes and *R. oryzae *with 0.29 per 1000 genes, with the latter being surprising given that it has the largest PPIase repertoire of these fungi (Table 15). There is on average just a single parvulin in these fungi (Table 15), with the exception being the presence of a second in members of the Pezizomycotina taxa, but this number is in keeping with their genome size. The most parvulin poor fungus is *R. oryzae*, which has just the sole parvulin despite it having a genome size larger than that of the Pezizomycotina fungi. The most parvulin rich is *E. cuniculi*, with its sole parvulin but very small genome size compared with the other fungi.

Overall, *G. zeae *and *R. oryzae *have the sparsest PPIase repertoires of these fungi, with both lacking the number of members in all three PPIase families that would be predicted based on their genome sizes. *Sz. pombe *and *C. neoformans *have the densest PPIase repertoires of these fungi, with the former having an increased number of cyclophilins and FKBPs over the expected value, whilst the latter solely has a larger than expected cyclophilin repertoire.

### Cyclophilin orthology

Table 16A [See Additional File 1] shows that there are only two cyclophilin groups that are conserved throughout all the fungi. One group are the human cyclophilin A orthologues (Group B), a ubiquitous group of cyclophilins that have been reported to be cytoplasmic, in agreement with the PSORT predicted localization for all members except RoCyp1 (Table 16A [See Additional File 1]), with an appreciable nuclear component [10,47-49], which given the absence of nuclear localisation sequences (NLS) within their sequences is likely due to interactions with target proteins that shuttle them into the nuclear compartment. However, SpCyp2 has been reported to be present in discrete vesicles within the cytoplasm [11], and RoCyp1 was predicted by PSORT to be nuclear, which is in partial agreement with the observed localization for members of this group, implying that the localization of this group may be variable between different organisms. This is supported by the observation that the cyclophilin A orthologue in *N. crassa *has two isoforms that localize to the cytoplasm or the mitochondrion, dependant upon the cleavage of a N-terminal signal peptide [50]. Similar dual functions may also exist with other members of this group, but they have not yet been identified. Members of this group have been reported to function in protein activity regulation [51], transcriptional regulation [51-53], a vesicular import pathway [54], the control of both the meiotic [46,49] and mitotic [46,55] cell cycles, and in the mediation of the virulence of *C. neoformans *[46], indicating that they have wide ranging functions within the cell.

The second group that is present in all the fungi is that of the human cyclophilin B orthologues (Table 16A [See Additional File 1]; Group G). This group is identified by their targeting to the endoplasmic reticulum (ER) by an N-terminal signal peptide [9-11,56,57], in agreement with their PSORT predicted localization (Table 16A [See Additional File 1]). AnCyp6 has been reported to be upregulated during heat shock and it is capable of inhibiting calcineurin in the presence of CsA, but it is not essential for cell growth [57]. A second cyclophilin in both *R. oryzae *(RoCyp9) and *S. cerevisiae *(ScCpr2) appears to be a paralogue of their respective Group B member, suggesting that these fungi may be evolving divergent functions within their ER that require a second ER cyclophilin related to Group G. ScCpr2 has been reported to be present in the fungi's secretory pathway [10,14,58] and induced by heat stress and tunicamycin [59], confirming its localization. Their presence in the ER and up-regulation during heat shock would strongly suggest a role within the vesicular protein folding pathway, presumably as a folding catalyst and chaperone.

Only one group is present in all but one of the fungi, *E. cuniculi*. The human cyclophilin 40 orthologues (Table 16A [See Additional File 1]; Group I) are a group of heat shock inducible [11,60-62] predominantly nuclear [10,11] cyclophilins. They are predicted by PSORT to be cytoplasmic and possess no NLS with the exception of UmCyp5, suggesting that their presence in the nucleus is through interactions with target factors that shuttle them into the nucleus rather than their direct targeting. Members of this group have been reported to function within the Hsp90 complex [62-64], potentially regulating its ATPase activity [65], during its functions in cellular signalling pathways that regulate transcription [62,66], the cellular heat shock response [60] and also in maintaining the cell cycle protein kinases Mik1, Wee1 and Swe1 [67]. This would suggest that this group functions as a control element within a wide range of cellular signalling pathways that include the regulation of the cell cycle.

The Cwc27 orthologous are present in 12 of the 16 fungi (Table 16A [See Additional File 1]; Group L). Not originally identified as members of the cyclophilin family, recent research has identified the presence of a degenerate PPIase domain in the N-terminus of these proteins followed by a C-terminal region rich in S/K-R/E residues that is similar to those observed in hnRNP-binding proteins [11,68]. All members were predicted by PSORT to be nuclear, which has been confirmed experimentally for ScCwc27 [10], however SpCyp7 has been reported to be found within the perinuclear space [11]. Both SpCyp7 and ScCwc27 have been reported to be a component of their respective Cdc5 complex [69], but their function, and those their orthologues, within this complex remains unknown. However, it does identify a role for this group within pre-mRNA processing. This is also in contradiction with the observed localization of SpCyp7, as the Cdc5 protein complex has been reported to be predominantly nuclear [10,70], indicating that SpCyp7 may have multiple functions within *Sz. pombe*.

Three groups are present in 10 out of the 16 fungi, with no members identified in *E. cuniculi *and all but two of the Saccharomycotina fungi (Table 16A [See Additional File 1]; Groups D, K & N). From the latter, *D. hansenii*, along with *Y. lipolytica*, has a member in both Groups D & N, or *E. gossypii*, in Group K. Very little is known about the functions of Group D besides the predominantly nuclear localization of SpCyp3, with a suggested role in pre-mRNA splicing [71], and its apparent presence in the spindle-pole bodies and/or microtubule organizing centres [11], both of which are contrary to the groups PSORT predicted cytoplasmic localization for all members except DhCyp2 which was predicted to be nuclear (Table 16A [See Additional File 1]), suggesting that the presence of this group in the nucleus is due primarily to their association with other factors. Again, very little is known about the functions of Group N, all of whose members are predicted to be cytoplasmic by PSORT, with the exception of DhCyp9 and UmCyp8 which were predicted to be nuclear, and share the presence of WD40 motifs in their N-terminal region. This motif is found in all eukaryotes, but not in prokaryotes, in a large variety of proteins that share no obvious commonality in their functions [72]. Finally, Group K members share the presence of a C-terminal RNA Recognition Motif (RRM), which is found in Metazoan protein factors involved in constitutive pre-mRNA splicing and alternative splicing regulation [73]. Again, very little is known about this group in fungi beyond the highly specific nuclear localization of SpCyp6 [11] and a putative role in cell morphogenesis, cortical organization and nuclear reorganization [74]. The localization of SpCyp6 is in agreement with the PSORT predicted localization of all members of this group except for EgCyp5 and DhCyp6 which were predicted to be cytoplasmic, indicating that their targeting to the nucleus may also be due to an association with other targeting factors, presumably during their functions within the pre-mRNA processing complexes.

One group is found in 9 of the 16 fungi, with no members identified in *E. cuniculi*, and all of the Saccharomycotina fungi with the exception of *Y. lipolytica *(Table 16A [See Additional File 1]; Group M). Half of its members are predicted by PSORT to be nuclear (RoCyp14, SpCyp8, YlCyp8, & UmCyp7) whilst the other half are predicted to be cytoplasmic (AnCyp9, AfCyp9, NcCyp6, GcCyp7, & CnCyp11). This difference is grouped by taxa, suggesting that there may be a difference in function between them despite the high sequence homology they all share [See Additional File 8] or that their function is both within the cytoplasm and nucleus and that this targeting difference is a response to changes in their target factors that in the latter group can transport them into the nucleus, but not in the former. They all possess an N-terminal U-Box motif, which is reported to be a modified RING-finger motif involved in protein:protein interactions that has been primarily identified in proteins involved in the ubiquitin/proteasome system [75]. As with the previous groups, very little is known about the functions of this group within these fungi beyond the predominantly nuclear localization reported for SpCyp8 [11], which is in agreement with its PSORT predicted localization.

All but four of the fungi have a PSORT predicted mitochondrial cyclophilin, the exceptions being *R. oryzae*, *E. cuniculi*, *Sz. pombe *and *U. maydis*. These cyclophilins are found spread between two othology groups (Table 16A [See Additional File 1]; Groups E & F) that are distinguished based upon sequence characteristics [See Additional File 8]. Group E is present in the all Saccharomycotina fungi, whilst Group F is present in all Pezizomycotina fungi and *C. neoformans*. In Group E, ScCpr3 has been reported as a mitochondrial cyclophilin [10] required for mitochondrial function under heat-stress [76] and as a protein folding chaperone within the mitochondria [59,77,78]. In Group F, NcCyp4 has both a mitochondrial and cytoplasmic isoform [50]. Mitochondrial NcCyp4 has been reported to cooperate with Hsp70 and Hsp60 within the mitochondrial matrix and whilst mitochondria lacking functional NcCyp4 efficiently imported preproteins into the matrix, the folding of the imported preprotein was significantly delayed [79]. It has also been reported to suppress the gating of the putative fungal mitochondrial permeability transition pore in a CsA sensitive manner [80]. No functions are yet known for its cytoplasmic isoform, but based on this information the mitochondrial isoform appears important for the maintenance of mitochondrial function. Interestingly, AnCyp3 and AfCyp4, whilst both showing a high degree of sequence homology to the other members of Group F [See Additional File 8], they were both predicted by PSORT to be cytoplasmic, unlike the other members of this group, due to the absence of a signal peptide [See Additional File 8]. *R. oryzae *has two cyclophilins (RoCyp5 & RoCyp6) that appear to be paralogues based on their sequence (data not shown) and also show a high degree of orthology to the members of mitochondrial Group E (data not shown), however they are predicted by PSORT to be cytoplasmic as no mitochondrial localization sequences were identified within their sequence. They could possess unknown mitochondrial targeting signals, but further investigation is required to confirm this.

Two groups are present only in *R. oryzae*, the Pezizomycotina fungi, and the Basidiomycota fungi with the exception of *U. maydis *(Table 16A [See Additional File 1]; Group A) or *N. crassa *(Group C). All Group A members are predicted to be cytoplasmic by PSORT, however SpCyp1 has been reported to be predominantly nuclear [11] and to function within a broad range of SNW/SKIP signal transduction pathways involved in cell proliferation and differentiation [81], suggesting that it is its interaction with factors within these pathways that cause its targeting to the nucleus. Group C members are predicted by PSORT to be cytoplasmic, but nothing is currently known about the functions of this group.

Group J is found solely within the Saccharomycotina fungi (Table 16A [See Additional File 1]). This is a second TPR-containing cyclophilin group whose members exhibit a very high degree of sequence homology with their respective member of the other TPR-containing group (Group I; data not shown), and in the case of ScCpr7, to also interact with Hsp90 [61,82,83], suggesting that they share a similar role within cellular signalling pathways. Like Group I, members of this group are predicted by PSORT to be cytoplasmic, in agreement with the reported localization for ScCpr7 [10], with the exception of CaCyp6 which was predicted to be nuclear, supporting possible conserved functions.

Finally, Group H is also found solely within the Saccharomycotina fungi with the exception of *Y. lipolytica *(Table 16A [See Additional File 1]). ScCpr4 has been reported to localise to the endoplasmic reticulum [10,14], function within the secretory pathway [59], possess a putative transmembrane domain and is induced by heat shock and tunicamycin. This would suggest that this group has a role within the vesicular protein folding pathways as a folding catalyst or chaperone.

The dendrogram showing the putative evolutionary relationship of the fungal cyclophilins (Figure 1A) shows good agreement with the groups identified by BLAST analysis (Table 16A [See Additional File 1]), and it may also allow us to better understand the evolution of the eleven individual cyclophilins identified in four of the fungi. Interestingly, we would have expected the uni-domain cyclophilins to have evolved first, followed by the larger multi-domain cyclophilins as the cells become more complex. However, the pattern in the dendrogram suggests that the initial divergence separated the ancestor of the TPR containing Groups I & J from the ancestor of the other groups. The other multi-domain groups are found to evolve amongst the other uni-domain cyclophilins, of which many have evolved from the ER Group G despite their predominant PSORT predicted cytoplasmic and nuclear localizations. Only uni-domain Groups A, E & F are observed to evolve on a separate branch which they share with four individual cyclophilins (RoCyp5, RoCyp6, RoCyp11, & CnCyp10). This would suggest that the functions of Group I became important early in the evolution of the fungi, with the other multi-domain groups evolving as the cells became more complex, and/or as the uni-domain cyclophilins evolved functions that required a second domain.

It is of note that in many cases the evolution of the members of each group does not follow that of their respective fungi. This suggests that there may be variable factors within these fungi that are driving their evolution. Some fungi share these factors in common despite not sharing a close evolutionary history, whilst others that share a close evolutionary relationship do not. This would result in variable evolutionary pressures on individual members of the group, leading to clustering in the dendrogram away from that of the evolutionary history of the host fungus but which may be representative of shared evolutionary factors.

Despite the observed clustering of most groups identified by BLAST analysis within the dendrogram, two groups do however show fragmentation; Group B, which is found in four distinct parts of the dendrogram, and Group G, which is found in two. This would suggest a complex evolutionary history for these two groups, but may also indicate that some members that have been identified by BLAST analysis may not be true orthologues of these groups. In most cases, the parent fungi of these outliers does not have a member in the group to which these outliers appear associated, and these groups also appear related to the group identified by BLAST analysis. This could indicate that there are selective pressures driving these outliers to perform some functions of the group to which they appear associated, slightly increasing their homology to this group and away from their BLAST identified group, but not enough to be distinguished by BLAST analysis, resulting in their observed clustering in the dendrogram.

The initial divergence separated the discrete branch that contains the ubiquitous TPR containing human cyclophilin 40 orthlogues (Group I) and their Saccharomycotina specific paralogues (Group J), and the human USA-CyP orthologues (Group D), implying a shared evolutionary history, from the other groups (Figure 1A). All are predicted by PSORT to be cytoplasmic, which is supported by experimental studies which also showed an appreciable nuclear component for members of each group [10,11]. It is interesting that the uni-domain members of Group D, whose function appears vastly-different from those of Groups I & J, appears to have evolved from a common ancestor with Group J.

The evolution of the cytoplasmic human cyclophilin A orthologues (Group B; Figure 1A) and mitochondrial cyclophilins (Groups E & F; Figure 1A) appears on a discrete second branch, which on a wider aspect is shared with four individual cyclophilins, three within *R. oryzae *(RoCyp5, RoCp6, & RoCyp11) and one from *C. neoformans *(CnCyp10). RoCyp5 & RoCyp6 appear to possibly be part of mitochondrial Group E based on sequence homology (data not shown) although they are predicted by PSORT to be cytoplasmic due to the absence of any signal sequences, and RoCyp11 & CnCyp10 appear to be related based upon sequence homology (data not shown), but not sufficiently to be called orthologues. The presence of Groups B, E, & F on this shared branch would suggest a close evolutionary history, which is supported by the observed dual cytoplasmic and mitochondrial nature of the NcCyp4 gene [50] and the appreciable sequence homology between Groups B, E, & F (data not shown). NcCyp4 shares a sub-branch with GzCyp5, AnCyp3 & AfCyp4, suggesting that these other cyclophilins may, or may have, exhibited this dual nature, which is supported in the case of GzCyp5 by the presence of a putative N-terminal signal peptide [See Additional File 8]. There is no clear separation of these groups into discrete sub-branches, with YlCyp1, a member of Group B, found within Group E on a sub-branch it shares with YlCyp3, its Group E member, indicating that their evolution may have been more restricted or more recent than the other members leading to their greater sequence homology. Interestingly, AfCyp2, a member of Group B, is found on its own sub-branch that shares a major branch with Groups D, I, and J, suggesting a different evolutionary path for this cyclophilin from the other Group B members. EcCyp1, another Group B member, is also found on a separate sub-branch, which it shares with CnCyp6, an individual cyclophilin that appears to be a paralogue of CnCyp3, its Group B member, suggesting that EcCyp1 may have adapted to perform functions for which *C. neoformans *has evolved a second cyclophilin to perform. Also interesting is that RoCyp1 is found within the Group J members on a separate branch, whilst RoCyp2, an apparent paralogue of RoCyp1, is found on the same branch as Groups B, E, & F. Based on sequence homology RoCyp1 would be called the *R. oryzae *orthologue of Group B [See Additional File 8], however its PSORT predicted nuclear localization is in contrast to the cytoplasmic localization predicted for the other members of Group B. It's observed clustering in a different branch of the dendrogram could indicate that it has a divergent function from this group, and that RoCyp2 may be the true Group B member despite exhibiting a lower sequence homology towards its members. RoCyp2 also shares its branch with RoCyp10, a fungal cyclophilin that is unique in that is possesses an N-terminal RRM domain. The presence of an RRM is only observed in one other group (Group K) where it is found in their C-terminal domain. Nuclear Group K is found on a separate branch from cytoplasmic RoCyp10 (Figure 1A), suggesting that these two RRM containing cyclophilin groups have evolved separately to perform their specific functions which may be within different compartments of the cell.

The final major branch sees all other groups evolve from a common ancestor that appears to begin with the precursor to ER Group G (Figure 1A), which itself appears to have evolved in three phases that are not restrained by the evolutionary history of the fungi. The initial branch contains all the members from the Pezizomycotina fungi, as well as CnCyp7, SpCyp4, RoCyp8, and interestingly YlCyp4, a member of the Saccharomycotina taxa. The other members from the Saccharomycotina taxa appear to evolve in the remaining two branches, with CaCyp3 evolving with DhCyp4 and interestingly UmCyp3, a member of the Basidiomycota taxa, whilst ScCpr5, EgCyp3, KlCyp3, and CgCyp3 all evolve on the final branch. This pattern of evolution is hard to explain. It may involve the convergent evolution of some cyclophilins to perform a common function present in some, but not all, of the fungi, or it could indicate the presence of shared evolutionary pressures within subsets of the fungi. An individual cyclophilin within *R. oryzae*, RoCyp9, is also seen to evolve on the first branch along with its closely related Group G member, RoCyp8, which may indicate that a recent gene duplication has occurred. *S. cerevisiae *also has an individual cyclophilin, ScCpr2, that is present on the same major branch as its Group G member, but it appears more distantly related (data not shown) which would indicate that if gene duplication did occur, it happened earlier than the *R. oryzae *duplication.

It is fascinating that the remaining functionally divergent groups that are predominantly present in the cytoplasm and nucleus all appear to have evolved from the ER Group G. The initial divergence from Group G appears to have separated the evolution of two cytoplasmic groups, uni-domain Group A and the WD40 containing Group N, with the former appearing to evolve on a discrete branch that shares a common ancestor with the later, from the other remaining groups. Both appear vastly different in putative function, with Group A being small uni-domain predominantly nuclear cyclophilins whilst Group N are large multi-domain putatively cytoplasmic cyclophilins, suggesting that Group N gained the WD40 domain after their divergence from their common ancestor.

The next divergence in the companion branch sees the evolution of the ER Group H on two discrete branches, which are shared with ScCpr8, an individual cyclophilin in the budding yeast that whilst it is not found in the ER, it has been reported to be a membrane bound protein [14]. This is not far removed from that of an ER protein, and this may have come about through the loss of its ability to loose its signal peptide. Interestingly, EcCyp2, a member of Group G, is found within these branches rather than on the branches with its fellow group members. This could indicate a degree of divergent evolution of this cyclophilin to fulfil the needs of both Group G and Group H within its parent fungi. The final divergence led to the evolution of the nuclear RRM containing Group K and the related Cwc27 orthologues (Group L), both of which appear to be involved in mRNA processing, on a discrete branch from the cytoplasmic uni-domain cyclophilins of Group C and the U-box containing predominantly nuclear Group M, which themselves are also found to evolve on a shared branch.

### FKBP orthology

Table 16B [See Additional File 1] shows that the only FKBP group to have members in all these fungi, with the exception of *E. cuniculi *which has no FKBPs in its genome, are the human FKBP12 orthologues (Group A). ScFpr1 has been reported to be cytoplasmic [10], in agreement with their PSORT predicted localization with the exception of UmFKBP2 which was predicted to be nuclear. SpFKBP12 has been reported to have an important role in the early steps of the fission yeast sexual development pathway but it is not essential for normal growth [84], and mutant cells that lack CaFKBP1 [85] and CnFKBP1 [86] have also been reported to be viable under normal conditions. Finally, ScFpr1 has a reported regulatory role within the homoserine synthetic pathway where disruption of its function perturbs the aspartokinase feedback inhibition by threonine resulting in the toxic accumulation of aspartate β-semialdehyde, the substrate of homoserine dehydrogenase [87]. Group B (Table 16B [See Additional File 1]) and the individual FKBP AnFKBP2 appear to be closely related to Group A based upon sequence homology (data not shown), but nothing is presently known about the functions of these FKBPs.

**Figure 2:**
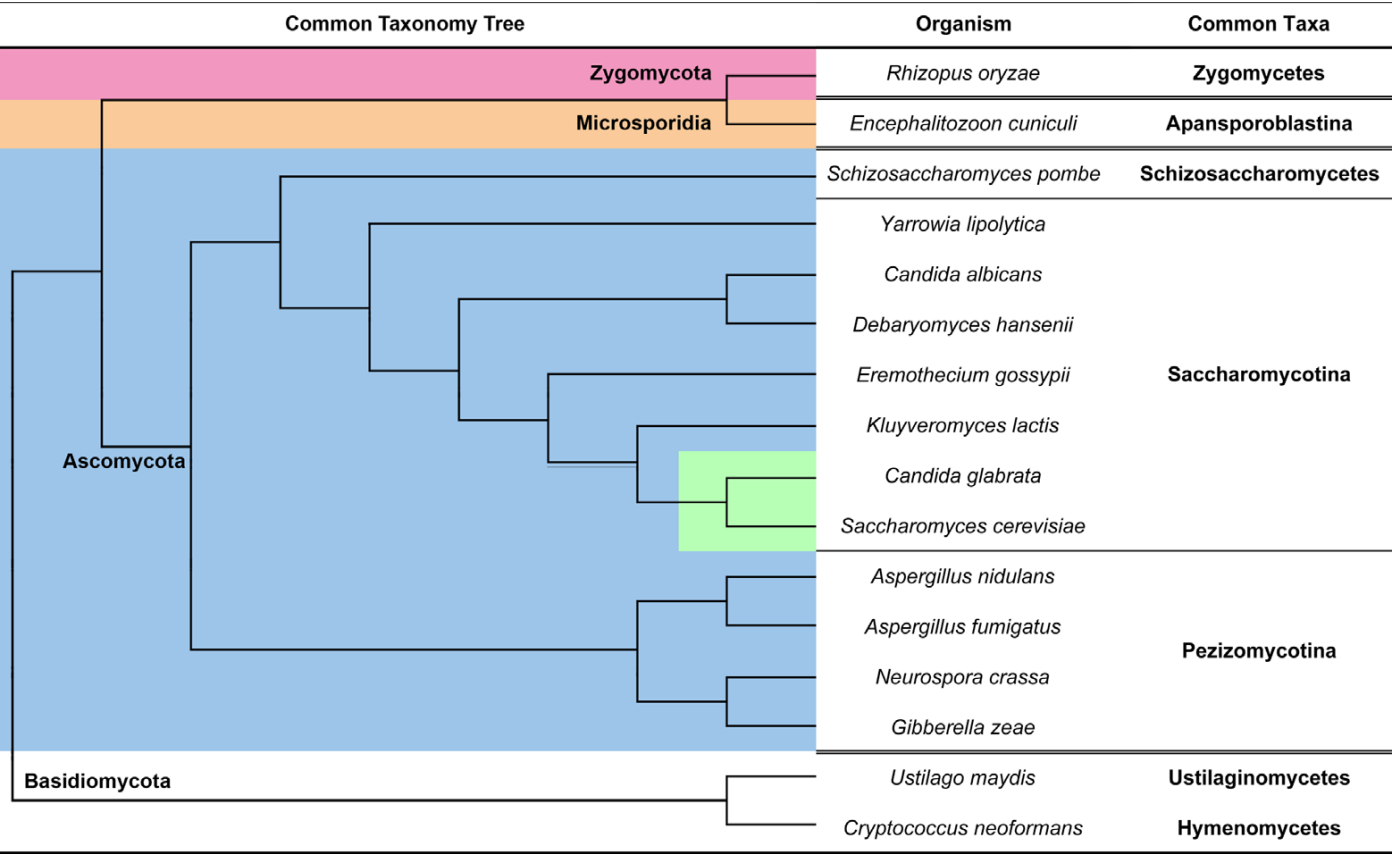
**Common taxonomy tree depicting the evolutionary relationship of the fungi: **The Ascomycota are ordered as previously reported [163]. Branches highlighted in green indicate those genomes that are known to have undergone whole genome duplication (WGD) during their evolution [119]. Branch lengths are not proportional to evolutionary distances.

The only other group to show conservation in many of these fungi is that of Group C (Table 16B [See Additional File 1]). Members are present in all but *E. cuniculi*, *Sz. pombe*, *E. gossypii*, *G. zeae*, and *C. albicans*, despite the closely related fungus *D. hansenii *(Figure 2) having a member. *R. oryzae *has two closely related FKBPs, RoFKBP2 & RoFKBP3, that appear to be paralogues and show a high degree of homology with the other members of Group C (data not shown). ScFpr2 has been reported to be resident within the ER [10,88], and NcFKBP3 has been reported to be synthesized as a precursor protein with a cleavable signal sequence and a C-terminal HNEL endoplasmic reticulum (ER) retention signal, strongly suggesting that NcFKBP2 is an ER resident protein [89]. This is in agreement with the PSORT predicted ER localization for all members of this group with the exception of CgFKBP2 and NcFKBP2 which were predicted to be cytoplasmic due to the absence of an N-terminal signal peptide. In addition, NcFKBP3 was reported to have a carboxyterminal domain that has an amino acid composition biased towards charged residues that is predicted to form an amphipathic α-helix [89], which could indicate that NcFKBP3 will function at the surface of plasma membranes [90].

The remaining groups identified within the FKBP repertoires appear specific to a subset of the fungi compared here. Group I has been identified solely within the Pezizomycotina fungi (Table 16B [See Additional File 1]), however nothing is known about the members of this group besides their PSORT predicted cytoplasmic localization (Table 16B [See Additional File 1]). Group D was identified only within *N. crassa *and *G. zeae *and both members were predicted by PSORT to localize to the ER, but their functions remain unknown. Group F (Table 16B [See Additional File 1]), whose members are predicted by PSORT to all be nuclear, is found only within some of the Saccharomycotina fungi, the exceptions being *Y. lipolytica*, *D. hansenii*, and *C. albicans*. Both *S. cerevisiae *and *C. glabrata *have a second FKBP that shares a high degree of homology with their member of Group F (Table 16B [See Additional File 1]; Group G), and ScFpr4 has been reported to share similar properties with ScFpr3. Both ScFpr3 [10,91-93] and ScFpr4 [10,76,94] have been identified as nuclear, and have been reported to suppress defects seen in the absence of the E3 ubiquitin ligase TOM1 [94]. ScFpr3 has also been reported to maintain recombination checkpoint activity through the control of protein phosphatase 1 [95], a critical function during meiosis, and the phosphorylation state of ScFpr3 is important for correct growth [96] but has no affect on its localization [97], suggesting the phosphorylation state of ScFpr3 is important for its cellular function. Members of Group H are present only within *C. albicans *and *D. hansenii*, with the former appearing to also have a paralogue of its member, CaFKBP2, and there is a closely related FKBP within *Y. lipolytica*, YlFKBP3.

Finally, Group E is found in four of the 16 fungi (Table 16B [See Additional File 1]); *R. oryzae*, *Sz. pombe *and both of the Basidiomycota fungi. Three members are predicted by PSORT to be nuclear (RoFKBP4, SpFKBP39, & UmFKBP3), however CnFKBP3 is predicted to be ER due to the presence of an N-terminal signal peptide, suggesting a possible difference in function despite its high sequence homology towards the other members of this group [See Additional File 8]. SpFKBP39 has been reported as nuclear [98] and to have a role in chromatin remodelling involved in ribosomal DNA silencing through a potential role as a histone chaperone [99].

The dendrogram for the evolution of the FKBPs (Figure 1B) shows good agreement with the groups identified by BLAST analysis (Table 16B [See Additional File 1]). It is interesting to note that the dendrogram suggests that the evolution of the uni-domain FKBPs (Groups A, B, C, & D) occurred first, with the FKBPs that possess a second charged domain (Groups E, F, G, & H) evolving later in their history. The exception to this is the evolution of RoFKBP5, the only fungal FKBP with a TPR domain in this comparison, which appears before the evolution of the other multi-domain FKBPs. Orthologues of RoFKBP5 are not present in any of the other fungal genomes that are currently available (data not shown), suggesting that it may be restricted to a specific fungal taxa. Without further orthologues the evolutionary history of this group cannot be fully explored, as RoFKBP5 could have either evolved recently in *R. oryzae*, or at an earlier point in a recent ancestor.

The FKBPs all appear to have evolved from a single ancestor that was related closest to Group A, the cytoplasmic FKBP12 orthologues. These are seen to evolve first but in four different phases that appear as the other groups begin to evolve. UmFKBP2 and YlFKBP1 are found on the earliest branch, suggesting that these are the earliest precursors of this group, which does not agree with the evolution of the fungi as both are members of different taxa (Figure 2). The next three divergences separate the evolution of the remaining Group A members from the other FKBPs. First to evolve were the Saccharomycotina Group A members on their own discrete branch, followed by the remaining Group A members SpFKBP12, RoFKBP1, CnFKBP1, AnFKBP1 & AfFKBP1, and finally NcFKBP1 and GzFKBP1. This pair shares a branch with the Group B members in *A. nidulans *and *A. fumigatus*, which both appear to be closely related to their Group A members at the sequence level (data not shown), and interestingly RoFKBP5, an individual cyclophilin that is unique in possessing a TPR domain.

The next divergence led to the evolution of AnFKBP2, an individual cytoplasmic FKBP that appears closely related to its Group A member, from the other groups. The evolution of ER Group C came next, whose members have all evolved on a discrete branch that separates the Saccharomycotina members and *R. oryzae*, which is normally found with the other fungal taxa, on one sub-branch and the other fungal taxa, who share their sub-branch with RoFKBP3, an individual ER FKBP that appears to be a paralogue of its Group C member (data not shown), and the two members of the final ER group, Group D, on the other.

Next came the evolution of the Group E members, which occurred in two phases. All but the *Sz. pombe *member evolve on a discrete branch, with SpFKBP39 found on a different branch it shares with SpFKBP39a, which appears to be a paralogue of SpFKBP39 (data not shown) that has been reported to be nuclear [98]. This suggests that *Sz. pombe *has evolved a second FKBP to perform the functions of Group E. All members of Group E were predicted by PSORT to be nuclear, with the exception of CnFKBP3 which was predicted to be in the ER due to the presence of an N-terminal signal peptide. Their clustering in the dendrogram would indicate that any divergence in function was relatively recent as their sequences have not yet significantly diverged.

Cytoplasmic Group I, whose members are restricted to the Pezizomycotina fungi, is found to evolve on the companion branch from the groups that include the SpFKBP39 branch. Finally, the evolution of the nuclear Groups F, G & H are all found on the same branch, indicating a close evolutionary relationship. The evolution of Group F is observed on a discrete branch that shows a close evolutionary relationship with both members of Group G, which is supported by their high sequence homology (data not shown). Finally, Group H shares its discrete branch with two individual FKBPs, cytoplasmic YlFKBP3 and nuclear CaFKBP2, both of which appear to be related to Group H at the sequence level, but not to each other (data not shown). It also appears that the divergence of CaFKBP2 and CaFKBP3 is relatively recent, as they appear together on the same discrete branch.

### Parvulin orthology

Compared with the cyclophilin and FKBP repertoires, the parvulin repertoires are relatively small. All the compared fungi share a single parvulin in common with a second parvulin only found within the Pezizomycotina fungi (Table 16C [See Additional File 1]). Members of both groups are predicted by PSORT to be nuclear, with the exception of one member of Group A (GzPar1), and two members of Group B (EgPin1 & AnPin1), suggesting that the direct targeting of these groups to the nucleus is either not essential and/or that their interaction with target factors recruits them into the nucleus, or that we do not fully understand all of the fungal NLS.

The sole parvulin they all share in common is that of the human Pin1 orthology group (Table 16C [See Additional File 1]; Group B). SpPin1 is reported to be it a positive regulator of the cell cycle control proteins Wee1 and Cdc25 [19]. ScEss1 is reported to be nuclear and involved in transcription through an interaction with the C-terminal domain of RNA polymerase 2 [10,100-105] downstream of its phosphorylation repsonse to DNA damage [106], and in cell cycle regulation [19]. Interestingly, ScEss1 [16] and CaEss1 [18] have been reported as essential genes, whereas SpPin1 [19] and CnEss1 [20] have been reported to be non-essential, although that latter has been reported to be required for virulence. Cross-talk between ScEss1 and ScCpr1 [20,55], a member of cyclophilin Group B, has been reported to modulate the activity of the Sin3-Rpd3 complex with excess histone deacetylation causing mitotic arrest in ScEss1 mutants [53] and CnEss1-null mutants have been reported to be hypersensitive to CsA [20], suggesting a cyclophilin-mediated redundancy mechanism. Disruption of ScEss1 can be complemented by DmDodo [21] and the plant *Digitalis lanata*'s Par13 [107], which lacks the WW domain conserved in the other proteins, indicating a conserved functionality may exist between all Pin1 orthologues that is essential in some but not all organisms under normal growth conditions and only requires the rotamase domain. CaEss1 is reported as essential for growth and the reduction in its dosage or activity blocks morphogenetic switching between the hyphal and pseudohyphal forms under certain conditions [18]. Structural studies have shown that whilst CaEss1 has the same overall structure as its human orthologue, hPin1, it's altered α-helical linker results in a rigid juxtaposition of the WW and rotamase domains that gives the two domains of CaEss1 a distinct orientation from that of hPin1, eliminating the hydrophobic pocket between the domains that was identified as the main substrate recognition site [108]. NcPin1 is unique among the known eukaryotic parvulins in containing a polyglutamine stretch between the N-terminal WW domain and the C-terminal rotamase domain [109], indicating that it may have a specialized function within this fungi. So far it would appear that some, but not all, of the functions of this group are essential, with varying degrees of redundancy present in some fungi, and structural studies would suggest that whilst these functions require one or both of the domains present in its members, it is unlikely that they will act in synergy given that their orientation appears to vary between the different members.

The final parvulin orthology group was identified only in the Pezizomycotina fungi (Table 16C [See Additional File 1]; Group A). Whilst nothing is presently known about this group in fungi, it is interesting to note that its members appear to all be orthologues of the second parvulin group previously only identified within the Metazoa that includes human Par14 [68,110]. This indicates that the evolution of Group A must have occurred prior to the evolutionary split of the unicellular fungi from the ancestral cell that went on to form the Metazoan eukaryotes.

As we would expect, the dendrogram for the parvulins (Figure 1C) shows the evolution of Group A occurs on a discrete branch from those of Group B, but interestingly it shares a branch with EcPin1, a member of Group B. The dendrogram shows that the members of Group B have not evolved within their specific taxa, but rather they have a shared evolution, as inferred by the lack of clustering of the members from each taxa onto the same branches. There are four sub-branches that form Group B, with CaEss1, DhPin1 and RoPin1, appearing the most removed of all the members, with the remaining groups forming on two branches, with UmPin1 and CnEss1 evolving form the same branch as Group A and appearing related to EcPin1. Interestingly, the three members for which structural studies have been reported are found on separate branches, indicating that the clustering of the Group B members in the dendrogram may represent the three different tertiary structural conformations that they represent.

## Discussion

The number of PPIases within these fungal repertoires varies, with *E. cuniculi *having the smallest repertoire with 3 and *R. oryzae *the largest with 22, which is close to that of *D. melanogaster *and *C. elegans *[68]. The number of PPIases in a given repertoire appears associated with the number of genes in the genome of the fungus, averaging 1.9 PPIases per 1000 genes. Some fungi were found to be more PPIase rich than others, such as *Sz. pombe *and *C. neoformans *with around 2.6 PPIaess per 1000 genes, whilst others were found to be relatively PPIase poor, such as *G. zeae *and *R. oryzae*, with 1.06 and 1.26 PPIases per 1000 genes, respectively. The greatest variation was observed in the number of cyclophilins, where on average there were between 6 and 13 present, whilst the number of FKBPs were fairly constant, with between 3 and 5 present, and on average just a single parvulin was present. The exception was the presence of a second parvulin within the Pezizomycotina fungi, whose parvulin repertoires resemble those of the Metazoa [68]. *E. cuniculi *was unique is possessing no FKBPs, whilst retaining a member for both of the highly conserved cyclophilin groups (Table 16A [See Additional File 1]; Groups B & G) and the sole highly conserved parvulin group (Table 16C [See Additional File 1]; Group B).

We have shown that only two cyclophilin groups, cytoplasmic Group B and endoplasmic reticular Group G, are present in all of the fungi. Another predominantly cytoplasmic group is found in all the fungi with the exception of *E. cuniculi *(Group I), which has no further cyclophilins in its repertoire. Five groups were found to be restricted to *C. neoformans*, *Sz. pombe R. oryzae*, the Pezizomycotina fungi, and were also present in some of the Saccharomycotina fungi. Nuclear Group L was present in four of the Saccharomycotina fungi; *S. cerevisiae*, *D. hansenii*, *E. gossypii*, and *Y. lipolytica*. Three groups were present in two Saccharomycotina fungi; Cytoplasmic Groups D & N was present in *Y. lipolytica *and *D. hansenii*, and nuclear Group K was present in *D. hansenii *and *E. gossypii*. Finally, predominantly nuclear Group M was only present in one of the Saccharomycotina fungi, *Y. lipolytica*. Four groups were found to be absent in the Saccharomycotina fungi. As with the above groups, cytoplasmic Group A is restricted to the Pezizomycotina fungi, *C. neoformans*, *Sz. pombe *and *R. oryzae*, but in this case it was absent in the Saccharomycotina fungi. Similar to Group A, cytoplasmic Group C was restricted to *U. maydis*, *R. oryzae Sz. pombe*, and the Pezizomycotina fungi with the exception of *N. crassa*. Finally, mitochondrial Group F was restricted to the Pezizomycotina fungi and *C. neoformans*. Three groups were also found to be restricted to the Saccharinycotina: Mitochondrial Group E, endoplasmic reticular Group H, and cytoplasmic Group J.

Eleven individual cyclophilins were identified, six of which are present in *R. oryzae*; RoCyp2, RoCyp5 and RoCyp6 which appear to be paralogues, RoCyp9, RoCyp10, which is the only known fungal cyclophilin with an N-terminal RRM, and RoCyp11. All were predicted to be cytoplasmic with the exception of RoCyp9, which was predicted to be endoplasmic reticular, and RoCyp11, which was predicted to be nuclear. Of the remaining five, two were present in both *S. cerevisiae *(ScCpr2 & ScCpr8) and *C. neoformans *(CnCyp6 & CnCyp10), and one was present in *Y. lipolytica *(YlCyp5), with the remainder of the fungi having no unique cyclophilins in their repertoire. Of these last five, ScCpr2 has been found to function in the ER, ScCpr8 has been reported to associate with membranes, YlCyp5 is predicted to be nuclear, and CnCyp6 is predicted to be cytoplasmic.

We have found that the cyclophilins appear to have a wide range of functions within the cell, but there are several conserved themes within these functions. We have the expected roles in protein folding and chaperoning both within the ER (Groups G & H) and the mitochondrion (Groups E & F). For the former, only one group was found to be present within all fungi (Group G) whereas Group H was found solely within all but one of the Saccharomycotina fungi, *Y. lipolytica*, suggesting that these fungi require an increased amount of PPIase-mediated protein folding within their ER. Given their small genome size (Table 15), this is presumably an adaptation to environmental challenges predominantly affecting this taxa. The mitochondrial groups are found separated between two separate taxa, Group E is present solely within the Saccharomycotina fungi whilst Group F is found solely within the Pezizomycotina fungi and *C. neoformans*, suggesting a divergence in their functions within the mitochondria. Group F also has two different types of members; we have the dedicated mitochondrial cyclophilins in *C. neoformans*, and presumably *A. nidulans *and *A. fumigatus *despite their PSORT predicted cytoplasmic localization, and those in *N. crassa *and *G. zeae *that have two isoforms that target them to the cytoplasm (Group B) or mitochondrion (Group F), which may shed light on the evolutionary history of these groups. Interestingly, no mitochondrial cyclophilins were identified in *Sz. pombe*, *E. cuniculi *and *U. maydis*, which given that they are spread throughout the fungal evolutionary tree is easiest explained by gene deletion. *R. oryzae *also had no identified mitochondrial cyclophilin, but it does have two cyclophilins that show a high degree of sequence homology to mitochondrial Group E but which are predicted by PSORT to be cytoplasmic due to the absence of any signal sequences.

We have also found three groups involved in cellular signalling pathways (Groups A, I, & J). Whilst Group A appears to function within the SNW/SKIP pathways involved in cell proliferation and differentiation within all the fungi except *E. cuniculi*, *U. maydis*, and the Saccharomycotina fungi, Groups I & J appear to function as part of the Hsp90 complex in hormone signaling and cell cycle pathways in all fungi except *E. cuniculi*, or in the case of Group J, only within the Saccharomycotina fungi, suggesting that this taxa has additional divergent pathways that require an extra cyclophilin-Hsp90 chaperone. Overall, these cyclophilin groups appear to function within cellular signaling pathways involved in regulating the cellular growth response to external signals.

Finally, we have three groups that appear to have a role within pre-mRNA processing (Groups D, K, & L), that are largely absent from the Saccharomycotina fungi and *E. cuniculi *(Table 16A [See Additional File 1]). The presence of an RRM in members of Group K suggests that they function in the recruitment of the RNA related to cell morphogenesis, cortical organization and nuclear reorganization into the pre-mRNA processing complexes, whereas members of Group L have been found present within the Cdc5 complex and along with Group D, are believed to function within pre-mRNA splicing.

There are also some groups with unique or less defined functions within these fungal repertoires. Group B is present in all the fungi and its members appear to function in a wide range of pathways from the control of protein activity, transcription, translation and the cell cycle, to the vesicular transport of proteins and the control of fungal virulence. Group M is found in all the fungi except *E. cuniculi *and the Saccharomycotina fungi, with the exception of *Y. lipolytica*, and its members are believed to function in the proteosome degradation pathway, potentially recruiting target proteins for degradation based upon the presence of a U-box domain in their sequence. Group N is present in the same fungi as Group M, but also *D. hansenii*, and its members appear to function within protein complex(s) due to the presence of a WD40 motif in their sequence. However, both groups require further investigation to elucidate their functions within the cell. Also, there is one group (Group C), which lacks any secondary domains with which to infer a putative function and at present, there is nothing known about its members besides its presence in all fungi except the Saccharomycotina fungi, *E. cuniculi*, *Sz. pombe*, and *N. crassa*.

Only a single FKBP group was present in all fungi, with the exception of *E. cuniculi *as it possessed no FKBPs, that being cytoplasmic Group A which shows orthology to human FKBP12 (Table 16B [See Additional File 1]; Group A). ER Group C was found to be in all fungi with the exception of *Sz. pombe*, *E. gossypii*, *G. zeae*, and *C. glabrata*. Three groups are restricted to the Saccharomycotina fungi. Nuclear Group F is present in all members except for *Y. lipolytica*, *D. hansenii*, and *C. albicans*. Nuclear Group G is only found in *S. cerevisiae *and *C. glabrata *and its members exhibit high sequence homology to members of Group F, and nuclear Group H is found only in *C. albicans *and *D. hansenii*. The remaining four groups are not found in the Saccharomycotina fungi, of which three are restricted to the Pezizomycotina fungi; Cytoplasmic Group I, ER Group D, which is only present in *N. crassa *and *G. zeae *but neither of the *Aspergillus *fungi, and Group B, which is found only within the *Aspergillus *fungi and appears closely related to Group A. Finally, nuclear Group E was restricted to *R. oryzae*, *Sz. pombe*, and both of the Basidiomycota fungi, *U. maydis*, and *C. neoformans*.

There were six individual FKBPs identified as present within five of the fungi (Table 16B [See Additional File 1]). Two were present within *R. oryzae*; RoFKBP2 appears to be a paralogue of RoFKBP3, a member of ER Group C, and it also shares a PSORT predicted ER localization. RoFKBP5 is predicted by PSORT to be cytoplasmic and it is the only known fungal FKBP to contain a TPR domain. The remaining four fungi each have just a single individual FKBP. *A. nidulans *has an extra cytoplasmic FKBP, AnFKBP2, that appears to be closely associated with Groups A & B, and *Sz. pombe *contains nuclear SpFKBP39a, which appears to be a paralogue of its Group E member (SpFKBP39). Both *C. albicans *(CaFKBP2) and *Y. lipolytica *(YlFKBP3) have an FKBP that shows appreciable sequence homology to nuclear Group H, with CaFKBP2 sharing this nuclear localization, whereas YlFKBP2 is predicted by PSORT to be cytoplasmic.

We do not find the same clustering of functions with the FKBPs as were found with the cyclophilins. There are two ER groups (Groups C & D), but whilst it appears that they will be involved in the vesicular protein folding pathways this has not been confirmed experimentally, and they are present only within a subset of the fungi (Table 16B [See Additional File 1]), suggesting that their function is not universally conserved. Group A was found to be in all fungi and they appear to have a wide range of roles within the sexual development of the fungi and also in other pathways where it appears likely that their role is in maintaining the correct folding of the protein components of these pathways. Group B appears closely associated with Group A based on sequence homology (data not shown), although no functions are yet known for its members. Nuclear Groups F & G are both found only within a subset of the Saccharomycotina fungi (Table 16B [See Additional File 1]) and they appear functionally linked. Members of Group F appear to have a role within recombination checkpoint maintenance. A member of nuclear Group E has been reported to function within chromatin remodelling associated with DNA silencing, and its presence in only *Sz. pombe*, *R. oryzae *and the Basidiomycota fungi implies a specific rather than generalized function. At present, nothing is known about cytoplasmic Group I, which is found solely within the Pezizomycotina fungi, or nuclear Group H, which is found only within *C. albicans *and *D. hansenii*.

Both the cyclophilins and FKBPs are found similarly distributed throughout the cells organelles, but the cyclophilins are the only family found within the mitochondria. However, the apparent evolutionary pattern of the cyclophilins and FKBPs was found to be different. Whilst the single domain FKBP groups appear to have evolved prior to the larger multi-domain FKBP groups (Figure 1B), suggesting that the latter became important as cellular complexity increased, the same is not true for the cyclophilins (Figure 1A). The TPR containing multi-domain cyclophilin groups appear to have evolved very early on, suggesting that they have a very important and primal role within the cell, whereas the other multi-domain cyclophilin groups have evolved throughout the dendrogram at varying stages in the evolution of the fungal cyclophilin groups. This would suggest that the factors that drove the evolution of the multi-domain cyclophilin groups are, at least in part, different from that of the multi-domain FKBPs. Interestingly, in some instances we find single domain cyclophilin groups evolving from the branches of multi-domain groups. This would suggest that as the multi-domain cyclophilins evolved along with the cell, in some instances they were able to adapt to perform some functions without the secondary domain, and in such instances were able to spawn a new group to perform these functions as they continued to evolve new ones. Alternatively, there may have been an evolutionary event in one of their targets, such as the creation of a duplication of a target protein that then evolved to require a subtle adaptation in the cyclophilin that could not be accommodated. In such instances, a gene duplication of the cyclophilin and the incorporation of this adaptation into one copy would allow the protein and its host fungi to continue their evolution. If this change also ends the requirement for the cyclophilins secondary domain, it would cease to be selected for which would allow for its loss at some point during the evolutionary history of this new group.

This observation would suggest that the evolution of the FKBP family may have been in response to the increasing complexity of the proteome of these fungi as they have evolved, which after a certain point required more than just the PPIase catalytic domain to perform their functions in some cases. However, the cyclophilins appear to have evolved both in response to the increasing complexity of the proteome of these fungi and to the increase in the complexity of the underlying biological pathways that allow the fungi to survive, implied by the sporadic nature of the evolution of the more complex cyclophilins.

The final and smallest of the PPIase families, the parvulins, had just a single representative in most of the fungi, that of the nuclear human Pin1 orthology group (Table 16C [See Additional File 1]; GroupB) whose members appear to have a wide range of functions within transcription and cell cycle regulation. Interestingly, a second nuclear member was present in the Pezizomycotina fungi that showed orthology to the human parvulin Par14 (Group A). Orthologues of Group A have only previously been identified in the Metazoa, and were thought to have evolved within the Metazoa, however their presence in the Pezizomycotina fungi brings a new perspective on their evolution. The position of the Pezizomycotina taxa in the evolutionary tree and the absence of any Group A orthologue in the other fungi does however create a mystery. There is no common ancestor shared between the Pezizomycotina taxa and the Metazoa that the former does not share with other fungal taxa. So if this group evolved prior to the divergence of both the Pezizomycotina and Metazoa from their common ancestor, why did the other fungi not retain this parvulin? Or, did these parvulins evolve separately within the Pezizomycotina and Metazoa by convergent evolution? This group is not present in any of the other fungal genomes currently available in the NCBI database (data not shown), making the further analysis of this group within the genomes of additional fungi as they become available imperative to understanding their evolution and role within the cell.

Taking into account both the evolutionary paths of the three different PPIase families, their conservation within the different fungi, and their known functions within the fungi, we would hypothesize that the parvulins and cyclophilins have evolved to perform conserved functions within the fungi. The parvulins appear to have primal functions, presumably retained from the bacterial lineages within which this PPIase family is the predominant group [6,111], to which they are now largely restrained, whilst the cyclophilins appear to have gained functions at varying stages during fungal evolution. However, the FKBPs appear to have evolved to fill the ever-changing niches within these constantly evolving fungi, presumably in response primarily to the evolutionary pressure associated with the increase in the number of proteins that required chaperoning as the fungi increased in complexity.

The duplication and differentiation of genes, giving rise to multigene families, and genomic rearrangements, which allowed for the acquisition of new domains and regulatory sequences, has been a characteristic feature in the evolution of eukaryotic genomes and it is likely to have been a major factor in the evolution of the fungi [112-116]. There is some evidence that gene duplication has occurred during the evolution of the fungal PPIase repertoires. Some of the PPIases in this comparison are apparent paralogues of another PPIase in their respective repertoire (Table 16 [See Additional File 1]): *R. oryzae *(RoCyp1-RoCyp2, RoCyp5-RoCyp6, & RoFKBP2-RoFKBP3), *C. neoformans *(CnCyp3-CnCyp6), *Sz. pombe *(SpFKBP39-SpFKBP39a), *C. albicans *(CaFKBP2-CaFKBP3), *A. fumigatus *(AfFKBP1-AfFKBP2), and in all the Saccharomycota fungi (Group I-Group J). Interestingly, *A. nidulans *has a group of three FKBPs that all appear to be paralogues (AnFKBP1-AnFKBP2-AnFKBP3), suggesting that their progenitor gene may have undergone multiple duplications during the evolution of *A. nidulans*. *A. fumigatus *has a pair of FKBPs that are apparent orthologues of two of these genes (Table 16 [See Additional File 1]; AnFKBP1-AfFKBP1 & AnFKBP3-AfFKBP2), indicating that the evolution of the third paralogue (AnFKBP2) occurred after the divergence of *A. nidulans *from *A. fumigatus*.

There is strong evidence that the genome of the common ancestor of *S. cerevisiae*, *C. glabrata*, and the *Saccharomyces sensu stricto *yeast, underwent whole genome duplication (WGD) after its divergence from the ancestors of the other fungal lineages [117-121]. Whilst a vast majority of the duplicated genome was lost, some duplicate gene copies were retained and subsequently underwent differentiation to form distinct members of the evolving multigene families. This gene retention varied from cell to cell and this is likely to have been a major contributory factor in the divergent evolution of the different yeast that evolved from this ancestral cell. The WGD event appears to have had an apparent role in the evolution of the cyclophilin and FKBP repertoires of *S. cerevisiae *and *C. glabrata*. There are three pairs of PPIase genes present within different duplicated blocks identified in the genome of *S. cerevisiae *[117,122]. Two pairs are cyclophilins (*ScCpr2*-*ScCpr5*, block 14; *ScCpr4*-*ScCpr8*, block 11) and one pair are FKBPs (*ScFpr3*-*ScFpr4*, block 44). Both of the cyclophilin duplications are not present within the genome of *C. glabrata *or any other fungi in this comparison (Table 16A [See Additional File 1]), however there are orthologues of both ScFpr3 and ScFpr4 in the *C. glabrata *repertoire (Table 16B [See Additional File 1]). This would suggest that the functions of these duplicate FKBP genes are important in both *S. cerevisiae *and *C. glabrata*, but the functions of the duplicate gene in both of the *S. cerevisiae *cyclophilin pairs are not required in *C. glabrata*. *K. lactis *is the most recent common ancestor to *S. cerevisiae *and *C. glabrata *in this comparison (Figure 2), and its repertoire contains only one of each pair of duplicate PPIases found in *S. cerevisiae *and *C. glabrata*, supporting their evolution during the post-WGD evolution of these latter fungi.

Gene deletion is also likely to have played role in the evolution of discrete fungi, as exemplified by the PPIase repertoire differences of *C. glabrata *and *S. cerevisiae *after their divergent post-WGD evolution (Table 16 [See Additional File 1]). The differences between the different fungal PPIase repertoires in Table 16 [See Additional File 1] include both additional and absent PPIases between both the different lineages and individual fungi. In many cases, a PPIase orthology group is present in many of the fungi, but it is absent in a particular lineage or in some cases members of a particular lineage. Whilst the presence of some individual PPIases and the evolution of some groups, such as cyclophilin Groups H & J, and FKBP Groups F & I, can be explained easiest by gene duplication, in many others gene deletion appears more likely, such as: cyclophilin Groups A, C, D, K, L, M, & N, and FKBP Groups C & E.

Domain duplication and shuffling by recombination are probably the most important forces driving protein evolution [123], with the majority of multi-domain proteins likely to have evolved by the stepwise insertions of single domains [124]. The dendrogram for the fungal cyclophilins (Figure 1A) and FKBPs (Figure 1B) show evidence that this has probably occurred during the evolution of the fungal PPIase repertoires. There are instances in the cyclophilin dendrogram (Figure 1A) where a multi-domain cyclophilin group appears to have evolved from a uni-domain group, such as: Group N – Group A, Groups K, L & M – Group C, and Groups I & J – Group D. In the FKBP dendrograms (Figure 1B), RoFKBP5 is found on a branch with the members of Group B, a group closely related to Group A with which it shares the larger branch (Figure 1B). It appears likely that the Group B members have evolved by gene duplication given their high sequence homology to their respective Group A member (data not shown). It is therefore possible that RoFKBP5 evolved from a gene duplication event of its Group A member and the subsequent acquisition of its TPR domain that resulted in its divergence from the other Group B members. Interestingly, it has been reported that in most cases domain order is conserved because recombination between the domains usually only occurrs once during the course of evolution [125], and in many protein family combinations, the preservation of the N to C-terminal orientation of the combined domain pair is almost absolute, with only a few examples of domain pairs appearing in both orientations [126]. In the fungal cyclophilin repertoires identified here, we have one group with a C- terminal RNA recognition motif (RRM; Group K), and an individual cyclophilin with an N-terminal RRM (RoCyp10). It therefore appears that multiple recombination events have occurred between the RRM and cyclophilin domains, suggesting that this domain combination may have an important role to play in the complex eukaryotic cell.

The evolution of the fungal PPIase repertoires therefore appears to have been complex involving both gene and whole genome duplication, gene deletion, and multiple domain-acquisition events. Based on the pattern of PPIase conservation and their appearance/disappearance (Table 16 [See Additional File 1]) in relation to the evolutionary history of these fungi (Figure 2), we would hypothesize that the ancestral fungus from which these fungi evolved would have had nine cyclophilins (members of Groups A, B, D, G, I, K, L, M, & N), three FKBPs (members of Groups A, C, & E), and just one parvulin (member of Group B). However, the presence of a member of the second parvuilin group (Group A) cannot be discounted due to its presence within both the Pezizomycotina and the higher eukaryotes. This repertoire size is comparable to that of the current fungal repertoires (Table 15), with much of the variation in size likely due to gene duplication and deletion, as discussed above. The future availability of the complete sequences of additional fungal genomes within which to study the size and makeup of their PPIase repertoires will greatly improve our ability to hypothesize as to the repertoire makeup of the ancestral fungal cell.

The level of orthology between the fungal PPIase repertoires and that of *H. sapiens *has been found to vary. Of the 12 human cyclophilins (Table 16A [See Additional File 1]), out of 17 [68], with orthologues present in these fungi (Table 16A [See Additional File 1]), *S. cerevisiae *only has representatives for four out of its repertoire of nine, which is one less than *E. gossypii *(out of 8), and only marginally better than *C. albicans*, *C. glabrata *and *K. lactis*, which all have three (out of 6). Both *Y. lipolytica *and *D. hansenii *have seven (out of 10) in common with *H. sapiens*, the highest of the Saccharmycotina, with both lacking representatives of hCGI-124, hPPIL3, hCyp33, and hCypF, although they do have an unrelated mitochondrial cyclophilin, and the former also lacks an hCyp57 orthologue and the latter an hCyp60 orthologue. *Sz. pombe*, *A. nidulans*, *A. fumigatus*, *U. maydis*, *N. crassa*, *G. zeae*, and *E. cuniculi*, all share their complete cyclophilin repertoires in common with *H. sapiens*, however they do fall short of having orthologues of all the human cyclophilins. The two Pezizomycotina fungi, *G. zeae *and *N. crassa*, are interesting in that they both have a cyclophilin that has both a cytoplasmic and mitochondrial isoforms (NcCyp4 & GzCyp5) that show orthology with hCypA and hCypF respectively, suggesting a shared evolutionary origin of the cytoplasmic and mitochondrial cyclophilins as shown in the dendrogram (Figure 1A). The other two Pezizomycotina fungi, *A. nidulans *and *A. fumigatus*, both have 11 human orthologues out of the possible 12 with both lacking an hCyp33 orthologue, and both possess separate cyclophilins that show orthology to hCypA and hCypF. This number of human orthologues is shared with *R, oryzae*, which also has 11 out of the possible 12 present in its repertoire of 16. In the case of *R. oryzae*, it is lacking a mitochondrial hCypF orthologue, and it appears to not possess a mitochondrial cyclophilin, although it does have two that show a high degree of sequence homology with the Saccharomycotina mitochondrial cyclophilins (RoCyp5 & RoCyp6; data not shown).

*H. sapiens *has only a single FKBP in common with these fungi, out of 13 [68], the orthologues of human FKBP12 (Group A; Table 16B [See Additional File 1]), which are present in all the fungi with the exception of *E. cuniculi*, which has no FKBPs. There are however two FKBPs within the fruit fly, *Drosophila melanogaster*, that are also present within some of the fungi (DmCG14715 & DmFKBP39; Table 16B [See Additional File 1]). These are both present within *U. maydis*, *C. neoformans*, and *R. oryzae*, and they are both absent within *G. zeae*, *E. gossypii*, and *C. albicans*. DmCG14715 is present in all of the other fungi with the exception of *Sz. pombe*, which is the only other fungi to have an orthologue of DmFKBP39. As with the FKBPs, only one human parvulin is present in all of the fungi, the orthologues of human Pin1 (Group B; Table 16C [See Additional File 1]). The second parvilin group that shows orthology to human Par14 (Group A; Table 16C [See Additional File 1]) is present only within the Pezizomycotina fungi.

Based on this orthology of the fungal PPIase repertoires to that of *H. sapiens*, we have shown that whilst the fungi are good models for the study of the cyclophilins and parvulins, they are a poor model for the study of the FKBPs. This supports the hypothesis that the cyclophilins and parvulins have evolved to perform conserved functions within the underlying biological processes of the cell, whilst the FKBPs have a role largely within protein folding that varies between organisms. The repertoires of *A. nidulans*, *A. fumigatus*, and *C. neoformans *exhibit the closest resemblance, missing only an hCyp33 orthologue, which they share with *R. oryzae*, which instead lacks an hCypF orthologue, and *G. zeae*, however the presence of a single gene encoding the hCypA and hCypF within this latter fungi reduces its resemblance.

*Saccharomyces cerevisiae *has proven to be a very powerful model organism that has, and continues to, contribute greatly to our deciphering of the molecular functioning of our cells [reviewed in 127–129]. The study of its PPIase repertoire has proven invaluable in understanding the elusive functions of these proteins and it continues to provide new insights into their diverse cellular roles [reviewed in 130]. However, as we have shown here and previously [68], its PPIase repertoire poorly represents those of the higher eukaryotes in comparison with the repertoires of *A. nidulans*, *A. fumigatus*, and *C. neoformans*. Whilst research on the *S. cerevisiae *PPIase repertoire will continue to aid us in the search to understand the functions of the PPIases in the eukaryotic cell, research within fungi whose repertoires better represent those of *H. sapiens *and the other Metazoa may offer greater insights into the biological processes within which these PPIases function in the more complex Metazoan cells that cannot be investigated in *S. cerevisiae *whose repertoire lacks orthologues of these PPIases. In this respect, *C. neoformans *may represent a better model fungi as its PPIase repertoire exhibits the highest orthology to the human repertoire of the fungi reported here whilst retaining a genome size close to that of *S. cerevisiae *(Table 15), making it more genetically tractable than *A. nidulans*, *A. fumigatus *and *R. oryzae*, whose genomes are up to three times that of *S. cerevisiae *and *C. neoformans*. It is already an established model organism with the functional genomics and proteomics techniques necessary to fully dissect their function Future experimental investigations are required to elucidate whether or not this will prove to be the case.

## Conclusion

Reported here is the identification of 16 fungal PPIase rep ertoires representing multiple fungal taxa. This comparison has shown that the fungal PPIase repertoires do share some orthology; however this orthology is reduced between the different taxa and is also found to be variable within these taxa. This analysis has also shown that the evolutionary pattern of the cyclophilins and FKBPs appear to be different, with the multi-domain cyclophilins appearing throughout the evolution of the single domain cyclophilins, whereas the multi-domain FKBPs have evolved more recently after the evolution of the single domain FKBPs. Based on the data presented here, we would hypothesize that; (i) the evolution of the fungal PPIases is driven, at least in part, by the size of the proteome, (ii) evolutionary pressures differ both between the different PPIase families and the different fungi, and (iii) whilst the cyclophilins and parvulins have evolved to perform conserved functions, the FKBPs have evolved to perform variable roles that appear predominantly within protein folding. However, further experimental investigations are required to confirm these hypotheses. Interestingly, orthologues of the human parvulin hPar14 and hCyp33 have been identified within the genomes of the Pezizomycotina fungi and *R. oryzae*, respectively. These PPIases were thought to be restricted to the Metazoa. Their presence in these fungi suggests an older evolutionary history than was originally thought. A TPR-containing FKBP was also identified within *R. oryzae*, making it the only known fungal FKBP to possess one. Finally, whilst the PPIase repertoire of *S. cerevisiae *has been the subject of much research, it is a poor representative of the repertoire of *H. sapiens *and despite its highly successful use as a model organism for the study of PPIases over the past two decades, very little is still known about the functions of some of its PPIases. Whilst research into its repertoire will continue to give us insights into the function of the PPIases within the eukaryotic cell, the repertoire of *C. neoformans *may offer a better model system with which to further our knowledge of the role of the PPIases within the human cell given the greater orthology it exhibits towards it. This research has also highlighted the need to fully investigate how representative the model organism chosen to investigate the function of a family of proteins is to that of the primary organism, so as to improve the portability of the results.

## Methods

### Selection of fungal genomes

Fungi for this study were selected primarily by the presence of a complete annotated genome sequence within the database maintained by the National Centre for Biotechnology Information (NCBI; [131]). The complete annotated genome sequences of *Sz. pombe *[132], *E. cuniculi *[133], *S. cerevisiae *[134,135], *D. hansenii *[115], *E. gossypii *[136], *K. lactis *[115], *C. albicans *[137], *C. glabrata *[115], *Y. lipolyica *[115]*, A. nidulans *[138], *A. fumigatus *[139], *N. crassa *[140], &*C. neoformans *[141] were selected for this reason. *G. zeae *[142,143] and *U. maydis *[144] were included as their genomes were 90% and 98% complete, respectively, and they are present in an annotated state within the NCBI database. The unannotated genome of *R. oryzae *[145] was included as it is the only example of the *Zygomycota *in the NCBI database. In all three cases, there are no plans to complete the sequencing and annotation of their genomes making their current sequence repository as complete a picture as we are likely to achieve of their repertoires.

### Identification of the PPIase repertoires

The repertoires of *Sz. pombe *and *S. cerevisiae *were as previously described [68]. The identification of putative PPIases in the remaining fungi was performed using both BLASTP (protein vs. protein) and TBLASTN (protein against DNA sequence) searches [146] on all genomes except for that of *R. oryzae*, where only TBLASTN searches were possible. The TBLASTN step is important for detecting additional homologues because genes may remain unannotated in the sequence databases. The members of the three PPIase families were identified using the protein sequences of human cyclophilin A (hCypA; UniProt accession # P05092), human FKBP12 (hFKBP12; P20071) and the human parvulins Pin1 (hPin1; Q13526) and Par14 (hPar14; Q9Y237) as probes in BLASTP and TBLASTN searches of their sequences. Proteins were selected based upon the level of homology, both in regard to actual sequence homology and/or the presence of characteristic motifs, their PPIase catalytic domain exhibited towards that of their probes sequence. As the catalytic domain of the different PPIases families have been found to exhibit good conservation between related organisms [4,5,147], a conservative expected (E) value cut-off of 1 × 10^-10 ^and/or greater or equal to 30% alignment sequence identity [148] was used to identify homologues of the three PPIase families. Two fungal cyclophilin groups that possess a divergent PPIase domain have been previously identified [11]. To confirm that all members of these groups were identified (Table 16 [See Additional File 1]; Groups K & L), their *Sz. pombe *and *S. cerevisiae *members were used as probes in BLASTP and TBLASTN searches of the fungal proteomes and genomes, respectively.

In some cases, it was apparent that the sequence present in the NCBI database was incorrect or incomplete. In these instances, sequences were investigated and where possible corrected prior to further analysis. Sequence corrections obtained by this manual curation were submitted to the Universal Protein Resource (UniProt) consortium for verification [149]. Once verified, the appropriate UniProt reports were ammended to reflect this revised annotation. All protein sequences are available in the UniProt database and their accession numbers are given in Tables 1, 2, 3, 4, 5, 6, 7, 8, 9, 10, 11, 12, 13, 14, and those that were manually re-annotated are marked.

### Protein sequence analysis

The identification of putative domains within the identified PPIases was performed using the NCBI CDD (Conserved Domain Database) database [150,151]. The predicted localisation of the PPIases and the identification of sequence motifs that support this were identified using PSORT [152,153] located on the National Institute for Basic Biology (NIBB) server [154]. The theoretical molecular mass of the predicted proteins were calculated using the calculation tool on the ExPASy server [155]. Pairwise percentage sequence identity and similarity was calculated using the Matrix Global Alignment Tool (MatGAT) version 2.02 [156] using a BLOSUM50 scoring matrix.

### Comparative sequence analysis

Multiple sequence alignments (MSA) were produced using default settings in version 1.83 of the ClustalX program [157]. This program performs a pairwise alignment of the sequences prior to the construction of a dendrogram, which describes the approximate groupings of the sequences by similarity, with the final alignment carried out using this dendrogram as a guide. The dendrograms presented in Figure 1 were constructed from a global MSA of each family of PPIases by the Neighbour Joining (NJ) method with a Poisson correction for distance estimation [158] and 500 bootstrap replicates. The dendrograms were visualised from the files generated by the ClustalX alignment using MEGA version 3.1 [159]. The scales of the different dendrograms are not cross-comparable.

### Identification of orthologues

PPIases were considered to be orthologues if they fulfilled three criteria. Firstly, they should be of approximately the same size and possess the same domain architecture. Secondly, in BLAST searches they should identify each other ahead of all other PPIases within their respective genomes [160]. This is because they should, in theory, share a more recent common ancestor than they do with the other PPIases. Sequence variation, resulting from the distinct divergent evolution of each protein, should therefore be less between two orthologues than with other PPIases. Thirdly, they should have the same intracellular location and function. This latter criterion is however reliant upon prior research which is not applicable to all PPIases. In these cases, as long as the first two criteria were met, then the proteins were deemed to be putative orthologues.

Four methods were employed to identify the orthology between the repertoires using the above criteria. Firstly, the sequences of the identified PPIases were formatted by family as a database file using the BLAST tools package obtained from the ftp-site of NCBI [131,146] and an "all against all" BLASTP method was used to identify homology between proteins. Orthologues were defined in the following manner: (i) they must be reciprocal best hits with an expectation (E)-value less than 1 × 10^-10 ^[160], (ii) they must share at least 40% similarity in amino acid sequence [160], (iii) there must be <20% difference in protein length [160], and (iv) have a bit score above 60, which in almost all cases indicate a biologically relevant relationship when motifs are conserved [161,162]. However, in cases where a protein failed to meet one of these criteria but where membership to a particular orthology group was well supported by the remaining BLAST criteria as well as by the remaining identification methods detailed below, the protein was included as an orthologue of that particular group. Secondly, each orthology group identified by BLAST analysis were subjected to sequence alignment using the ClustalX program version 1.83 [157] to check for conserved domain architecture and conserved sequence motifs [See Additional File 8]. Thirdly, the sequences for all the member proteins of each of the three different PPIase families (cyclophilins, FKBPs & parvulins) from all of the compared fungi were subjected to global sequence comparison by family using the ClustalX program version 1.83 [157] for the purpose of creating a dendrogram. This analysis creates a putative model for how the individual sub-groups of each PPIase family may have diverged from one another based on relationships between their individual sequences, which allow us to infer a putative model for their evolution in the fungi compared here. As each orthology group should share a more recent common ancestor with themselves than with the other PPIases they should cluster together in the MSA [148] and therefore within the dendrogram, ideally as an individual branch with a distinct common ancestor. Fourthly, literature analysis looking for prior publications on the individual PPIases was performed which in some cases has allowed putative function(s) to be assigned to orthology groups.

## Abbreviations

Peptidyl-prolyl *cis*/*trans *isomerase (PPIase); cyclophilin (Cyp); FK506 binding-protein (FKBP); parvulin (Par, Pin or Ess); cyclosporin A (CsA); *R. oryzae *(Ro); *Sz. pombe *(Sp); *E. cuniculi *(Ec); *S. cerevisiae *(Sc); *D. hansenii *(Dh); *E. gossypii *(Eg); *K. lactis *(Kl); *C. albicans *(Ca); *C. glabrata *(Cg); *Y. lipolyica *(Yl);*A. nidulans *(An); *A. fumigatus *(Af); *N. crassa *(Nc); *G. zeae *(Gz); *U. maydis *(Um); *C. neoformans *(Cn); *H. sapiens *(h); endoplasmic reticulum (ER); nuclear localisation sequence (NLS); RNA Recognition Motif (RRM); tetratrico peptide repeat (TPR).

## Competing interests

The author(s) declare that they have no competing interests.

## Supplementary Material

Additional File 1 Table 16. The orthology between the three PPIase families that make up the fungal repertoires. Table depicting the orthology between the repertoires of these sixteen fungi (Tables 1, 2, 3, 4, 5, 6, 7, 8, 9, 10, 11, 12, 13, 14 & Ref. 68) as determined by BLASTP analysis. Click here for file

Additional File 2 Pairwise Expected (E)-values and bit scores of the cyclophilins. Pairwise expected (E)-values and bit scores for all members of each of the individual cyclophilin groups, and a global comparison of all the cyclophilins identified here. Click here for file

Additional File 3 Pairwise percentage sequence identity and similarity of the cyclophilins. Pairwise percentage sequence identity and similarity for all members of each of the individual cyclophilin groups, and a global comparison of all the cyclophilins identified in this report. Click here for file

Additional File 4 Pairwise expected (E)-values and bit scores of the FKBPs. Pairwise expected (E)-values and bit scores for all members of each of the individual FKBP groups, and a global comparison of all the FKBPs identified in this report. Click here for file

Additional File 5 Pairwise percentage sequence identity and similarity of the FKBPs. Pairwise percentage sequence identity and similarity for all members of each of the individual FKBP groups and a global comparison of all the FKBPs identified in this report. Click here for file

Additional File 6 Pairwise expected (E)-values and bit scores of the parvulins. Pairwise expected (E)-values and bit scores for all members of each of the individual parvulin groups, and a global comparison of all the parvulins identified in this report. Click here for file

Additional File 7 Pairwise percentage sequence identity and similarity of the parvulins. Pairwise percentage sequence identity and similarity for all members of each of the individual parvulin groups, and a global comparison of all the parvulins identified in this report. Click here for file

Additional File 8 Multiple sequence alignments of the different cyclophilin, FKBP and parvulin groups. Multiple sequence alignments of the different cyclophilin, FKBP and parvulin groups that were identified by BLAST analysis of their sequences. All alignments were constructed with ClustalX. Click here for file

## Figures and Tables

**Table 1:** PPIase repertoire of *Aspergillus nidulans*.

	**PPIase**	**Uniprot Acc. #**	**kDa**	**Signal Seq.**	**Predicted Localisation**	**Domain Architecture**
**Cyclophilins**	Cyp1	Q5ASX5	17.8	~	Cytoplasmic	PPIase only
	Cyp2	Q5ASQ0	17.8	~	Cytoplasmic	PPIase only
	Cyp3	Q5B6L6	18.7	~	Cytoplasmic	PPIase only
	Cyp4	Q5AQL0*	20.0	~	Cytoplasmic	PPIase only
	Cyp5	Q5BAH7*	22.5	~	Cytoplasmic	PPIase only
	Cyp6	Q5B4R3	23.4	N-term.	ER	PPIase only
	Cyp7	Q5B4E7	41.1	~	Cytoplasmic	N-term. PPIase only
	Cyp8	Q5ARI5	52.9	~	Nuclear	N-term. PPIase, Central RRM
	Cyp9	Q5AXT6	64.1	~	Cytoplasmic	N-term. U-Box, C-term. PPIase
	Cyp10	Q5BGF0	69.5	~	Cytoplasmic	N-term. WD-40 (1x), C-term. PPIase
	Cwc27	Q5AUG9	61.7	~	Nuclear	N-term. PPIase, C-term. Positively charged

**FKBPs**	FKBP1	Q5B782	11.9	~	Cytoplasmic	FKBP (1x) only
	FKBP2	Q5ASU9	12.8	~	Cytoplasmic	FKBP (1x) only
	FKBP3	Q5AXD5*	12.9	~	Cytoplasmic	FKBP (1x) only
	FKBP4	Q5ATN7	14.6	N-term.	ER	FKBP (1x) only
	FKBP5	Q5B6C2*	52.8	~	Cytoplasmic	N-term. Negatively charged, C-term. FKBP (1x)

**Parvulins**	Par1	Q5B5W1	13.9	~	Nuclear	Rotamase only
	Pin1	Q5AZY5	24.4	~	Cytoplasmic	N-term. WW domain, C-term. Rotamase

* manually re-annotated.

**Table 2:** PPIase repertoire of *Aspergillus fumigatus*.

	**PPIase**	**Uniprot Acc. #**	**kDa**	**Signal Seq.**	**Predicted Localisation**	**Domain Architecture**
**Cyclophilins**	Cyp1	Q4WCR3	17.7	~	Cytoplasmic	PPIase only
	Cyp2	Q4WWX5	17.7	~	Cytoplasmic	PPIase only
	Cyp3	Q4WCM6*	19.9	~	Cytoplasmic	PPIase only
	Cyp4	Q4WHY9	22.3	~	Cytoplasmic	PPIase only
	Cyp5	Q4WMB6*	22.5	~	Cytoplasmic	PPIase only
	Cyp6	Q4WP12	23.0	N-term.	ER	PPIase only
	Cyp7	Q4WIF3	41.8	~	Cytoplasmic	N-term. PPIase only
	Cyp8	Q4WAQ9	52.9	~	Nuclear	N-term. PPIase, Central RRM
	Cyp9	Q4WVU5*	63.3	~	Cytoplasmic	N-term. U-Box, C-term. PPIase
	Cyp10	Q4WKP5	69.9	~	Cytoplasmic	N-term. WD-40 (1x), C-term. PPIase
	Cwc27	Q4WE62	62.0	~	Nuclear	N-term. PPIase, C-term. Positively charged

**FKBPs**	FKBP1	Q4WLV6	12.1	~	Cytoplasmic	FKBP (1x) only
	FKBP2	Q4W9R2	13.2	~	Cytoplasmic	FKBP (1x) only
	FKBP3	Q4WHX4	14.6	N-term.	ER	FKBP (1x) only
	FKBP4	Q4WMV5*	49.7	~	Cytoplasmic	N-term. Negatively charged, C-term. FKBP (1x)

**Parvulins**	Par1	Q4WJM6	14.0	~	Nuclear	Rotamase only
	Pin1	Q4X1W4	21.3	~	Nuclear	N-term. WW domain, C-term. Rotamase

* manually re-annotated.

**Table 3:** PPIase repertoire of *Candida albicans*.

	**PPIase**	**Uniprot Acc. #**	**kDa**	**Signal Seq.**	**Predicted Localisation**	**Domain Architecture**
**Cyclophilins**	Cyp1	Q5AH66	17.6	~	Cytoplasmic	PPIase only
	Cyp2	Q5ALM6	21.0	~	Mitochondrial	PPIase only
	Cyp3	Q5A2Z0	25.4	~	ER	PPIase only
	Cyp4	Q59YT3	28.8	N-term.	ER	PPIase only
	Cyp5	Q5ACI8*	40.7	~	Cytoplasmic	N-term. PPIase, C-term. TPR (x3) motifs
	Cyp6	Q5A9Y5	42.6	~	Nuclear	N-term. PPIase only

**FKBPs**	FKBP1	Q59LZ3	13.3	~	Cytoplasmic	FKBP (1x) only
	FKBP2	Q59VR3	47.7	~	Nuclear	N-term. Negatively charged, C-term. FKBP (1x)
	FKBP3	Q59VV9	47.9	~	Nuclear	N-term. Negatively charged, C-term. FKBP (1x)

**Parvulin**	Ess1	Q59KZ2	19.9	~	Nuclear	N-term. WW domain, C-term. Rotamase

* manually re-annotated.

**Table 4:** PPIase repertoire of *Candida glabrata*.

	**PPIase**	**Uniprot Acc. #**	**kDa**	**Signal Seq.**	**Predicted Localisation**	**Domain Architecture**
**Cyclophilins**	Cyp1	Q6FVK5	17.4	~	Cytoplasmic	PPIase only
	Cyp2	Q6FS12	20.2	~	Mitochondrial	PPIase only
	Cyp3	Q6FSC9	22.6	N-term.	ER	PPIase only
	Cyp4	Q6FPI7	36.5	N-term.	ER	PPIase only
	Cyp5	Q6FNU6	41.6	~	Cytoplasmic	N-term. PPIase only
	Cyp6	Q6FJ99	44.2	~	Cytoplasmic	N-term. PPIase, C-term. TPR (x3) motifs

**FKBPs**	FKBP1	Q6FMA3	12.2	~	Cytoplasmic	FKBP (1x) only
	FKBP2	Q6FSC1	14.8	~	Cytoplasmic	FKBP (1x) only
	FKBP3	Q6FK71	44.8	~	Cytoplasmic	N-term. Negatively charged, C-term. FKBP (1x)
	FKBP4	Q6FKH7	49.7	~	Nuclear	N-term. Negatively charged, C-term. FKBP (1x)

**Parvulin**	Pin1	Q6FXP9	20.1	~	Nuclear	N-term. WW domain, C-term. Rotamase

**Table 5:** PPIase repertoire of *Cryptococcus neoformans*.

	**PPIase**	**Uniprot Acc. #**	**kDa**	**Signal Seq.**	**Predicted Localisation**	**Domain Architecture**
**Cyclophilins**	Cyp1	Q5KHA8*	18.2	~	Cytoplasmic	
	Cyp2	Q5KKX7	18.7	~	Cytoplasmic	PPIase only
	Cyp3	Q5KMM0	19.5	~	Cytoplasmic	PPIase only
	Cyp4	Q5KA96	19.6	~	Cytoplasmic	PPIase only
	Cyp5	Q5KML4	21.1	~	Mitochondrial	PPIase only
	Cyp6	Q5KHA0	21.6	~	Cytoplasmic	PPIase only
	Cyp7	Q5KEB7	25.0	N-term.	ER	PPIase only
	Cyp8	Q5KFV5	40.9	~	Cytoplasmic	N-term. PPIase only
	Cyp9	Q5KNY5	57.9	~	Nuclear	N-term. PPIase, Central RRM
	Cyp10	Q5KA10	60.4	~	Nuclear	N-term. PPIase, C-term. Positively charged.
	Cyp11	Q5KAW8	63.6	~	Cytoplasmic	N-term. U-Box, C-term. PPIase
	Cyp12	Q5KMG4	72.9	~	Cytoplasmic	N-term. WD-40 (1x), C-term. PPIase
	Cwc27	Q5KAB3	55.2	~	Nuclear	N-term. PPIase, C-term. Positively charged

**FKBPs**	FKBP1	Q5KMG3*	11.6	~	Cytoplasmic	FKBP (1x) only
	FKBP2	Q5KGT9	15.7	N-term.	ER	FKBP (1x) only
	FKBP3	Q5KIJ5	44.4	N-term.	ER	N-term. Negatively charged, C-term. FKBP (1x)

**Parvulin**	Pin1	Q8NJN5	19.5	~	Nuclear	N-term. WW domain, C-term. Rotamase

* manually re-annotated.

**Table 6:** PPIase repertoire of *Debaryomyces hansenii*.

	**PPIase**	**Uniprot Acc. #**	**kDa**	**Signal Seq.**	**Predicted Localisation**	**Domain Architecture**
**Cyclophilins**	Cyp1	Q6BP00	17.6	~	Cytoplasmic	PPIase only
	Cyp2	Q6BYF7	18.8	~	Nuclear	PPIase only
	Cyp3	Q6BWY3	20.4	~	Mitochondrial	PPIase only
	Cyp4	Q6BIH6	23.0	~	ER	PPIase only
	Cyp5	Q6BUC1	32.7	N-term.	ER	PPIase only
	Cyp6	Q6BPQ5	37.7	~	Cytoplasmic	N-term. PPIase, C-term. RRM
	Cyp7	Q6BXZ7	40.7	~	Cytoplasmic	N-term. PPIase, C-term. TPR (x3) motifs
	Cyp8	Q6BY72	43.9	~	Cytoplasmic	N-term. PPIase, C-term. Positively charged
	Cyp9	Q6BSZ1	71.9	~	Nuclear	N-term. WD-40 (1x), C-term. PPIase
	Cwc27	Q6BWH6	37.3	~	Nuclear	N-term. PPIase, C-term. Positively charged

**FKBPs**	FKBP1	Q6BX45	12.0	~	Cytoplasmic	FKBP (1x) only
	FKBP2	Q6BP84*	14.6	N-term.	ER	FKBP (1x) only
	FKBP3	Q6BSE7	48.8	~	Nuclear	N-term. Positively charged, C-term. FKBP (1x)

**Parvulin**	Pin1	Q6BRV8	19.7	~	Nuclear	N-term. WW domain, C-term. Rotamase

* manually re-annotated.

**Table 7:** PPIase repertoire of *Encephalitozoon cuniculi*.

	**PPIase**	**Uniprot Acc. #**	**kDa**	**Signal Seq.**	**Predicted Localisation**	**Domain Architecture**
**Cyclophilin**	Cyp1	Q8SRE1	19.0	~	Cytoplasmic	PPIase only
	Cyp2	Q8SQZ8	22.6	N-term.	ER	PPIase only

**Parvulin**	Pin1	Q8SRS5	17.2	~	Nuclear	C-term. Rotamase only

**Table 8:** PPIase repertoire of *Eremothecium gossypii*.

	**PPIase**	**Uniprot Acc. #**	**kDa**	**Signal Seq.**	**Predicted Localisation**	**Domain Architecture**
**Cyclophilins**	Cyp1	Q750W6	17.4	~	Cytoplasmic	PPIase only
	Cyp2	Q756U6	20.3	~	Mitochondrial	PPIase only
	Cyp3	Q75EY4	21.8	N-term.	ER	PPIase only
	Cyp4	Q75EN4	34.3	~	ER	PPIase only
	Cyp5	Q752C6	34.3	~	Cytoplasmic	N-term. PPIase, C-term. RRM
	Cyp6	Q75A33	40.0	~	Cytoplasmic	N-term. PPIase only
	Cyp7	Q757S4	42.8	~	Cytoplasmic	N-term. PPIase, C-term. TPR (x3) motifs
	Cwc27	Q75A74	32.5	~	Nuclear	N-term. PPIase, C-term. Positively charged

**FKBPs**	FKBP1	Q754K8	12.3	~	Cytoplasmic	FKBP (1x) only
	FKBP2	Q756V1	46.5	~	Nuclear	N-term. Negatively charged, C-term. FKBP (1x)

**Parvulin**	Pin1	Q75CN9	18.4	~	Cytoplasmic	N-term. WW domain, C-term. Rotamase

**Table 9:** PPIase repertoire of *Gibberella zeae*.

	**PPIase**	**Uniprot Acc. #**	**kDa**	**Signal Seq.**	**Predicted Localisation**	**Domain Architecture**
**Cyclophilins**	Cyp1	Q4I1Y1	17.7	~	Cytoplasmic	PPIase only
	Cyp2	Q4IPB8*	18.0	~	Cytoplasmic	PPIase only
	Cyp3	Q4IPH4	20.0	~	Cytoplasmic	PPIase only
	Cyp4	Q4I5R9	22.6	N-term.	ER	PPIase only
	Cyp5	Q4IPT1	24.4	~	Mitochondrial	PPIase only
	Cyp6	Q4HXF6	40.3	~	Cytoplasmic	N-term. PPIase only
	Cyp7	Q4IBK5	64.8	~	Cytoplasmic	N-term. U-Box, C-term. PPIase
	Cyp8	Q4IE79	68.4	~	Nuclear	N-term. PPIase, Central RRM
	Cyp9	Q4IQM8	69.9	~	Cytoplasmic	N-term. WD-40 (1x), C-term. PPIase
	Cwc27	Q4IPB3	60.9	~	Nuclear	N-term. PPIase, C-term. Positively charged

**FKBPs**	FKBP1	Q4HZB8*	11.9	~	Cytoplasmic	FKBP (1x) only
	FKBP2	Q4IN00	20.4	~	ER	Central FKBP (1x) only
	FKBP3	Q4INZ9	53.3	~	Cytoplasmic	N-term. Negatively charged, C-term. FKBP (1x)

**Parvulins**	Par1	Q4I665*	14.3	~	Cytoplasmic	N-term. Positively charged, N-term Rotamase
	Pin1	Q4I8C6	19.7	~	Nuclear	N-term. WW domain, C-term. Rotamase

* manually re-annotated

**Table 10:** PPIase repertoire of *Kluyveromyces lactis*.

	**PPIase**	**Uniprot Acc. #**	**kDa**	**Signal Seq.**	**Predicted Localisation**	**Domain Architecture**
**Cyclophilins**	Cyp1	Q6CQJ3	17.3	~	Cytoplasmic	PPIase only
	Cyp2	Q6CWF3	21.1	~	Mitochondrial	PPIase only
	Cyp3	Q6CTT5	22.2	N-term.	ER	PPIase only
	Cyp4	Q6CU04	34.5	N-term.	ER	PPIase only
	Cyp5	Q6CL78	41.3	~	Cytoplasmic	N-term. PPIase, C-term. TPR (x3) motifs
	Cyp6	Q6CM61	44.6	~	Cytoplasmic	N-term. PPIase, C-term. TPR (x3) motifs

**FKBPs**	FKBP1	Q6CX30	12.2	~	Cytoplasmic	FKBP (1x) only
	FKBP2	Q6CUZ8	15.3	N-term.	ER	FKBP (1x) only
	FKBP3	Q6CWE8	47.3	~	Nuclear	N-term. Negatively charged, C-term. FKBP (1x)

**Parvulin**	Pin1	Q6CMZ3	18.39	~	Nuclear	N-term. WW domain, C-term. Rotamase

**Table 11:** PPIase repertoire of *Neurospora crassa*.

	**PPIase**	**Uniprot Acc. #**	**kDa**	**Signal Seq.**	**Predicted Localisation**	**Domain Architecture**
**Cyclophilins**	Cyp1	Q7SF72*	17.9	~	Cytoplasmic	PPIase only
	Cyp2	Q7SG06	19.7	~	Cytoplasmic	PPIase only
	Cyp3	Q7S7Z6*	22.6	N-term.	ER	PPIase only
	Cyp4	P10255	24.1	~	Mitochondrial	PPIase only
	Cyp5	Q9P3X9	40.6	~	Cytoplasmic	N-term. PPIase, C-term. TPR (x3) motifs
	Cyp6	Q7RXA6	65.8	~	Cytoplasmic	N-term. U-Box, C-term. PPIase
	Cyp7	Q7SCP8	70.6	~	Cytoplasmic	N-term. WD-40 (1x), C-term. PPIase
	Cyp8	Q7RZ18	90.8	~	Nuclear	N-term. PPIase, Central RRM
	Cwc27	Q7SBX8	60.3	~	Nuclear	N-term. PPIase, C-term. Positively charged

**FKBPs**	FKBP1	Q6M981	11.8	~	Cytoplasmic	FKBP (1x) only
	FKBP2	P20080	13.0	~	Cytoplasmic	FKBP (1x) only
	FKBP3	O60046	22.9	N-term.	ER	FKBP (1x) only
	FKBP4	Q7SCN0	50.8	~	Cytoplasmic	N-term. Negatively charged, C-term. FKBP (1x)

**Parvulins**	Par1	Q7RYY4	13.7	~	Nuclear	Rotamase only
	Pin1	Q7RVY7	21.2	~	Nuclear	N-term. WW domain, C-term. Rotamase

* manually re-annotated.

**Table 12:** PPIase repertoire of *Rhizopus oryzae*.

	**PPIase**	**Uniprot Acc. #**	**kDa**	**Signal Seq.**	**Predicted Localisation**	**Domain Architecture**
**Cyclophilins**	Cyp1	P0C1H8*	17.6	~	Nuclear	PPIase only
	Cyp2	P0C1H7	17.8	~	Cytoplasmic	PPIase only
	Cyp3	P0C1I4*	18.4	~	Cytoplasmic	PPIase only
	Cyp4	P0C1I5	18.9	~	Cytoplasmic	PPIase only
	Cyp5	P0C1I7	19.0	~	Cytoplasmic	PPIase only
	Cyp6	P0C1I8	19.2	~	Cytoplasmic	PPIase only
	Cyp7	P0C1I3*	19.7	~	Cytoplasmic	PPIase only
	Cyp8	P0C1H9	22.7	N-term.	ER	PPIase only
	Cyp9	P0C1I0	22.9	N-term.	ER	PPIase only
	Cyp10	P0C1I2	34.7	~	Cytoplasmic	N-term. RRM, C-term. PPIase
	Cyp11	P0C1I9*	39.1	~	Nuclear	N-term. PPIase, C-term. Positively charged
	Cyp12	P0C1I1	40.4	~	Cytoplasmic	N-term. PPIase, C-term. TPR (x3) motifs
	Cyp13	P0C1I6	51.7	~	Nuclear	N-term. PPIase, Central RRM
	Cyp14	P0C1J1*	61.7	~	Nuclear	N-term. U-Box, C-term. PPIase
	Cyp15	P0C1J0	71.3	~	Cytoplasmic	N-term. WD40 (x3), C-term. PPIase
	Cwc27	P0C1J2	61.0	~	Nuclear	N-term. PPIase, C-term. Positively charged

**FKBPs**	FKBP1	P0C1J3	11.7	~	Cytoplasmic	FKBP (1x) only
	FKBP2	P0C1J4	18.6	N-term.	ER	FKBP (1x) only
	FKBP3	P0C1J5	22.6	N-term.	ER	FKBP (1x) only
	FKBP4	P0C1J6	42.9	~	Nuclear	N-term. Negatively charged, C-term. FKBP (1x)
	FKBP5	P0C1J7	44.0	~	Cytoplasmic	N-term. FKBP(1x), Central TPR(3x)

**Parvulin**	Pin1	P0C1J8	17.4	~	Nuclear	N-term. WW domain, C-term. Rotamase

* manually re-annotated.

**Table 13:** PPIase repertoire of *Ustilago maydis*.

	**PPIase**	**Uniprot Acc. #**	**kDa**	**Signal Seq.**	**Predicted Localisation**	**Domain Architecture**
**Cyclophilins**	Cyp1	Q4P837	17.4	~	Cytoplasmic	PPIase only
	Cyp2	Q4PCH8	18.0	~	Cytoplasmic	PPIase only
	Cyp3	Q4PFU5	22.9	N-term.	ER	PPIase only
	Cyp4	Q4P6X6*	17.2	~	Cytoplasmic	Partial PPIase only
	Cyp5	Q4P0V4	43.6	~	Nuclear	N-term. PPIase only
	Cyp6	Q4P8S3*	63.7	~	Nuclear	N-term. PPIase, Central RRM.
	Cyp7	Q4P555	64.5	~	Nuclear	N-term. U-Box, C-term. PPIase
	Cyp8	Q4P6F3	93.5	~	Nuclear	N-term. Positively charged, C-term. PPIase.
	Cwc27	Q4P7H2	54.0	~	Nuclear	N-term. PPIase, C-term. Positively charged

**FKBPs**	FKBP1	Q4P608	15.2	~	ER	FKBP (1x) only
	FKBP2	Q4P790	27.0	~	Nuclear	N-term. FKBP (1x) only
	FKBP3	Q4PIN7	41.1	~	Nuclear	N-term. Negatively charged, C-term. FKBP (1x)

**Parvulin**	Pin1	Q4P1L0	19.4	~	Nuclear	C-term. Rotamase only

* manually re-annotated.

N.B.: The N-terminal sequence of UmCyp4 is truncated [See Additional File 8] due to the absence of genomic DNA sequence 5' of the current start of the protein sequence in the available genomic sequence database (GenBank ID: AACP01000148).

**Table 14:** PPIase repertoire of *Yarrowia lipolytica*.

	**PPIase**	**Uniprot Acc. #**	**kDa**	**Signal Seq.**	**Predicted Localisation**	**Domain Architecture**
**Cyclophilins**	Cyp1	Q6CCD9	17.7	~	Cytoplasmic	PPIase only
	Cyp2	Q6CCS1	19.1	~	Cytoplasmic	PPIase only
	Cyp3	Q6CCE8	19.3	~	Mitochondrial	PPIase only
	Cyp4	Q6C4W6	24.9	N-term.	ER	PPIase only
	Cyp5	Q6H9N9	39.4	~	Nuclear	N-term. PPIase, C-term. Positively charged
	Cyp6	Q6CBP4	40.1	~	Cytoplasmic	N-term. PPIase, C-term. TPR (x3) motifs
	Cyp7	Q6CGQ3	42.6	~	Cytoplasmic	N-term. PPIase, C-term. TPR (x3) motifs
	Cyp8	Q6C7K2	52.8	~	Nuclear	C-term. PPIase only
	Cyp9	Q6CBT5	71.1	~	Cytoplasmic	N-term. WD-40 (1x), C-term. PPIase
	Cwc27	Q6CGK4	39.7	~	Nuclear	N-term. PPIase, C-term. Positively charged

**FKBPs**	FKBP1	Q6CF41	11.6	~	Cytoplasmic	FKBP (1x) only
	FKBP2	Q6CGG3	15.6	N-term.	ER	FKBP (1x) only
	FKBP3	Q6C4C9	45.2	~	Cytoplasmic	N-term. Negatively charged, C-term. FKBP (1x)

**Parvulin**	Pin1	Q6CEL8	20.2	~	Nuclear	N-term. WW domain, C-term. Rotamase

**Table 15:** The numbers of the three different PPIase families present in each of the fungal repertoires.

**Organism**	**Genes**	**Cyclophilins**		**FBKPs**		**Parvulins**		**Total**		**Common Taxa**
			
		**Number**	**/1000 genes**	**Number**	**/1000 genes**	**Number**	**/1000 genes**	**Number**	**/1000 genes**	
*Rhizopus oryzae*	17,467^a^	16	0.92	5	0.29	1	0.06	22	1.26	**Zygomycetes**

*Encephalitozoon cuniculi*	1,997^b^	2	1.00	0	0	1	0.50	3	1.50	**Apansporoblastina**

*Schizosaccharomyces pombe*	4,824^c^	9	1.87	3	0.62	1	0.21	13	2.69	**Schizosaccharomycetes**

*Yarrowia lipolytica*	6,703^d^	10	1.49	3	0.45	1	0.15	14	2.09	**Saccharomycotina**
*Candida albicans*	6,419^e^	6	0.93	3	0.47	1	0.16	10	1.56	
*Debaryomyces hansenii*	6,906^d^	10	1.45	3	0.43	1	0.14	14	2.03	
*Eremothecium gossypii*	4,718^f^	8	1.70	2	0.42	1	0.21	11	2.33	
*Kluyveromyces lactis*	5,329^d^	6	1.13	3	0.56	1	0.19	10	1.88	
*Candida glabrata*	5,283^d^	6	1.14	4	0.76	1	0.19	11	2.08	
*Saccharomyces cerevisiae*	5,885^g^	8	1.36	4	0.68	1	0.17	13	2.21	

*Aspergillus nidulans*	9,541^h^	11	1.15	5	0.52	2	0.21	18	1.89	**Pezizomycotina**
*Aspergillus fumigatus*	9,926^i^	11	1.11	4	0.40	2	0.20	17	1.71	
*Neurospora crassa*	10,082^j^	9	0.89	4	0.40	2	0.20	15	1.49	

*Gibberella zeae*	14,086^k^	10	0.71	3	0.21	2	0.14	15	1.06	

*Ustilago maydis*	6,522^l^	9	1.38	3	0.46	1	0.15	13	1.99	**Ustilaginomycetes**

*Cryptococcus neoformans*	6,572^m^	13	1.98	3	0.46	1	0.15	17	2.59	**Hymenomycetes**

"a" = [145], "b" = [133], "c" = [132], "d" = [115], "e" = [137], "f" = [136], "g" = [134], "h" = [138], "i" = [139], "j" = [140], "k" = [142, 143], "l" = [144], "m" = [141]

## References

[B1] Adams B, Musiyenko A, Kumar R, Barik S (2005). A Novel Class of Dual-family Immunophilins. J Biol Chem.

[B2] Galat A (1993). Peptidyl proline cis-trans-isomerases: immunophilins. Eur J Biochem.

[B3] Galat A, Metcalfe SM (1995). Peptidyl proline cis/trans isomerases. Progress Biophys Mol Biol.

[B4] Galat A (1999). Variations of sequences and amino acid compositions of proteins that sustain their biological functions: An analysis of the cyclophilin family of proteins. Arch Biochem Biophys.

[B5] Rulten S, Thorpe J, Kay J (1999). Identification of eukaryotic parvulin homologues: a new subfamily of peptidylprolyl cis-trans isomerases. Biochem Biophys Res Comm.

[B6] Maruyama T, Furuani M (2000). Archael Peptidyl-Prolyl cis/trans Isomerases (PPIases). Front Biosci.

[B7] Ivery MTG (2000). Immunophillins: Switched on Protein Binding Domains. Med Res Rev.

[B8] He Z, Li L, Luan S (2004). Immunophilins and Parvulins. Superfamily of Peptidyl Prolyl Isomerases in Arabidopsis.. Plant Physiol.

[B9] Kumar A, Agarwal S, Heyman JA, Matson S, Heidtman M, Piccirillo S, Umansky L, Drawid A, Jansen R, Liu Y, Cheung KH, Miller P, Gerstein M, Roeder GS, Snyder M (2002). Subcellular localization of the yeast proteome. Genes Dev.

[B10] Huh WK, Falvo JV, Gerke LC, Carroll AS, Howson RW, Weissman JS, O'Shea EK (2003). Global analysis of protein localization in budding yeast.. Nature.

[B11] Pemberton TJ, Kay JE (2005). The Cyclophilin repertoire of the fission yeast Schizosaccharomyces pombe.. Yeast.

[B12] Justice SS, Hunstad DA, Harper JR, Duguay AR, Pinkner JS, Bann J, Frieden C, Silhavy TJ, Hultgren SJ (2005). Periplasmic Peptidyl Prolyl cis-trans Isomerases Are Not Essential for Viability, but SurA Is Required for Pilus Biogenesis in Escherichia coli. J Bacteriol.

[B13] Gothel SF, Scholz C, Schmid FX, Marahiel MA (1998). Cyclophilin and trigger factor from Bacillus subtilis catalyze in vitro protein folding and are necessary for viability under starvation conditions.. Biochemistry.

[B14] Dolinski K, Muir S, Cardenas M, Heitman J (1997). All cyclophilins and FK506 binding proteins are, individually and collectively, dispensable for viability in Saccharomyces cerevisiae. Proc Nat Acad Sci USA.

[B15] Hemenway C, Heitman J (1993). Proline Isomerases in Microorganisms and small Eukaryotes.. Ann NY Acad Sci.

[B16] Hanes SD, Shank PR, Bostian KA (1989). Sequence and mutational analysis of ESS1, a gene essential for growth in Saccharomyces cerevisiae.. Yeast.

[B17] Gemmill TR, Wu X, Hanes SD (2005). Vanishingly Low Levels of Ess1 Prolyl-isomerase Activity Are Sufficient for Growth in Saccharomyces cerevisiae. J Biol Chem.

[B18] Devasahayam G, Chaturvedi V, Hanes SD (2002). The Ess1 Prolyl Isomerase Is Required for Growth and Morphogenetic Switching in Candida albicans. Genetics.

[B19] Huang HK, Forsburg SL, John UP, O'Connell MJ, Hunter T (2001). Isolation and characterization of the Pin1/Ess1p homologue in Schizosaccharomyces pombe.. J Cell Sci.

[B20] Ren P, Rossettini A, Chaturvedi V, Hanes SD (2005). The Ess1 prolyl isomerase is dispensable for growth but required for virulence in Cryptococcus neoformans. Microbiology.

[B21] Maleszka R, Hanes SD, Hackett RL, de Couet HG, Miklos GLG (1996). The Drosophila melanogaster dodo (dod) gene, conserved in humans, is functionally interchangeable with the ESS1 cell division gene of Saccharomyces cerevisiae. Proc Nat Acad Sci USA.

[B22] Edgar KA, Belvin M, Parks AL, Whittaker K, Mahoney MB, Nicoll M, Park CC, Winter CG, Chen F, Lickteig K, Ahmad F, Esengil H, Lorenzi MV, Norton A, Rupnow BA, Shayesteh L, Tabios M, Young LM, Carroll PM, Kopczynski C, Plowman GD, Friedman LS, Francis-Lang HL (2005). Synthetic lethality of retinoblastoma mutant cells in the Drosophila eye by mutation of a novel peptidyl prolyl isomerase gene. Genetics.

[B23] Fujimori F, Takahashi K, Uchida C, Uchida T (1999). Mice Lacking Pin1 Develop Normally, but Are Defective in Entering Cell Cycle from G0 Arrest. Biochem Biophys Res Comm.

[B24] Colgan J, Asmal M, Yu B, Luban J (2005). Cyclophilin A-Deficient Mice Are Resistant to Immunosuppression by Cyclosporine. J Immunol.

[B25] Shou W, Aghdasi B, Armstrong DL, Guo Q, Bao S, Charng MJ, Mathews LM, Schneider MD, Hamilton SL, Matzuk MM (1998). Cardiac defects and altered ryanodine receptor function in mice lacking FKBP12.. Nature.

[B26] Xin HB, Senbonmatsu T, Cheng DS, Wang YX, Copello JA, Ji GJ, Collier ML, Deng KY, Jeyakumar LH, Magnuson MA, Inagami T, Kotlikoff MI, Fleischer S (2002). Oestrogen protects FKBP12.6 null mice from cardiac hypertrophy. Nature.

[B27] Cheung-Flynn J, Prapapanich V, Cox MB, Riggs DL, Suarez-Quian C, Smith DF (2005). Physiological Role for the Cochaperone FKBP52 in Androgen Receptor Signaling. Mol Endocrinol.

[B28] Liou YC, Sun A, Ryo A, Zhou XZ, Yu ZX, Huang HK, Uchida T, Bronson R, Bing G, Li X, Hunter T, Lu KP (2003). Role of the prolyl isomerase Pin1 in protecting against age-dependent neurodegeneration. Nature.

[B29] Liou YC, Ryo A, Huang HK, Lu PJ, Bronson R, Fujimori F, Uchida T, Hunter T, Lu KP (2002). Loss of Pin1 function in the mouse causes phenotypes resembling cyclin D1-null phenotypes. Proc Nat Acad Sci USA.

[B30] Ibrahim AS, Spellberg B, Avanessian V, Fu Y, Edwards JEJ (2005). Rhizopus oryzae adheres to, is phagocytosed by, and damages endothelial cells in vitro. Infect Immun.

[B31] Wilson JM (1979). The biology of Encephalitozoon cuniculi.. Med Biol.

[B32] Kumamoto CA, Vinces MD (2005). Alternative Candida albicans lifestyles: Growth on Surfaces. Ann Rev Microbiol.

[B33] Hazen KC (1995). New and emerging yeast pathogens. Clin Microbiol Rev.

[B34] Marr KA, Patterson T, Denning D (2002). Aspergillosis. Pathogenesis, clinical manifestations, and therapy.. Infect Dis Clin North Am.

[B35] Perfect JR, Casadevall A (2002). Cryptococcosis.. Infect Dis Clin North Am.

[B36] Gold SE, Garcia-Pedrajas MD, Martinez-Espinoza AD (2001). New (and used) approaches to the study of fungal pathogenicity.. Annu Rev Phytopathol.

[B37] Desjardins AE (2003). Gibberella from A (Venaceae) to Z (eae). Ann Rev Phytopathol.

[B38] Feldbrugge M, Kamper J, Steinberg G, Kahmann R (2004). Regulation of mating and pathogenic development in Ustilago maydis. Curr Op Microbiol.

[B39] Cianciotto NP, Fields BS (1992). Legionella pneumophila mip Gene Potentiates Intracellular Infection of Protozoa and Human Macrophages. Proc Nat Acad Sci USA.

[B40] Helbig JH, Konig B, Knospe H, Bubert B, Yu C, Luck CP, Riboldi-Tunnicliffe A, Hilgenfeld R, Jacobs E, Hacker J, Fischer G (2003). The PPIase active site of Legionella pneumophila Mip protein is involved in the infection of eukaryotic host cells.. Biol Chem.

[B41] Lundemose AG, Kay JE, Pearce JH (1993). Chlamydia trachomatis Mip-like protein has peptidyl-prolyl cis/trans isomerase activity that is inhibited by FK506 and rapamycin and is implicated in initiation of chlamydial infection.. Mol Microbiol.

[B42] Denkers EY, Butcher BA, Del Rio L, Bennouna S (2004). Neutrophils, dendritic cells and Toxoplasma. Int J Parasitol.

[B43] Denkers EY (2003). From cells to signaling cascades: manipulation of innate immunity by Toxoplasma gondii. FEMS Immunol Med Microbiol.

[B44] Viaud MC, Balhadere PV, Talbot NJ (2002). A Magnapothe grisea Cyclophilin Acts as a Virulence Determinant during Plant Infection.. Plant Cell.

[B45] Viaud M, Brunet-Simon A, Brygoo Y, Pradier JM, Levis C (2003). Cyclophilin A and calcineurin functions investigated by gene inactivation, cyclosporin A inhibition and cDNA arrays approaches in the phytopathogenic fungus Botrytis cinerea.. Mol Microbiol.

[B46] Wang P, Cardenas ME, Cox GM, Perfect JR, Heitman J (2001). Two cyclophilin A homologs with shared and distinct functions important for growth and virulence of Cryptococcus neoformans.. EMBO Rep.

[B47] Harding MW, Handschumacher RE, Speicher DW (1986). Isolation and amino acid sequence of cyclophilin. J Biol Chem.

[B48] Handschumacher RE, Harding MW, Rice J, Drugge RJ, Speicher DW (1984). Cyclophilin: a specific cytosolic binding protein for cyclosporin A. Science.

[B49] Arevalo-Rodriguez M, Heitman J (2005). Cyclophilin A Is Localized to the Nucleus and Controls Meiosis in Saccharomyces cerevisiae.. Euk Cell.

[B50] Tropschug M, Nicholson DW, Hartl FU, Kohler H, Pfanner N, Wachter E, Neupert W (1988). Cyclosporin A-binding protein (cyclophilin) of Neurospora crassa. One gene codes for both the cytosolic and mitochondrial forms. J Biol Chem.

[B51] Ansari H, Greco G, Luban J (2002). Cyclophilin A Peptidyl-Prolyl Isomerase Activity Promotes Zpr1 Nuclear Export. Mol Cell Biol.

[B52] Pijnappel WWMP, Schaft D, Roguev A, Shevchenko A, Tekotte H, Wilm M, Rigaut G, Seraphin B, Aasland R, Stewart AF (2001). The S. cerevisiae SET3 complex includes two histone deacetylases, Hos2 and Hst1, and is a meiotic-specific repressor of the sporulation gene program. Genes Dev.

[B53] Arevalo-Rodriguez M, Cardenas ME, Wu X, Hanes SD, Heitman J (2000). Cyclophilin A and Ess1 interact with and regulate silencing by the Sin3-Rpd3 histone deacetylase. EMBO J.

[B54] Brown CR, Cui DY, Hung GGC, Chiang HL (2001). Cyclophilin A Mediates Vid22p Function in the Import of Fructose-1,6-bisphosphatase into Vid Vesicles. J Biol Chem.

[B55] Fujimori F, Gunji W, Kikuchi J, Mogi T, Ohashi Y, Makino T, Oyama A, Okuhara K, Uchida T, Murakami Y (2001). Crosstalk of Prolyl Isomerases, Pin1/ESS1, and Cyclophilin A.. Biochem Biophys Res Comm.

[B56] Frigerio G, Pelham HRB (1993). A Saccharomyces cerevisiae Cyclophilin Resident in the Endoplasmic Reticulum. J Mol Biol.

[B57] Joseph JD, Heitman J, Means AR (1999). Molecular Cloning and Characterization of Aspergillus nidulans Cyclophilin B. Fungal Genet Biol.

[B58] Koser PL, Bergsma DJ, Cafferkey R, Eng WK, McLaughlin MM, Ferrara A, Silverman C, Kasyan K, Bossard MJ, Johnson RK (1991). The CYP2 gene of Saccharomyces cerevisiae encodes a cyclosporin A-sensitive peptidyl-prolyl cis-trans isomerase with an N-terminal signal sequence.. Gene.

[B59] Gothel SF, Marahiel MA (1999). Peptidyl-prolyl cis-trans isomerases, a superfamily of ubiquitous folding catalysts.. Cell Mol Life Sci.

[B60] Weisman R, Creanor J, Fantes P (1996). A multicopy suppressor of a cell cycle defect in S. pombe encodes a heat shock inducible 40 kDa cyclophilin-like protein.. EMBO J.

[B61] Mayr C, Richter K, Lilie H, Buchner J (2000). Cpr6 and Cpr7, two closely related HSP90-associated Immunophilins from Saccharomyces cerevisiae, differ in their functional properties.. J Biol Chem.

[B62] Faou P, Tropschug M (2003). A Novel Binding Protein for a Member of CyP40-type Cyclophilins: N. crassa CyPBP37, a Growth and Thiamine Regulated Protein Homolog to Yeast Thi4p. J Mol Biol.

[B63] Sykes K, Gething MJ, Sambrook J (1993). Proline isomerases function during heat shock. Proc Nat Acad Sci USA.

[B64] Duina AA, Kalton HM, Gaber RF (1998). Requirement for Hsp90 and a CyP-40-type cyclophilin in negative regulation of the Heat Shock Response. J Biol Chem.

[B65] Prodromou C, Siligardi G, O'Brien R, Woolfson DN, Regan L, Panaretou B, Ladbury JE, Piper PW, Pearl LH (1999). Regulation of Hsp90 ATPase activity by tetratricopeptide repeat (TPR)-domain co-chaperones. EMBO J.

[B66] Warth R, Briand PA, Picard D (1997). Functional analysis of the yeast 40 kDa cyclophilin Cyp40 and its role for viability and steroid receptor regulation.. Biol Chem.

[B67] Goes FS, Martin J (2001). Hsp90 chaparone complexes are required for the activity and stability of yeast protein kinases Mik1, Wee1 and Swe1.. Eur J Biochem.

[B68] Pemberton TJ, Kay JE (2005). Identification and comparative analysis of the peptidyl-prolyl cis/trans isomerase repertoires of H. Sapiens, D. melanogaster, C. elegans, S. cerevisiae & Sz. pombe.. Comp Funct Genom.

[B69] Ohi MD, Link AJ, Ren L, Jennings JL, McDonald WH, Gould KL (2002). Proteomics analysis reveals stable multiprotein complexes in both Fission and Budding yeasts containing Myb-related Cdc5p/Cef1p, novel pre-mRNA splicing factors, and snRNAs.. Mol Cell Biol.

[B70] McDonald WH, Ohi R, Smelkova N, Frendewey D, Gould KL (1999). Myb-Related Fission Yeast cdc5p Is a Component of a 40S snRNP-Containing Complex and Is Essential for Pre-mRNA Splicing. Mol Cell Biol.

[B71] Pemberton TJ, Rulten SL, Kay JE (2003). Cloning and Characterisation of Schizosaccharomyces pombe Cyclophilin 3: a Cyclosporin A insensitive orthologue of human USA-CyP.. J Chrom B.

[B72] Neer EJ, Schmidt CJ, Nambudripad R, Smith TF (1994). The ancient regulatory-protein family of WD-repeat proteins.. Nature.

[B73] Birney E, Kumar S, Krainer AR (1993). Analysis of the RNA-recognition motif and RS and RGG domains: conservation in metazoan pre-mRNA splicing factors.. Nuc Acids Res.

[B74] Krzywicka A, Beisson J, Keller AM, Cohen J, Jerka-Dziadosz M, Klotz C (2001). KIN241: a gene involved in cell morphogenesis in Paramecium tetraurelia reveals a novel protein family of cyclophilin-RNA interacting proteins (CRIPs) conserved from fission yeast to man.. Mol Microbiol.

[B75] Pringa E, Martinez-Noel G, Muller U, Harbers K (2001). Interaction of the Ring-finger related U-box motif of a nuclear Dot protein with ubiquitin-conjugating enzymes.. J Biol Chem.

[B76] Dolinski K, Scholz C, Muir RS, Rospert S, Schmid FX, Cardenas ME, Heitman J (1997). Functions of FKBP12 and Mitochondrial Cyclophilin Active Site Residues In Vitro and In Vivo in Saccharomyces cerevisiae. Mol Biol Cell.

[B77] Matouschek A, Rospert S, Schmid K, Glick BS, Schatz G (1995). Cyclophilin Catalyzes Protein Folding in Yeast Mitochondria. Proc Nat Acad Sci USA.

[B78] Davis ES, Becker A, Heitman J, Hall MN, Brennan MB (1992). A yeast cyclophilin gene essential for lactate metabolism at high temperature.. Proc Nat Acad Sci USA.

[B79] Rassow J, Mohrs K, Koidl S, Barthelmess IB, Pfanner N, Tropschug M (1995). Cyclophilin 20 is involved in mitochondrial protein folding in cooperation with molecular chaperones Hsp70 and Hsp60. Mol Cell Biol.

[B80] Brustovetsky N, Tropschug M, Heimpel S, Heidkamper D, Klingenberg M (2002). A large Ca2+-dependent channel formed by recombinant ADP/ATP carrier from Neurospora crassa resembles the mitochondrial permeability transition pore.. Biochemistry.

[B81] Skruzny M, Ambrozkova M, Fukova K, Martinkova K, Blahuskova A, Hamplova L, Puta F, Folk P (2001). Cyclophilins of a novel subfamily interact with SNW/SKIP coregulator in Dictyostelium discoideum and Schizosaccharomyces pombe.. Biocheim Biophys Acta.

[B82] Marsh JA, Kalton HM, Gaber RF (1998). Cns1 Is an Essential Protein Associated with the Hsp90 Chaperone Complex in Saccharomyces cerevisiae That Can Restore Cyclophilin 40-Dependant Functions in Cpr7 Deletion Cells.. Mol Cell Biol.

[B83] Tesic M, Marsh JA, Cullinan SB, Gaber RF (2003). Functional interactions between Hsp90 and the Co-chaperones Cns1 and Cpr7 in Saccharomyces cerevisiae. J Biol Chem.

[B84] Weisman R, Finkelstein S, Choder M (2001). Rapamycin blocks sexual development in fission yeast through inhibition of the cellular function of an FKBP12 homolog.. J Biol Chem.

[B85] Cruz MC, Goldstein AL, Blankenship J, Del Poeta M, Perfect JR, McCusker JH, Bennani YL, Cardenas ME, Heitman J (2001). Rapamycin and Less Immunosuppressive Analogs Are Toxic to Candida albicans and Cryptococcus neoformans via FKBP12-Dependent Inhibition of TOR. Antimicrob Agents Chemother.

[B86] Cruz MC, Cavallo LM, Gorlach JM, Cox G, Perfect JR, Cardenas ME, Heitman J (1999). Rapamycin Antifungal Action Is Mediated via Conserved Complexes with FKBP12 and TOR Kinase Homologs in Cryptococcus neoformans. Mol Cell Biol.

[B87] Arevalo-Rodriguez M, Pan X, Boeke JD, Heitman J (2004). FKBP12 controls aspartate pathway flux in Saccharomyces cerevisiae to prevent toxic intermediate accumulation. Euk Cell.

[B88] Partaledis JA, Berlin V (1993). The FKB2 Gene of Saccharomyces cerevisiae, Encoding the Immunosuppressant- Binding Protein FKBP-13, is Regulated in Response to Accumulation of Unfolded Proteins in the Endoplasmic Reticulum. Proc Nat Acad Sci USA.

[B89] Solscheid B, Tropschug M (2000). A novel type of FKBP in the secretory pathway of Neurospora crassa. FEBS Lett.

[B90] Segrest JP, Pownall HJ, Jackson RL, Glenner GG, Pollock PS (1976). Amyloid A: amphipathic helixes and lipid binding.. Biochemistry.

[B91] Benton BM, Zang JH, Thorner J (1994). A novel FK506- and rapamycin-binding protein (FPR3 gene product) in the yeast Saccharomyces cerevisiae is a proline rotamase localized to the nucleolus.. J Cell Biol.

[B92] Manning-Krieg UC, Henriquez R, Cammas F, Graff P, Gaveriaux S, Movva NR (1994). Purification of FKBP-70, a novel immunophilin from Saccharomyces cerevisiae, and cloning of its structural gene, FPR3.. FEBS Lett.

[B93] Shan X, Xue Z, Melese T (1994). Yeast NPI46 encodes a novel prolyl cis-trans isomerase that is located in the nucleolus.. J Cell Biol.

[B94] Davey M, Hannam C, Wong C, Brandl CJ (2000). The yeast peptidyl proline isomerases FPR3 and FPR4, in high copy numbers, suppress defects resulting from the absence of the E3 ubiquitin ligase TOM1.. Mol Gen Genet.

[B95] Hochwagen A, Tham WH, Brar GA, Amon A (2005). The FK506 Binding Protein Fpr3 Counteracts Protein Phosphatase 1 to Maintain Meiotic Recombination Checkpoint Activity. Cell.

[B96] Marchetta M, Gamberi T, Sarno S, Magherini F, Raugei G, Camici G, Pinna LA, Modesti A (2004). Expression of the Stp1 LMW-PTP and inhibition of protein CK2 display a cooperative effect on immunophilin Fpr3 tyrosine phosphorylation and Saccharomyces cerevisiae growth. Cell Mol Life Sci.

[B97] Magherini F, Gamberi T, Paoli P, Marchetta M, Biagini M, Raugei G, Camici G, Ramponi G, Modesti A (2004). The in vivo tyrosine phosphorylation level of yeast immunophilin Fpr3 is influenced by the LMW-PTP Ltp1. Biochem Biophys Res Comm.

[B98] Himukai R, Kuzuhara T, Horikoshi M (1999). Relationship between the subcellular localization and structures of catalytic domains of FKBP-type PPIases. J Biochem (Tokyo).

[B99] Kuzuhara T, Horikoshi M (2004). A nuclear FK506-binding protein is a histone chaperone regulating rDNA silencing.. Nat Struct Mol Biol.

[B100] Xu YX, Hirose Y, Zhou XZ, Lu KP, Manley JL (2003). Pin1 modulates the structure and function of human RNA polymerase II.. Genes Dev.

[B101] Wu X, Wilcox CB, Devasahayam G, Hackett RL, Arevalo-Rodriguez M, Cardenas ME, Heitman J, Hanes SD (2000). The Ess1 prolyl isomerase is linked to chromatin remodeling complexes and the general transcription machinery. EMBO J.

[B102] Wu X, Rossettini A, Hanes SD (2003). The ESS1 prolyl isomerase and its suppressor BYE1 interact with RNA Pol II to inhibit transcription elongation in Saccharomyces cerevisiae. Genetics.

[B103] Morris DP, Phatnani HP, Greenleaf AL (1999). Phospho-Carboxyl-Terminal Domain Binding and the Role of a Prolyl Isomerase in Pre-mRNA 3'-End Formation. J Biol Chem.

[B104] Kops O, Zhou XZ, Lu KP (2002). Pin1 modulates the dephosphorylation of the RNA polymerase II C-terminal domain by yeast Fcp1. FEBS Lett.

[B105] Wilcox CB, Rossettini A, Hanes SD (2004). Genetic Interactions With C-Terminal Domain (CTD) Kinases and the CTD of RNA Pol II Suggest a Role for ESS1 in Transcription Initiation and Elongation in Saccharomyces cerevisiae. Genetics.

[B106] Jeong SJ, Kim HJ, Yang YJ, Seol JH, Jung BY, Han JW, Lee HW, Cho EJ (2005). Role of RNA polymerase II carboxy terminal domain phosphorylation in DNA damage response.. J Microbiol.

[B107] Metzner M, Stoller G, Rucknagel KP, Lu KP, Fischer G, Luckner M, Kullertz G (2001). Functional Replacement of the Essential ESS1 in Yeast by the Plant Parvulin DlPar13. J Biol Chem.

[B108] Li Z, Li H, Devasahayam G, Gemmill T, Chaturvedi V, Hanes SD, Van Roey P (2005). The Structure of the Candida albicans Ess1 Prolyl Isomerase Reveals a Well-Ordered Linker that Restricts Domain Mobility.. Biochemistry.

[B109] Kops O, Eckerskorn C, Hottenrott S, Fischer G, Mi H, Tropschug M (1998). Ssp1, a Site-specific Parvulin Homolog from Neurospora crassa Active in Protein Folding. J Biol Chem.

[B110] Galat A (2003). Peptidylprolyl cis/trans isomerases (immunophilins): biological diversity--targets--functions.. Curr Top Med Chem.

[B111] Maruyama T, Suzuki R, Furutani M (2004). Archaeal peptidyl prolyl cis-trans isomerases (PPIases) update 2004.. Front Biosci.

[B112] Hughes AL (1994). The evolution of functionally novel proteins after gene duplication.. Proc Biol Sci.

[B113] Lynch M, Conery JS (2000). The Evolutionary Fate and Consequences of Duplicate Genes. Science.

[B114] Friedman R, Hughes AL (2001). Pattern and Timing of Gene Duplication in Animal Genomes. Genome Res.

[B115] Dujon B, Sherman D, Fischer G, Durrens P, Casaregola S, Lafontaine I, de Montigny J, Marck C, Neuveglise C, Talla E, Goffard N, Frangeul L, Aigle M, Anthouard V, Babour A, Barbe V, Barnay S, Blanchin S, Beckerich JM, Beyne E, Bleykasten C, Boisrame A, Boyer J, Cattolico L, Confanioleri F, de Daruvar A, Despons L, Fabre E, Fairhead C, Ferry-Dumazet H, Groppi A, Hantraye F, Hennequin C, Jauniaux N, Joyet P, Kachouri R, Kerrest A, Koszul R, Lemaire M, Lesur I, Ma L, Muller H, Nicaud JM, Nikolski M, Oztas S, Ozier-Kalogeropoulos O, Pellenz S, Potier S, Richard GF, Straub ML, Suleau A, Swennen D, Tekaia F, Wesolowski-Louvel M, Westhof E, Wirth B, Zeniou-Meyer M, Zivanovic I, Bolotin-Fukuhara M, Thierry A, Bouchier C, Caudron B, Scarpelli C, Gaillardin C, Weissenbach J, Wincker P, Souciet JL (2004). Genome evolution in yeasts. Nature.

[B116] Fischer G, Rocha EPC, Brunet F, Vergassola M, Dujon B, ric (2006). Highly Variable Rates of Genome Rearrangements between Hemiascomycetous Yeast Lineages. PLoS Genetics.

[B117] Wolfe KH, Shields DC (1997). Molecular evidence for an ancient duplication of the entire yeast genome. Nature.

[B118] Langkjaer RB, Cliften PF, Johnston M, Piskur J (2003). Yeast genome duplication was followed by asynchronous differentiation of duplicated genes. Nature.

[B119] Wolfe K (2004). Evolutionary Genomics: Yeasts Accelerate beyond BLAST. Current Biology.

[B120] Kellis M, Birren BW, Lander ES (2004). Proof and evolutionary analysis of ancient genome duplication in the yeast Saccharomyces cerevisiae. Nature.

[B121] Scannell DR, Byrne KP, Gordon JL, Wong S, Wolfe KH (2006). Multiple rounds of speciation associated with reciprocal gene loss in polyploid yeasts. Nature.

[B122] Byrne KP, Wolfe KH (2005). The Yeast Gene Order Browser: Combining curated homology and syntenic context reveals gene fate in polyploid species. Genome Res.

[B123] Vogel C, Teichmann SA, Pereira-Leal J (2005). The Relationship Between Domain Duplication and Recombination. Journal of Molecular Biology.

[B124] Bjorklund AK, Ekman D, Light S, Frey-Skott J, Elofsson A (2005). Domain Rearrangements in Protein Evolution. Journal of Molecular Biology.

[B125] Bashton M, Chothia C (2002). The geometry of domain combination in proteins. Journal of Molecular Biology.

[B126] Apic G, Gough J, Teichmann SA (2001). Domain combinations in archaeal, eubacterial and eukaryotic proteomes. Journal of Molecular Biology.

[B127] Homann OR, Cai H, Becker JM, Lindquist SL (2005). Harnessing Natural Diversity to Probe Metabolic Pathways. PLoS Genetics.

[B128] Mager WH, Winderickx J (2005). Yeast as a model for medical and medicinal research. Trends in Pharmacological Sciences.

[B129] Mustacchi R, Hohmann S, Nielsen J (2006). Yeast systems biology to unravel the network of life. Yeast.

[B130] Arevalo-Rodriguez M, Wu X, Hanes SD, Heitman J (2004). Prolyl isomerases in yeast.. Front Biosci.

[B131] The National Centre for Biotechnology Information (NCBI). http://www.ncbi.nlm.nih.gov/.

[B132] Wood V, Gwilliam R, Rajandream MA, Lyne M, Lyne R, Stewart A, Sgouros J, Peat N, Hayles J, Baker S, Basham D, Bowman S, Brooks K, Brown D, Brown S, Chillingworth T, Nurse P (2002). The genome sequence of Schizosaccharomyces pombe. Nature.

[B133] Katinka MD, Duprat S, Cornillot E, Metenier G, Thomarat F, Prensier G, Barbe V, Peyretaillade E, Brottier P, Wincker P, Delbac F, El Alaoui H, Peyret P, Saurin W, Gouy M, Weissenbach J, Vivares CP (2001). Genome sequence and gene compaction of the eukaryote parasite Encephalitozoon cuniculi. Nature.

[B134] Goffeau A, Barrell BG, Bussey H, Davis RW, Dujon B, Feldmann H, Galibert F, Hoheisel JD, Jacq C, Johnston M, Louis EJ, Mewes HW, Murakami Y, Philippsen P, Tettelin H, Oliver SG (1996). Life with 6000 genes.. Science.

[B135] Wood V, Rutherford KM, Ivens A, Rajandream MA, Barrell B (2001). A re-annotation of the Saccharomyces cerevisiae Genome.. Comp Funct Genomics.

[B136] Dietrich FS, Voegeli S, Brachat S, Lerch A, Gates K, Steiner S, Mohr C, Pohlmann R, Luedi P, Choi S, Wing RA, Flavier A, Gaffney TD, Philippsen P (2004). The Ashbya gossypii Genome as a Tool for Mapping the Ancient Saccharomyces cerevisiae Genome. Science.

[B137] Jones T, Federspiel NA, Chibana H, Dungan J, Kalman S, Magee BB, Newport G, Thorstenson YR, Agabian N, Magee PT, Davis RW, Scherer S (2004). The diploid genome sequence of Candida albicans. Proc Nat Acad Sci USA.

[B138] Galagan JE, Calvo SE, Cuomo C, Ma LJ, Wortman JR, Batzoglou S, Lee SI, Basturkmen M, Spevak CC, Clutterbuck J, Kapitonov V, Jurka J, Scazzocchio C, Farman M, Butler J, Purcell S, Harris S, Braus GH, Draht O, Busch S, D'Enfert C, Bouchier C, Goldman GH, Bell-Pedersen D, Griffiths-Jones S, Doonan JH, Yu J, Vienken K, Pain A, Freitag M, Selker EU, Archer DB, Penalva MA, Oakley BR, Momany M, Tanaka T, Kumagai T, Asai K, Machida M, Nierman WC, Denning DW, Caddick M, Hynes M, Paoletti M, Fischer R, Miller B, Dyer P, Sachs MS, Osmani SA, Birren BW (2005). Sequencing of Aspergillus nidulans and comparative analysis with A. fumigatus and A. oryzae. Nature.

[B139] Nierman WC, Pain A, Anderson MJ, Wortman JR, Kim HS, Arroyo J, Berriman M, Abe K, Archer DB, Bermejo C, Bennett J, Bowyer P, Chen D, Collins M, Coulsen R, Davies R, Dyer PS, Farman M, Fedorova N, Fedorova N, Feldblyum TV, Fischer R, Fosker N, Fraser A, Garcia JL, Garcia MJ, Goble A, Goldman GH, Gomi K, Griffith-Jones S, Gwilliam R, Haas B, Haas H, Harris D, Horiuchi H, Huang J, Humphray S, Jimenez J, Keller N, Khouri H, Kitamoto K, Kobayashi T, Konzack S, Kulkarni R, Kumagai T, Lafton A, Latge JP, Li W, Lord A, Lu C, Majoros WH, May GS, Miller BL, Mohamoud Y, Molina M, Monod M, Mouyna I, Mulligan S, Murphy L, O'Neil S, Paulsen I, Penalva MA, Pertea M, Price C, Pritchard BL, Quail MA, Rabbinowitsch E, Rawlins N, Rajandream MA, Reichard U, Renauld H, Robson GD, de Cordoba SR, Rodriguez-Pena JM, Ronning CM, Rutter S, Salzberg SL, Sanchez M, Sanchez-Ferrero JC, Saunders D, Seeger K, Squares R, Squares S, Takeuchi M, Tekaia F, Turner G, de Aldana CRV, Weidman J, White O, Woodward J, Yu JH, Fraser C, Galagan JE, Asai K, Machida M, Hall N, Barrell B, Denning DW (2005). Genomic sequence of the pathogenic and allergenic filamentous fungus Aspergillus fumigatus. Nature.

[B140] Galagan JE, Calvo SE, Borkovich KA, Selker EU, Read ND, Jaffe D, FitzHugh W, Ma LJ, Smirnov S, Purcell S, Rehman B, Elkins T, Engels R, Wang S, Nielsen CB, Butler J, Endrizzi M, Qui D, Ianakiev P, Bell-Pedersen D, Nelson MA, Werner-Washburne M, Selitrennikoff CP, Kinsey JA, Braun EL, Zelter A, Schulte U, Kothe GO, Jedd G, Mewes W, Staben C, Marcotte E, Greenberg D, Roy A, Foley K, Naylor J, Stange-Thomann N, Barrett R, Gnerre S, Kamal M, Kamvysselis M, Mauceli E, Bielke C, Rudd S, Frishman D, Krystofova S, Rasmussen C, Metzenberg RL, Perkins DD, Kroken S, Cogoni C, Macino G, Catcheside D, Li W, Pratt RJ, Osmani SA, DeSouza CPC, Glass L, Orbach MJ, Berglund JA, Voelker R, Yarden O, Plamann M, Seiler S, Dunlap J, Radford A, Aramayo R, Natvig DO, Alex LA, Mannhaupt G, Ebbole DJ, Freitag M, Paulsen I, Sachs MS, Lander ES, Nusbaum C, Birren B (2003). The genome sequence of the filamentous fungus Neurospora crassa. Nature.

[B141] Loftus BJ, Fung E, Roncaglia P, Rowley D, Amedeo P, Bruno D, Vamathevan J, Miranda M, Anderson IJ, Fraser JA, Allen JE, Bosdet IE, Brent MR, Chiu R, Doering TL, Donlin MJ, D'Souza CA, Fox DS, Grinberg V, Fu J, Fukushima M, Haas BJ, Huang JC, Janbon G, Jones SJM, Koo HL, Krzywinski MI, Kwon-Chung JK, Lengeler KB, Maiti R, Marra MA, Marra RE, Mathewson CA, Mitchell TG, Pertea M, Riggs FR, Salzberg SL, Schein JE, Shvartsbeyn A, Shin H, Shumway M, Specht CA, Suh BB, Tenney A, Utterback TR, Wickes BL, Wortman JR, Wye NH, Kronstad JW, Lodge JK, Heitman J, Davis RW, Fraser CM, Hyman RW (2005). The Genome of the Basidiomycetous Yeast and Human Pathogen Cryptococcus neoformans. Science.

[B142] Broad Institute of MIT & Harvard (2006). Fusarium graminearum Sequencing Project. Broad Institute of MIT and Harvard (http://www.broad.mit.edu).

[B143] Guldener U, Mannhaupt G, Munsterkotter M, Haase D, Oesterheld M, Stumpflen V, Mewes HW, Adam G (2006). FGDB: a comprehensive fungal genome resource on the plant pathogen Fusarium graminearum. Nuc Acids Res.

[B144] Broad Institute of MIT & Harvard (2006). Ustilago maydis Sequencing Project. Broad Institute of MIT and Harvard (http://www.broad.mit.edu).

[B145] Broad Institute of MIT & Harvard (2006). Rhizopus oryzae Sequencing Project. Broad Institute of MIT and Harvard (http://www.broad.mit.edu).

[B146] Altschul SF, Madden TL, Schaffer AA, Zhang J, Zhang Z, Miller W, Lipman DJ (1997). Gapped BLAST and PSI-BLAST: a new generation of protein database search programs. Nuc Acids Res.

[B147] Galat A (2000). Sequence diversification of the FK506-binding proteins in several different genomes.. Eur J Biochem.

[B148] Galat A (2004). Function-dependent clustering of orthologues and paralogues of cyclophilins.. Proteins.

[B149] Universal Protein Resource (UniProt). http://www.uniprot.org/.

[B150] Marchler-Bauer A, Panchenko AR, Shoemaker BA, Thiessen PA, Geer LY, Bryant SH (2002). CDD: a database of conserved domain alignments with links to domain three-dimensional structure.. Nuc Acids Res.

[B151] NCBI Conserved Domain Database (CDD). http://www.ncbi.nlm.nih.gov/Structure/cdd/wrpsb.cgi.

[B152] Horton P, Nakai K (1996). A probabilistic classification system for predicting the cellular localization sites of proteins.. Proc Int Conf Intell Syst Mol Biol.

[B153] Horton P, Nakai K (1997). Better prediction of protein cellular localization sites with the k nearest neighbors classifier.. Proc Int Conf Intell Syst Mol Biol.

[B154] National Institute for Basic Biology (NIBB) PSORT server. http://psort.nibb.ac.jp/.

[B155] Expert Protein Analysis System (ExPASy) PI/Mw calculation tool. http://www.expasy.ch/tools/pi_tool.html.

[B156] Campanella J, Bitincka L, Smalley J (2003). MatGAT: An application that generates similarity/identity matrices using protein or DNA sequences. BMC Bioinformatics.

[B157] Thompson JD, Gibson TJ, Plewniak F, Jeanmougin F, Higgins DG (1997). The ClustalX windows interface: flexible strategies for multiple sequence alignment aided by quality analysis tools.. Nuc Acids Res.

[B158] Saitou N, Nei M (1987). The Neighbor-joining Method: A New Method for Reconstructing Phylogenetic Trees.. Mol Biol Evol.

[B159] Kumar S, Tamura K, Nei M (2004). MEGA3: Integrated software for Molecular Evolutionary Genetics Analysis and sequence alignment.. Brief Bioinform.

[B160] Tatusov RL, Koonin EV, Lipman DJ (1997). A genomic perspective on protein families. Science.

[B161] Koonin EV, Tatusov RL, Rudd KE (1995). Sequence Similarity Analysis of Escherichia coli Proteins: Functional and Evolutionary Implications. PNAS.

[B162] Koonin EV, Tatusov RL, Rudd KE (1996). Protein sequence comparison at genome scale. Methods In Enzymology.

[B163] Cai J, Roberts IN, Collins MD (1996). Phylogenetic relationships among members of the ascomycetous yeast genera Brettanomyces, Debaryomyces, Dekkera, and Kluyveromyces deduced by small-subunit rRNA gene sequences. Int J Syst Bacteriol.

