# Supplementary material for: Identification and comparative analysis of sixteen fungal peptidyl-prolyl cis/trans isomerase repertoires
Source: BMC Genomics. 2006 Sep 22;7:244. doi: 10.1186/1471-2164-7-244 (PMC1618848; doi:10.1186/1471-2164-7-244)

Cyclophilin - Group A

```
                  :   *::*.: :***  ***:**.**  *. .***:.. ***:* :*::* **********:**:*::* *** . *:..***:*:***:*****.****:* ****:*********** .*:   :*:  : *. .**     **  *
AnCyp2 ----MAT--DVTFDTSMGSFTVELYNSHAPKTCKNFATLAQRGYYNNVIFHRIIPNFMVQTGDPTGTGRGGSSIYGEKFEDEIRSDLKHTGAGILSMANSGPNTNGSQFFITLAPTPWLDGKHTIFGRVKSGMRIIQRMGLVKTNNEDRPLDEVKILRAKVVEAGSEE--   162
AfCyp1 ----MAT--DVVFDTSMGSFTVELYNEHAPKTCRNFATLAQRGYYNNVIFHRIIPNFMVQTGDPTGTGRGGSSIYGEKFEDEIHPGLKHTGAGVLSMANSGPNTNGSQFFITLAPTPWLDGKHTIFGRVKSGMRVIQRMGLVKTNSEDRPVDEVKIIRAKVVEEGDE---   161
NcCyp1 ----MVP--YLVPLTSMGTFTLELYTNHAPKTCKNFATLADRGYYDSTVFHRIIKDFMIQGGDPTGTGRGGSSIYGEKFEDEIHPGLKHTGAGVLSMANAGPNTNGSQFFITLAPTPWLDGKHTIFGRVKKGMGVIRRMGMVPTDKEDRPATEVKIVKARVVREEDMEA-   163
GzCyp1 ----MTT--NIVLETTMGSLILELYTTHAPKTCNNFTTLVRRGYYDNTIFHRIIPNFMVQGGDPTGTGRGGSSIFGEKFEDEIDPGLKHTGAGILSMANAGPNTNGSQFFVTLAPTPWLDGKHTIFGRVKSGMGTIKRMGLVKTGSEDRPVEEVKIVKARVVEEEEQI--   162
CnCyp2 MSGPSPT--YVTFDTSVGSFTVELYTAHAPKTCNNFAKLAERGYYNGVIFHRIIPNFMIQGGDPTGTGRGGTSIYGDRFADEIHPELRFVGAGILAMANSGPNTNGSQFFITCAPTPYLDGKHTIFGRVSSGMKTIQRLEAVRTDKDDRPVEEIKIHRARLGDATQGGGL   168
SpCyp1 ----MAN---VELQTSLGKILIELYTEHAPKTCQNFYTLAKEGYYDGVIFHRVIPDFVIQGGDPTGTGRGGTSIYGDKFDDEIHSDLHHTGAGILSMANAGPNTNSSQFFITLAPTPWLDGKHTIFGRVVSGLSVCKRMGLIRTDSSDRPIEPLKIIKAVAL--------   155
RoCyp3 ----MSEREEVILDTSMGSIHIELYWDHAPRTCKNFYELAKRGYYDGVSFHRIIADFMIQGGDPTGTGRGGTSIYGERFADEINPGLQHTGAGILSMANAGPNTNGSQFFITLAPTPWLDGKHTIFGRVSDGMNVVKRMGLVKTDANDRLFIVQKILYALNAVDLNKEL-   165
 ruler 1.......10........20........30........40........50........60........70........80........90.......100.......110.......120.......130.......140.......150.......160.......170

AnCyp2 ------   162
AfCyp1 ------   161
NcCyp1 ------   163
GzCyp1 ------   162
CnCyp2 AVAMPA   174
SpCyp1 ------   155
RoCyp3 ------   165
 ruler ......
```

Cyclophilin - Group B

```
                      :**:  :                   *:: : * :::.***. **: *.   :* ** .. ***:** **:****:*   *.**:**** :* ****   * .  :*****.* :*******:*     **:  * ***:* .    .:
NcCyp4_iso.2 -----------------MS-KVFFDLEWEGPVLGPNNKPTSEIKAQSGRINFTLYDDVVPKTARNFKELCTGQNGFGYKGSSFHRIIPEFMLQGGDFTRGNGTGGKSIYGEKFADENFAKKHVRPGLLSMANAGPNTNGSQFFVTTVPTSWLDGRHVVFGEVADDESMKV   152
GzCyp5_iso.2 -----------------MGNKVFFDISWEGPVFQ-NGKPTSEVKKQTGRINFNLFDDVVPKTAENFRALCTGEKGFGYEGSSFHRIIPDFMLQGGDFTRGNGTGGKSIYGEKFADENFTLKHDKPGLLSMANAGPNTNGSQFFVTTVVTSWLNGRHVVFGEVADEESLNV   152
      AfCyp2 -----------------MVVKTFFDITIDG--------------QPAGRITFKLFDEVVPKTVENFRALCTGEKGFGYKGSSFHRIIPQFMLQGGDFTKGNGTGGKSIYGDRFPDENFQLKHDKPGLLSMANAGKNTNGSQFFITTVVTSWLDGAHVVFGEVEDG--MDL   137
      RoCyp1 -----------------MS--VYFDISIDG--------------KPAGRIEFQLFEDVVPKTAKNFRALCTGEQGFGYKGSKFHRVIPQFMLQGGDFTRGDGTGGKSIYGEKFADENFKLKHSEPGLLSMANAGPNTNGSQFFITTVPCSWLDGAHVVFGKVTKN--MEL   135
      CnCyp3 MSSMLLSHLCNVELIVFNS-QVYFDIAINN--------------APAGRITFKLFDDVVPKTARNFRELCTGQNGFGYAGSGFHRVIPQFMLQGGDFTNHNGTGGKSIYGNKFADENFKLRHDRPFLLSMANAGPNTNGSQFFITTVVTSWLDGKHCVFGEVSSG--QDL   153
      UmCyp1 -----------------MS-NVFFDITKNG--------------SPLGRITFKLYDDVVPKTAKNFRELCTGAHGFGYEGSSFHRVIPDFMLQGGDFTAGNGTGGKSIYGAKFEDENFKLSHTKPGLLSMANAGPNTNGSQFFITTVVTSWLDGKHVVFGEVVEG--LDI   136
      AnCyp1 -----------------MS-NVFFDITANG--------------EPLGRVEFKLFDDVVPKTARNFRELATGQHGFGYKGSPFHRVIPQFMLQGGDFTRQNGTGGKSIYGEKFEDENFTLKHDRPYLLSMANAGRNTNGSQFFITTVKTSWLDGAHVVFGEVVKG--QEV   136
      EgCyp1 -----------------MS-KVFFDVEADG--------------QPLGRIVFQLYNDVTPKTAENFRALCTGEKGFGYAGCPFHRVIPDFMLQGGDFTHGTGVGGKSIYGGKFPDENFVKKHEKPGLLSMANAGPNTNGSQFFITTVPCPWLNGKHVVFGEVSEG--MDV   136
      KlCyp1 -----------------MS-KVFFDVEADG--------------QPLGRIVFQLYNDVVPKTTENFRALATGEKGFGYAGSVFHRVIPDFMLQGGDFTHGTGVGGKSIYGAKFPDENFVKKHEKPGLLSMANAGPNTNGSQFFITTVPCPWLDGKHVVFGEVADG--FDV   136
      CgCyp1 -----------------MS-KVYFDIEADG--------------QPLGRVVFQLYDDVVPKTAANFRALCTGEKGFGYAGSPFHRVIPDFMLQGGDFTAGNGTGGKSIYGGKFPDENFVKRHERPGLLSMANAGPNTNGSQFFITTVPCPWLDGKHVVFGEVSEG--YDV   136
      ScCpr1 -----------------MS-QVYFDVEADG--------------QPIGRVVFKLYNDIVPKTAENFRALCTGEKGFGYAGSPFHRVIPDFMLQGGDFTAGNGTGGKSIYGGKFPDENFKKHHDRPGLLSMANAGPNTNGSQFFITTVPCPWLDGKHVVFGEVVDG--YDI   136
      DhCyp1 -----------------MS-QVYFDISADG--------------NKLGRVTFKLYDDVVPKTAENFRALCTGEKGFGYASSTFHRVIPQFMLQGGDFTRGNGTGGKSIYGEKFRDENFVKKHEKPGLLSMANAGPNTNGSQFFITTVTCPWLDGKHVVFGEVVDG--FDI   136
      CaCyp1 -----------------MS-TVYFDVSADG--------------QKLGKITFKLYDDVVPKTAENFRALCTGEKGFGYKGSIFHRVIPQFMLQGGDFTNFNGTGGKSIYGTKFADENFVKRHDRPGLLSMANAGPNTNGSQFFITTVPCPWLDGKHVVFGEVTDG--LDI   136
      YlCyp1 -----------------MSNQVYFDVSADG--------------QPLGRIEFKLYNDIVPKTAENFRALCTGEQGFGYANSIFHRVIPQFMLQGGDFTRHNGTGGKSIYGAKFPDENFEAKHTRPGLLSMANSGPNTNGSQFFVTTVPCPWLDGKHVVFGEVTNG--MDV   137
      SpCyp2 -----------------MS-NCFFDVIANG--------------QPLGRIVFKLFDDVVPKTAANFRALCTGEKGYGYAGSTFHRVIPQFMLQGGDFTRGNGTGGKSIYGEKFPDENFALKHNKPGLLSMANAGPNTNGSQFFITTVVTPWLDGKHVVFGEVTEG--MDV   136
      EcCyp1 -------------MAKEASGNVYFDVYANE--------------ESLGRIVMKLEDDIVPKTAKNFRTLCERPKGEGYKGSTFHRIIPGFMVQGGDYTAHNGTGGRSIYGEKFPDENFELKHTKEGILSMANCGAHTNGSQFFITLGKTQWLDEKHVVFGEVVEG--MDV   141
       ruler 1.......10........20........30........40........50........60........70........80........90.......100.......110.......120.......130.......140.......150.......160.......170

             *  :   *: **       :  :  .* :
NcCyp4_iso.2 VKALEATGSSSGAIRYSKKPTIVDCGAL---    180
GzCyp5_iso.2 VKALEATGSGSGAIKYTQKPKIEKSGEL---    180
      AfCyp2 VKKIESYGSASGTPK--KKITIADCGQL---    163
      RoCyp1 VSKIESLGSASGAVK--AKVVIEDCGVL---    161
      CnCyp3 VRKIESYGSDSGKPK--AKITITASGTV---    179
      UmCyp1 VKAIENEGTKSGKTK--SKITIAKSGVL---    162
      AnCyp1 VDAVEKLGSQSGATK--KKVVISNSGTL---    162
      EgCyp1 VKKVESMGSSSGATR--ARIVISKSGEL---    162
      KlCyp1 VKKVESLGSGSGATK--ARIVIAKSGEL---    162
      CgCyp1 VKKVESMGSSSGATR--ARIIVSKSGEL---    162
      ScCpr1 VKKVESLGSPSGATK--ARIVVAKSGEL---    162
      DhCyp1 VKKVESYGSGSGATK--SRITIDASGEL---    162
      CaCyp1 VKKIESFGSGSGATS--KKIVIEESGQL---    162
      YlCyp1 VKKIESFGSSSGATR--AKISIDKSGEL---    163
      SpCyp2 VKKVESLGSNSGATR--ARIVIDKCGTV---    162
      EcCyp1 VHKIAKYGSESGQVKKGYRIEIRDCGVLGSN    172
       ruler .......180.......190.......200.
```

Cyclophilin - Group C

```
       *******  **:*:*::*::.*::****:. *..* *:   :** :  ** *         .. .              *** **:    : :**:  :*:.. **::: *.               *.  * ******:   * **   :::*:*:      :   .*
CnCyp1 MSVTLHTNLGDIKIELFCESVPRTAENFLALCASGQYDGTLFHRNIRGFMIQ------GGDPTGT-------------GKGGQSIWG-RPFSDEIR-QTLRFNNRGMVAMAN--------------AGPDTNKSQFFITYAKQPSLDGKYSIFGKVID---GME---TLD   129
UmCyp2 MSVTLHTNVGDIKVEVFCDATPRAAENFLGHCAAGTYNDVKWHRNIKSFMIQ------TGDPTGT-------------GKGGQSIWG-KPFADEIR-SSLKFDRRGIVACAN--------------AGPDTNKSQFFITYAKQPHLDGKYTIIGKVID---GAEDGGTLD   132
RoCyp4 MSVTLHTDLGDIKIEVFCEAVPKTAENFLALCASGYYDNNTFHRNIPGFMIQVHEHLHTGDPTGT-------------GKGGNSIWG-KKFNDEIR-STLKHNSRGIVSMAN--------------SGPNTNGSQFFITYAKHPHLDTKYTVFGKVID---GADS--TLD   136
GzCyp2 MSVTLHTSHGDIKVEIYCESVPKTAENFLALCGSGYYDKSPFHRVIPKFMAQTG-APATPNPPEN-------------PKGGRSIWG-GAFEDEIR-PALRHGARGVLSMAN--------------KGPGTNGSQFFITFDKAPHLDGLNTVFGRVI----GDEGLATLA   136
AnCyp5 MSVTLHTTHGDLKVELFCEAVPKTAENFIALCAAGAYNDTPFHRLIPGFMIQGG-DISLGPAANSQGTT-PMLPFDDIPKGGTSIYHPSALNQEIHLPALRHNTRGILSMASRPVKNQTAPGSQGATGPTINGSQFFITFAAAPHLDGSSTVFGKVLNLTAEDEGGDVLS   168
AfCyp5 MSVTLHTTHGDLKVELFCEAVPQTAENFLALCACGAYNNTPFHRIFPGFMIQGG-DISLGPAPGTLNTLKPMLPVNEIPKGGTSIYHPRALNQEIHLPALRHNARGILSMASRPVKDRTAPGSQGATGATVNGSQFFITFAAAPHLDGASTVFGKVLNLSPQDEGGDVLS   169
 ruler 1.......10........20........30........40........50........60........70........80........90.......100.......110.......120.......130.......140.......150.......160.......170

        :*     .: * :  .           . :  **:****:*
CnCyp1 SMEKT--PVNPKSRPLQE----------IKLLNVTVHANPIADQAKGGLA   167
UmCyp2 AMEAI--PVDAKNRPTSE----------IRMTGVTVHANPIAIKAAGK--   168
RoCyp4 MMEKV--PVDEKHRPLQE----------FRIKSVTIHANPIADKQL----   170
GzCyp2 KMEAV--EVDRKNRPKEP----------VRIENVTIHANPLAG-------   167
AnCyp5 KLEKANVKTDKKGKVVQPKENEETEYETLRINRVTIHANPFAT-------   211
AfCyp5 KLEKAKLKVDKKGRVTQPKEGEEGGYEAIGINSVTIHANPFAK-------   212
 ruler .......180.......190.......200.......210.......220
```

Cyclophilin - Group D

```
                                    **:. .*: .  *:*.****::****       * *** . ***::  ** *****: .:* *:  *:.   * **    .    *     :****:* .:**.****       *:..* ***.:.:*   :  :* . .
UmCyp4 --------------------------TPAGRLKCELFSDIVPRTSENFRQLCTGEFRPN-HVPEGYKNSIFHRIIKDFMCQGGDFINADGTGSRSIYG-DKFDDE----NFTLKHDKAGLLSMANSGPGTNGCQFFITAQPCPFLDGKHVVFGKVVDGLLTLRKMENVPT   138
RoCyp7 MDKKSNQV-----ERPVVFFDISIGDVPVGRMKMELFSDIVPRTAENFRQLCTGEYKRN-GVPQGYKNCLFHRVIKDFMVQGGDFIKGDGTGAMCIYGGDRFADE----NFIEKHTGAGLLSMANSGPNSNGCQFFITCDACDFLDGKHVVFGRLVDGLLTLRKIENVAT   160
CnCyp4 MSSIDPPAG---HTRPIVFFDISIGDTPAGRIKMELFDDITPKTAENFRQLCTGEHRIN-SVPQGYKKATFHRVIPQFMVQGGDFVRGDGTGSFSIYG-AQFEDE----NFKVKHTGPGLLSMANSGPNTNGCQFFITTAPAEFLDGKHCVFGRVIDGLLTVRKIENVPT   161
NcCyp2 MAPTVLPA----SGNPLVFFDITLGGEPLGRITFELFKDVVPRTAENFRQFCTGESKNNLGRPQGYKGSKFHRIIPNFMCQGGDFLNGDGTGSTCIYGTKSFADE----NFLLKHDTPGLLSMANAGPNTNGSQFFITTVPTPFLDGKHVVFGKVVDGMDVVKKMENTKT   162
GzCyp3 MPPTKLPE----SGNPLVFFDITIGGEPLGRITFELFKDVVPKTAENFRQFCTGESKTPVGRPQGYKGSKFHRIIPNFMCQGGDFLNGDGTGSTCIWGFKSFEDE----NFTLKHDQPGLLSMANAGPNTNGSQFFITTVPTPFLDNKHVVFGKVFEGMDVVKKMEATKT   162
AnCyp4 MSNTGSAVPQTTSSNPIVFFDVALGGEPLGRLKLELFADVTPRTAENFRQFCTGESKNQQGRPQGYKGSKFHRVIKDFMIQGGDFVNGDGTGSCTIYGTPKFSDE----NFTLRHDRPGLLSMANSGPNTNGCQFFITTTATPFLNGKHVVFGQVIDGMDIVRMIENTRT   166
AfCyp3 MEDIHSGP--ATNTNPIVFFDIALGGEPLGRIKMELFADVTPRTAENFRRFCTGESKNSQGKPQGYKNSKFHRVIKDFMIQGGDFVNGDGTGSCTIYGTPKFADE----NFVLKHDRAGVLSMANSGPNTNGCQFFITTTATPFLNGKHVVFGQVVDGMDIVRMIENTRT   164
SpCyp3 MS-----------TEPVVFMDIAIDGRLLGRIKIRLFSSIVPKTAENFRQFCTGETLGVNQKPIGYKNSTFHRIIQGFMIQGGDFVSGDGTGSATIFNSRTFPDE----NFTLKHDRPGLLSMANAGKDSNGCQFFITTVPCDFLDGKHVVFGEVIEGYDIVKEIESTPV   155
DhCyp2 -------------MNPVVFLDVNKGDVPLGRIKLKLFASELPKTCENFRQFCTGEYREN-NRPKGYKGCTFHRVVKGFMIQGGDFVRGNGLGSISIYGGNTFDDE----GFPYDH-KKYSVSMANSGPNSNGCQFFICCKEVPHLDGKHVVFGEVVEGFDIVDQIEKTKV   151
YlCyp2 -------------MT-ITFLDVTYDGQRIGRIKFKLYSEDLPKTCENFRQFCTGEYRID-NVPQGYKESTFHRVVKGFMIQGGDFVRGNGLGTVSIFGSASFPDERTISGKTFPH-IEGALSMANSGKDTNGCQFFITTRKCPELDGSHVVFGHVIEGMDVVKFVENVPV   154
 ruler 1.......10........20........30........40........50........60........70........80........90.......100.......110.......120.......130.......140.......150.......160.......170

            . *   : : :**:
UmCyp4 G--ANNRPKMAVRITQCGEM   156
RoCyp7 G--PNNRPKLPVKITECGQM   178
CnCyp4 G--ANNRPKLQVRIAECGEM   179
NcCyp2 GYRGKDVPNLDVVIAQCGEM   182
GzCyp3 GYRGKDMPNLDVVISQCGEM   182
AnCyp4 ---TRDKPNQDVVIIQCGEM   183
AfCyp3 ---IRDKPSQDVIITQCGEM   181
SpCyp3 G--ANSRPKSNVAIVECGEM   173
DhCyp2 G--VLDKPYPDITITNCGEM   169
YlCyp2 ---VEEKPKRDVVVSECGEY   171
 ruler .......180.......190
```

N.B. The N-terminal sequence of UmCyp4 is truncated due to the absence of genomic DNA sequence 5′ of the current start of the protein sequence in the available genomic sequence database (GenBank ID: AACP01000148)

Cyclophilin - Group E

```
                    :    * :*.:     .:*::  : .*     * *:**.: .* *:****.******* ** .  ***:**:**:****    :* ** *    . :* ****  .* :.********* ************  ***** ******* .*:. ** *
ScCpr3 MFKRS--------IIQQSRLFSNSASRLGKKVFFDPAVNGTKIGRIEFELYDNVVPKTAENFRALCTGEKGWGYKGVPFHRIIPDFMIQGGDTDLTNGFGGKS--IYGSKFADENFVKKHDKAGLLSMANAGPNTNGSQFFITTVPCPWLDGKHVVFGEVTKGMDIVKAI   160
CgCyp2 MLSR---------ISMQKRFLSSTVAKLGKKVYFEPSVNGSRIGRIEFELYDDVVPKTAENFRALCTGEKGWGYKGVPFHRIIPQFMIQGGDTDLTNGFGGKS--IYGNKFADENFTKTHDKPGLLSMANAGPNTNGSQFFITTVPCPWLDGKHVVFGEVTKGMDIVKTI   159
EgCyp2 MLANSSRSL----LPSSRRLFSSSAASLGTKVFFDTSINGAKAGRIEFELYDDVVPKTAENFRALCTGEKGWGYKGVPFHRIIPDFMLQGGDTDLTNGFGGKS--IYGARFPDENFTKKHTRAGLLSMANAGRNTNGSQFFITTVPCPWLDGKHVVFGEVTKGMDVVKAI   164
KlCyp2 MFATTLKSARIANLAPVQRLFSSSAYLLRTKVFFDTSINGNKMDRIEFELYDDIVPKTAENFRALCTGEKGFGYKGVPFHRIIPNFMLQGGDTDLTNGYGGKS--IYGNKFADENFQEKHTKPGLLSMANAGPNTNGSQFFITTVPCPWLDGKHVVFGEVTDGLSTVKTI   168
YlCyp3 -------------MFRQIRHFSTSP-LVRNNVYFNVSANGNPLGKISFKLYKDQVPKTAENFRALCTGEKGFGYAGSSFHRVIPNFMLQGGDFTNHNGTGGKS--IYGQKFPDENFNVKHTRPGLLSMANAGPNTNGSQFFITTVDTPWLDGAHVVFGEVTDGMDVVKAI   154
CaCyp2 MFKTTLTK--PLTFK-SARFFSTTPRAWGTKVFFNTSINGEKQPPITFELYDDVTPITAENFRVLCTGEKGVGYKGSIFHRIIPQFMLQGGDFETGKGYGGYSPTHNNDKFNDENFIKKHDRPGLLSMANAGPNTNGSQFFITTVPCPWLDGKHVVFGEVVDGFDVVKKI   167
DhCyp3 MFK-------PVNFTRQTRLFSSSARNWGTKVFITPSIGGVKQSPIKFELYDEVVPKTTENFRALCTGEKGFGYANSIFHRVIPNFMLQGGDFETGKGYGGRS--IYGQKFSDENFQKKHEKAGLLSMANAGPNTNGSQFFITTVPCPWLDGAHVVFGEVIEGMDIVKKI   161
 ruler 1.......10........20........30........40........50........60........70........80........90........100.......110.......120.......130.......140.......150.......160.......170

       *  *: .* .  .:*:::.*:
ScCpr3 ESYGTASGKPRAEIVIEEAGEL---   182
CgCyp2 ESYGTMSGQPRAKIVVEESGEIKE-   183
EgCyp2 ESYGTGNGQPSAKVVIEDAGELKE-   188
KlCyp2 ESFGTVSGRPRAKIVIEECGELKD-   192
YlCyp3 EKLGSNSGRTSQKIVIDDCGQE---   176
CaCyp2 ESCGTSSGTPSASVVIEESGEAEK-   191
DhCyp3 EAQGSGSGAPQSSIVIEECGELKDN   186
 ruler .......180.......190.....
```

Cyclophilin - Group F

```
                        .    .    .         .                                          *** *.*:*. ****. **: **.     **  * ***:**:**:******. *****:****:*******   * :  :***************
NcCyp4_iso.1 MFGPRHFSVLKTTGSLVSSTFSSSLKP--TATFSCARAFSQTSSIMS-KVFFDLEWEGPVLGPNNKPTSEIKAQSGRINFTLYDDVVPKTARNFKELCTGQNGFGYKGSSFHRIIPEFMLQGGDFTRGNGTGGKSIYGEKFADENFAKKHVRPGLLSMANAGPNTNGSQF   167
GzCyp5_iso.1 -MRPSLFKPLLPRHTCFSAARTSSSLPQLSNTIRSVRFFSATSAVMGNKVFFDISWEGPVFQ-NGKPTSEVKKQTGRINFNLFDDVVPKTAENFRALCTGEKGFGYEGSSFHRIIPDFMLQGGDFTRGNGTGGKSIYGEKFADENFTLKHDKPGLLSMANAGPNTNGSQF   168
      AnCyp3 ----------MAPQVFFDVQYAP------LGTGAP--------------------------------------KTGRIIFNLFDDVVPKTAANFRELCKRPEKEGYKGSTFHRIIPNFMLQGGDFTRGNGTGGRSIYGDKFADENFKITHSRPGLLSMANAGPNTNGSQF   116
      AfCyp4 ----------MS-QVFFDVEYAP------VGTAETSARNCAQSGLDA----FGLQSILSILFTDGILLRAVTAKVGRIVFNLFDKDVPKTAKNFRELCKRPAGEGYRESTFHRIIPNFMIQGGDFTRGNGTGGRSIYGDKFADENFSRKHDKKGILSMANAGPNTNGSQF   149
      CnCyp5 -MQCPGDPPSPPHPLSFTSSFPK------TSKFTSTKHPPCPTFTLT-------------------LPSTVRQPAGRITFKLFDDVVPKTVQNFRELCTGQNGFGYAGSGFHRVIPQFMLQGGDFTNHNGTGGKSIYGNKFADENFKLRHDRPFLLSMANAGPNTNGSQF   144
       ruler 1.......10........20........30........40........50........60........70........80........90.......100.......110.......120.......130.......140.......150.......160.......170

             *:**. ****:*:*******:. :  .:*: :*: ** **: :    : .*  .* :
NcCyp4_iso.1 FVTTVPTSWLDGRHVVFGEVADDESMKVVKALEATGSSSGAIRYSKKPTIVDCGAL   223
GzCyp5_iso.1 FVTTVVTSWLNGRHVVFGEVADEESLNVVKALEATGSGSGAIKYTQKPKIEKSGEL   224
      AnCyp3 FITTVVTSWLDGKHVVFGEVADEESYSVVKEIESLGSQSGAPRSNVKPTIVNCGEL   172
      AfCyp4 FITTAVTSWLDGKHVVFGEVADEKSYSVVKEIEALGSSSGSVRSNTRPKIVNCGEL   205
      CnCyp5 FITTVVTSWLDGKHVVFGEVSSGQ--DLVRKIESYGSGSGATK--AKITIAASGTV   196
       ruler .......180.......190.......200.......210.......220......
```

Cyclophilin - Group G

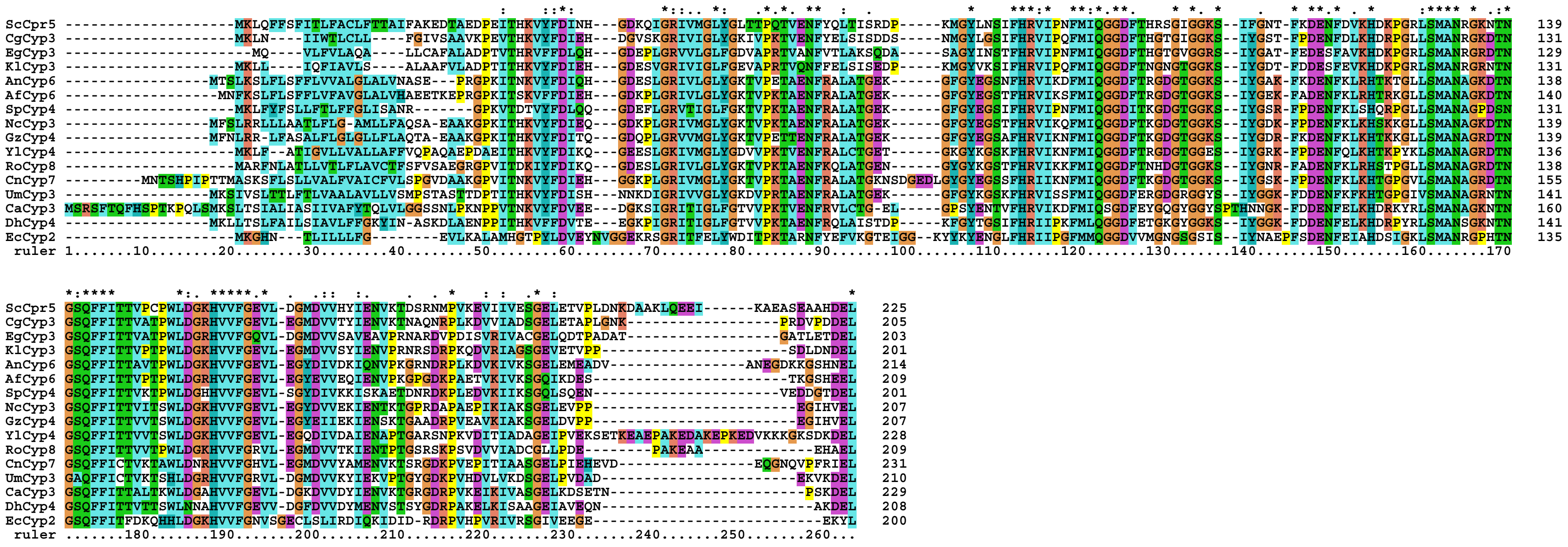

Cyclophilin - Group H

```
           .    :                       .  .    .*  :::  : :       .          :.   ::* . * : .**  :                        : : .:     *  *           :::.    : :*** :*.::  *::*
ScCpr4 MWLKSLLLCLYSLVLCQVHAAPSSGKQITSKDVDLQKKYEPSPPATHRGIITIEYFDPVSKSM-----KEADLTFELYGTVVPKTVNNFAMLAHGVKAVIEGKDPNDIHTYSYRKTKINKVYPNKYIQGGVVA--PDVGPFTVYGPK--FDDENFYLKHDRPERLAMAYF   161
CgCyp4 -MLANILLFLFTMLFSMQSTLADS------ADVDLRSMYAPDPPATNRISMTISYFPKGSDVK-----KEVDFSIDVYGTVCPKARDNFITLIRGIKAVIEGRG-SGVMTLRYPGTKFTNVLPNKYILGGEIL--PGISPYSIYGDK--WPEENFDLKFDRPGRIAMWNH   153
EgCyp4 -MTVGNGKRLVALSWVVLFGVMSYFGVISAAQAKSVKMYPPNPPISQRVQMLLRYD--GGEKQ-----EELEIGIELYGSVVPDTVKNFREIAKGVKAKIKGTD--QVLDITYKNTVFHRVVPEKYICGGKVL--DYR--FSIHGQT--FKDENFDIKHDRPGRLAMVND   154
KlCyp4 --MISKLFRLFCAVFVLASVTAGA------LVEDFKTLYEPNPPVTKRVLFGINYTDPSTNQP-----KAVDVGIELYGTVVPLTVNNFNELARGVKGQL-GDK---IIDISYKKTIFHRIIPGFMIQGGNVL--PHVGPFSIYGYA--FDDENFNLKHDRPGRLSMANS   149
CaCyp4 --MSSILLAVIFLVNLVLSSVIPQTDL--KLSVEEQSHLEGDPSVTHKVVFTI------KHGD----ELLGDLTLALFGETCPITVENFYQLAIRGEDGQGYKN-----------SKFHRIINNFVIQGGDY---DGRGGKSIYGDS--FNDENFILKHNKLGRLSMANA   140
DhCyp5 --MKG-LLGILFLCISVVSLVIDKHALMDKLTVQEINFLYRDPLITHKVHIEITKLAKRKNKDGVKPVVIGEIHAGLFGYTVPFTVNNFIQLANK-TNGYGYDDK----------TLFHRVIKDFMIQTGDYQFGEGYGGHSVYNNKGRFRDENFKLKHNKQGRMSMANG   156
 ruler 1.......10........20........30........40........50........60........70........80........90.......100.......110.......120.......130.......140.......150.......160.......170

       * ..:  :* * .        **:  :***: .*::  :. :      .         :       :             .    .   *  : . .    :
ScCpr4 GPDSNTSEFIITTKADGNEELDGKSVVFGQITSGLDQLMDAIQYTETDEYGKPQHELRFLYFVLEILKISNILDLHAAYTEKV-EKFRNGDVSVGSTLENIFRNDKAYTPLTTSTGTTAYD-----LNHPISRALMCLTVLGLCFIAYKG---MHEKPH-TVSLRHK---   318
CgCyp4 GQGKQESQFFISTNPKPDTELDGRYSIFGQVVSGLDVILNEVQHAEVSST----KDMILKYMVKEDLRLAKPDELHAKWLKRL-EEYNNGDKTKGVSIARYFKAEETAAIPKKMPANALYNGYVRHSGFPLFKVFFILVSLAVCFYVFKHRAEFLKRTN-IVSLKQWRSV   317
EgCyp4 GPDSNHSQFYIVTSLEPLEENDGKNVVFGQVYDGLEELLEKVRHLPLDEQGKPTSNLKIVGIHVDDLSIQNMDELVALYKQSL-VKYHAGDTSVGVPETAFLR--KGAGENGAVISSFEYS--------KQRVVVFGLVFALLYIAAKRGRSYLASKAN-VVSVRNG---   309
KlCyp4 GPNTNACQFFITTSETPLEHLDGKHVVFGQVISGLEDLMKYVQHVETDDKDKPVNDVSITYTYTEELRIADIEKMHEEYIKKV-EQFRNGDTSVGITLEEELK--EGAKNEVELENDYYYS------QHPYAKFVFAFLFIGALVTVYANRRSIIPRAKNIVSVRS----   306
CaCyp4 GPNTNGGQFFILNG-KPTPHLDGHHVVFGQLVDGFDT-LQKISTVET-KDSSPLKPVVISDVKTA-IDKSKVNQKEETGTSSS--SETEGITAVSTSVYSYLFIILLLG---VVFAAY----------------------------------------------------   249
DhCyp5 GPNTNGGQFFITTK-DECSWLDGKHVVFGQIINGFDT-LDLLNSARTDKNDRPKEEYVMSKITIETLDEDYLSTVDLSRVNDIGYDIMVGETSLSYTYISLFLLCAALG---MLYRRWHYR------------------RQAFSDMKDPDFYKMMPRRDQITTIKD----   299
 ruler .......180.......190.......200.......210.......220.......230.......240.......250.......260.......270.......280.......290.......300.......310.......320.......330.......340

ScCpr4 -----   318
CgCyp4 PETKA   322
EgCyp4 -----   309
KlCyp4 -----   306
CaCyp4 -----   249
DhCyp5 -----   299
 ruler .....
```

Cyclophilin - Group I

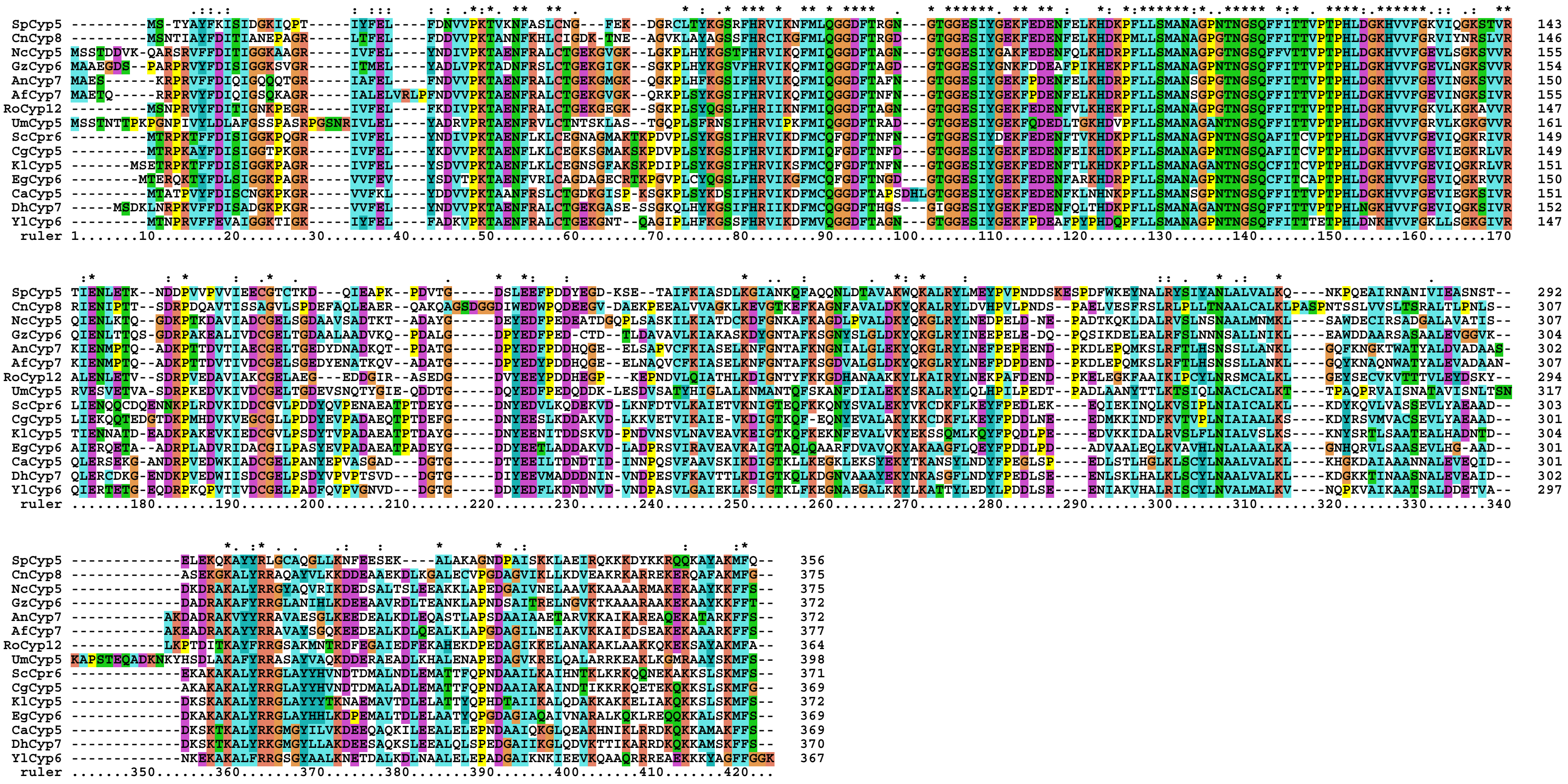

Cyclophilin - Group J

```
                 *:*:       **:* :*: : :* :  **  ** *             ::: . :.::::** :*.*.:                *. .*                    : .**      ..* * :** *  : * ****:*    .:: **:  **
CaCyp6 MQIEKVQPKAYFDITNGSKKLGRVVFELYDDLAPKATENFLNLCKG--ITLN---DKTYSYQDTIFDKVIKNFMIQGGSLN--------ANVSTIYGDQGIN-----------------KPIPGENLSDDLSHPFKLCMANNGDVNCNGSQFFITTSKQSQIAGKYSVFG   140
DhCyp8 MKVELLRPCSYIDISIGGSKTGRIVVELFDDLAPKTSENFIKLCNGSQITKE---GSPLTFKGNYFHRVIKNFVIQAGDIENGSSIKDYPNLNVGKGGISIYN---------------NEAFEDENLSEPLDDPFKLCLANDGTSNANKSQFFITTYPQPHLTGKHTVFG   152
ScCpr7 ---MIQDPLVYLDISIDKKPIGRIVCKLFREKAPKTTENFYKLCAGD-VKSPLKDQQYLSYKGNGFHRVVKNFMIQAGDIVFG-TQKDSSSSSVGKGGCSIYADKEEVK--TDDESFCYGNFEDENLG-EFVEPFTLGMANLGSPNTNNSQFFITTYAAPHLNGKHSIFG   162
EgCyp7 ---MSGVTRVYIDIEIGGCKSGRIVFELFQDKAPLSAQNFKQLCCGG-IEL---DGKHVSFRGNRFHRVIKNFMVQAGDIMYG-MEG-ADEENLGKGGCSIYSQQ-------DEEGPCRGAFADENTG-EFSEPFLLAMANMGAPDSNSSQFFITTTAAPHLNGKHSIFG   153
CgCyp6 -------MKVYLDIAIAGEQIGRIVCELYNDKAPKAVENFHRLCVGS-GSVG---GIHLTYKRSCFHRVIRNFMIQGGDLVFG-KEDNLDSDKVGLGGCSIYATEEEIKDDTK-ELQCHGLFEDENVG-EFDEPFLLAMANTGSPNTNSSQFFITTYPSPHLTGKHSIFG   156
KlCyp6 ------MHKVYLDIKIGDEPIGRIVCELFDEQAPKTCANFIHLCRGD-VNLN---GKVLSYKRNHFHRIIKNFMIQAGDLMFG-SDDKINKEEVGKGGCSIYATESELENSSRNEIQCFGNFPDENLG-EFTESFILAMANTGSQNTNSSQFFITTYPSPHLNGKHSQFG   158
YlCyp7 -----MSAFVYMDFAVGGEPVGRVVFELFDD-TPLTSANFRALCKGDKPTPE--GSVPLTFKDSNIHRIVRNFAIQGGDIVYG----------DGTGGTSIYG----------------DQFDDENFVHNHAEPFVLSMANAG-PNSNKSQFFVTLKGSPHLDGKHVAFG   135
 ruler 1.......10........20........30........40........50........60........70........80........90.......100.......110.......120.......130.......140.......150.......160.......170

       :*: **:::* :*   .   . *     : *  **       : : : .. . :  * *** * *:         :  :.   .  .:  **  .: : *     :           :  ** *.*.*     *: * .      :   *     *: :  : :  :
CaCyp6 HVIHGKSIIREIERVKTNKDDVP--ESNVVIDQCGVWTDDMPVPI-FNASYDTIGGDIYEEYPEDDNS------NFNQESTEEAFSVASKIKESGTLVFKKGDKEN-----------ARFKYIKALRYIMEFIPDPDQDPEWYKKFIDLKKKTYLNLSLCCLQLKDYHRC   290
DhCyp8 RVIHGKSVVREIERVNTDSQNIPTREDIVLIEDCGDWTENMEIPV-YNACYDQIGGDIYEEYPDDDE-------NIDKDSSELVYVASTKIKESGTILFKKGMKLE-----------AFLKYKKCLRYIMEYIPDEDQEPDWYKKYLDIKKKIYLNLSMVCLGLKNFIKA   303
ScCpr7 QVVHGKSVVRTIENCRVDSDGVP--ESDVRISDCGVWEKTMGVPL-YNASNDQIGGDVYEEYPDDDT-------HFGDDDFGKALEAANIIKESGTLLFKKKDYSN-----------AFFKYRKSLNYINEYMPEPDVDKERNIQFINLKMKIYLNLSLVLFNLERYDDA   311
EgCyp7 QVIHGKAVVREIEKEAVDSDGVP--EKAVRIAGCGEWTQDMPVPL-YNACNDPIGGDIYEEYPTDNT-------LFGGEEFGKAYAAAVLIKESGTQLLKRKDFQN-----------AFFKYRKSLNYVNEYIPDVDIDAEQFEKFNVLKQKLYLNMTLALFNMGSYDSA   302
CgCyp6 RVIHGKSVVRTIEYGKVDKEGTP--ENLVVIEDCGDWDETMSIPL-YNASNSTIGGDIYEEYPDDDT-------HFDSEDFAAAYNASNIIKDSGSALFKIKDYQN-----------SYYKYMKALKYVNTYIPEQDVDEKHCLLFIELKKKLYLNICLALFNLKDYDES   305
KlCyp6 KVIAGKSVVRTIEHMDVDENGVP--NDSIIIEDCGEWSDNMEIPL-YNACNDTIGNDFYEENPDDDT-------HFEGEDFAKAYEAAETIKESGSLLFKKRDFRN-----------ALFKYKKALKYTTAYTPEVDIDKFHNEKFTIQKHKLYLNICLMLFYLKEYHES   307
YlCyp7 KVVAGKSVLRQLEELDTAPGDVP--VLPVTITNCGELKRDAKLLLPFIGSQDGLTADKYEEHPDDNFNVGKEEGELNFDDPEQALRVTLEIKAYATEVYKMAGDAESEEERAMVLRVALQKYKKALRYVFELSPDPDSSKKAYDEFQKAKLALNQNMGLVALQLKKYDQV   303
 ruler .......180.......190.......200.......210.......220.......230.......240.......250.......260.......270.......280.......290.......300.......310.......320.......330.......340

              :: :       : :*   * . .    .  . .: :   .    ..   :   :   :       :. :   .***
CaCyp6 IDYCSYLLDMDDSVLTKQDKTKTLFRKGSANVGLKKYKLGLDDLKLANKLTPDDVSIQKSIESTEKLLQQQKQNEKATYSKFFG   374
DhCyp8 ADYSSYLLDMDN--LTAQEKAKGHYRKGCALFELRKYNDALTEFKDAKLLVPEDPAIGPQLDRCELLIELKKQNEKAKYSKFFS   385
ScCpr7 IMYATYLLEMDN--VPNRDQAKAYYRRGNSYLKKKRLDEALQDYIFCKEKNPDDEVIEQRIEYVNRLIEENKEKTRKNISKFFS   393
EgCyp7 ISYTNYVLEMDR--VPSMDLAKAYYRRGNCYLAKNLLEEALSNYKRCKEHNSD-EAVDMKIASVESKLAARLEKTKKSISKFFQ   383
CgCyp6 MKYGHYLLELDS--VDDKDKAKAYYRIGNSLFAKKRYDEALKNYRLCRDHNPDDNVIGQKIEKTENILEKEKERTKKNISKFFR   387
KlCyp6 IKYSTFLVDDDR--VGNKDKAKAYLRRGNCLYALNRFEAALTDYKNSAKTVPEDKNVEQKIDITEKKIASDLERRKKSIGKFFN   389
YlCyp7 INSTTSALEMEC--ATSRDRAKALSRRSSAYQGLKKQERAINDLEQAHELLPSDPQISKSLTALKQESEMKEKKQKAQYAKFFS   385
 ruler .......350.......360.......370.......380.......390.......400.......410.......420....
```

Cyclophilin - Group K

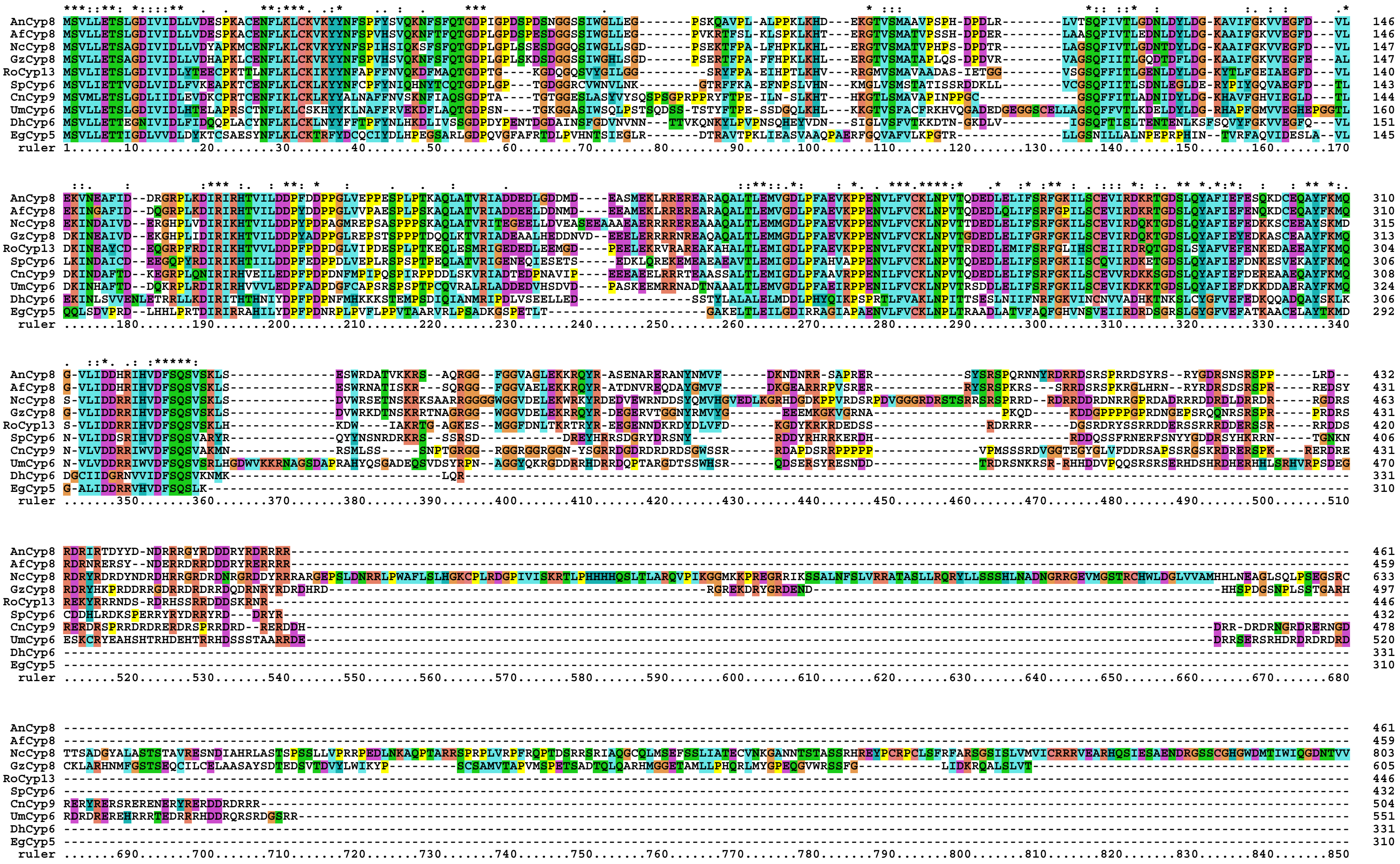

Cyclophilin - Group L

```
               **  ...   : *: * :   *: .: *     *:    .  : .  . .:                                                     * : *::: :  *              :     .     .      ::* : *.:::
CnCwc27 MSNLYATEPATNGKVIIDTTAGEIEVELWGKECPKAVRNFLALTMEGYYDGVIFHRVVPGFIIQSGDPTGTGMGGESFYGEP-------------------------FEDEIHGRLKFNRRGLLGMANNGSRNSNTSQ-FFITLDAAP-ELTNKHTMFGKIVGNTIFNVL 143
RoCwc27 MSNIYALEPHTNAKVILHTTSGDIEIELWGKEAPRATRNFIQLCLEGYYDNTIFHRIVPGFLVQGGDPTGTGQGGESVYEDG-------------------------FPDEFHSRLRFNRRGLVGVANTG-QNDNGSQ-FFITLDRAD-ELTKRHTLFGRVAGDTLFNVM 142
 SpCyp7 MATLTNLEPLATGTVILKTTRGDIQIELWCKEVPKACRNFIQLCLEGYYDGTIVHRVVPEFLIQGGDPTGTGMGGESIYGEP-------------------------FAVETHPRLRFIRRGLVGMACTE-NEGNNSQ-FFITLGPTP-EWNGKQTLFGRVVGDTIYNVV 142
NcCwc27 MSAIYNLEPQPTASAIIHTTQGEIAVELFAKQTPLTCRNFLQLALDGYYDNTIFHRLIPGFIVQGGDPSGTGHGGESIYDNGALSGDLDPWPMDQRRGKNAGPHGVNFKDEFHSRLKFNRRGLLGMANEGAPDTNSSQ-FFFTLGKAD-ELTGKNTMFGRVAGDTIYNLA 168
GzCWC27 MSAIYNLEPQPTASVIIHTTRGELSVELFAKQAPLTCRNFLQLALDGYYDNTIFHRLVPGFILQGGDPTGTGNGGESIYDGGAFSGDLDPWPMDQRMGKNAGPTGINFKDEFHSRLKFNRRGLLGSANESRPDTNSSQ-FFFTLDTAE-ELNGKNTMFGRIAGDTVYNLA 168
AnCwc27 MSAHYATEPAPTASATLHTTFGPLHIALFAKQTPLTCRNFLQHCMDNYYAGTIFHRVVPDFIIQGGDPTGTGSGGTSIYEYPEFEYDPDARDPNEK---------VVLRDEIHSRLRFNRRGLVGMAK-SEDGSYGSQ-FFITLANTERELNGQCTLFGRLEGDSLYNML 159
AfCwc27 MSAHYTTEPPPTASATLHTTFGPIHIALFANQTPLTCKNFLQHCLDNYYAGTIFHRIVPDFIVQGGDPTGTGSGGTSIYEYPEFEYDPEARDPNEK---------VVLRDELHSRLRFNRRGLVGMAK-SEDGTYGSQ-FFITLANAERELNGQCTLFGRVEGDSIYNVL 159
UmCwc27 MTSQYVTEPPSSGLVDLVTSKGTISIALFPTQAPLACRNFLTLALEGFYDNLVFHRLIPNFILQTGDPSATGTGGESIYGEP-------------------------FPIESHSRLKFNRRGLLGMAANQ-DRTNESQ-FFLTLDATP-ELTGKHTLMGKVEGKSIYTLV 142
DhCwc27 ---MSSLEPATTAKVALITTKGPIEIELWAKEVPNITRVFIQNCLDKKYIGTTFNKVIKDYLVQTSKIKEPATLK--------------------------------LKDEFHSRLKFNKRGLVGAVHDDKRNSNNVDSLFITLKPTP-EFNNNYVLFGKIMGDSIYNVV 134
ScCwc27 --MSSNIEPQTTAKCILYTTKGNIAIELWAKECPETCKRFLSMLSDGTFTNGEFKELKPTQWLMFN-ANSTGEYRT-------------------------------VAEEKNPRIRFNRDGLLGWDR-------RRNTWFITVLADSKHVLNDCNVFGKIVGKSIYIFR 129
EgCwc27 ----MSSEPSASGKCVLYTTKGELQIELWAKECPKTVRSFLQSIQDGKWDGIVLGKVAEDAVWAPAGLCAC-------------------------------------SAEINGRLRFNRRGLVGMAP-------GQDQPFFTLASRG-ELDGRAVVFGTLVGQSVYRLM 121
YlCwc27 ------MEPQTTAKVVLTTTKGPIEAELFAKEVPLACTRFLQLCKSGYYDSKPFYRVLPGELIQCGQQEAGNNTNSYP----------------------------KLKDEPHTRIKLKRGYLAMASEYTENNRRVPNSATTEFFIALKEIPFSGTVIGKITGDTIYNAQ 136
  ruler 1.......10........20........30........40........50........60........70........80........90.......100.......110.......120.......130.......140.......150.......160.......170

         :                      :   .:     :                                                               .
CnCwc27 NIGNLDTDK------EERPLIPPKIRRIHI-IENPFDDIVPRITAEEKKAQQKAKLEAKKDMEQRERR-AKAKKNTGLLSFGD--SEEIPEEEVTVK-KKSMTRQDLVD---PSEAEHTKSKTSKMTETFVNIPPSLKDLGKSRENEASEE-----KKAVDLKNIRAQHE 294
RoCwc27 KMTELEIDE------NERPLYPPRIKRTEI-VINPFDDILPRITEREK--RQQKELEKKKMLEEAKKKKAPKKKQLNLLSFGEEAAELEPPSHTVEK-TKMKSAYDFMETSAPTPTELIEELKKPVKEESTVISEKKDDAKQEEENKKAEEEERKRKKALEEERKRQKEE 302
 SpCyp7 RISELELDA------NQRPVFPPKIISTEV-IDNYFTDIKPRSTKEERE-KIANELAHQEKQKERNKLLKSGRRNKAVLSFGD-------EVDMPIV-KKPLRQKTPVSRSSDTTTELSKDLISSSSSIHSTYSSAQTGLTSAKVS--------------SDEYARQVDT 282
NcCwc27 KIGEAEVEEG-----GERPLYPIKITRIEI-LINPFDDMQKREKKPRPQQISRPTPAEKKQ--------KKRKGGKQLLSFGDE-VGDEDGEELPLP-KKPKFDTRIVADLDQDDSSKQSASKKSSAKRDAKPVANDVPKREEKPEPKPVKESRRSPSPQPVAQKKEQPP 322
GzCWC27 KMGEGEVDEA-----TERPTYPVKIERIEI-LINPFEDMKKRSR-VAAVAPSKTTTTKDKK--------KKRKGGKQLLSFGDD-EGD---DEMPVL-KKKKFDPRIVMEAPEEAPEQDEVRSKPTKAKKERASEKRVSIAQEEQD-NSDQTTPREP-PKEVRQK-PAPP 315
AnCwc27 KIAEAERIEG-----TERPVYPVKITSCEVGDLGPFVDKVKKRQVVATGPKTEEKPAAKKK--------KKAKPGKALLSFGGDDEED---EDMPIRPAKPKFNPMLVTDTK--LPEKESSTKG----EASQTRKRPRSPSPKRQPQPSAPPKNRPKTPEPTKQLPLPNP 307
AfCwc27 KIAEAERVER-----TERPVYPVKVVSCEVGELGPFAGKLKRRETIATAPAAEEKPAAKKK--------KKAKGGKTLLSFGGD-EGD---EDVPLRPSKPKFNPTLVVDAG--IPPANDAPKKTSPEAEQQTRKRPRSPSPRR--SLSAERKHRPKTPDPLTQLPLPDP 308
UmCwc27 ELVEGVELVD-----GDRPRYPIKLHEVRV-VENPFDDLQPRTTKKQRIAEERRKKHEMETR---------------------------VAEEQKRKRSKAKKNTGLLSFGAEEEAEDEVALKGPKSSHDLLKDDKHLSRQTIETSKSTKANTPAVSANISSKEKRFLAQ 279
DhCwc27 KINESELKS------EETPMYPAEITDIKI-LVQYFDDLVESKEHIAEPAKKKAKKAKKPR----------------------------VKLDYTLEDEEDTGFKMKSAHDLLSDSKLSNKLYANKKKGPSENNEKQKTIEKAQDS--------------SMETKKIVPE 255
ScCwc27 EILGGEIEASSRDNDVKRFMYPAVLKDVEI-TIPFFEDIFGSKRRLEDNEKKEQEP------------------------------------------AKKLVKSAKVKMVYEDEQEDDDGDVQKLKPRKRMILPAWIKDDSRSEG------------------------ 232
EgCwc27 EIAQGEVGDD-----GKTFVYPAEVRRAEV-TIPYFDGLSGQKRRAEPEQQAAPRP-------------------------------------------RKIATRVRLEYEESEDEASDPPLDVRIRAAHDILQDERLTDALHGDS------------------------ 218
YlCwc27 DIARGELTED------GYPMYVQTVQNVEIVLGGGLVQETQKAGADAGADAESRENKSGSR-------------------------------------DRPKKPKRKLQVNHDDDDDDEEPVFTKKSVSKLVEDKFKKDNTNVAKKP----------------------- 239
  ruler .......180.......190.......200.......210.......220.......230.......240.......250.......260.......270.......280.......290.......300.......310.......320.......330.......340

                                                                                                            :     .
CnCwc27 ----REK---AGGSAARQAEIKRMEEDLRRLKKRSGSVSDSESDSSSRARRKGPSYLEQELAKYASKRG---------RAAMKAGNKRGRRDEEEDVLTEMRKFSKRVMQAGDEP--EEEQAEEIEEGE-------------------AKEEGT-GIGAAMAEEE----- 421
RoCwc27 ----EKKKMQENTSESRASAIEKLKQDIRNLSKTSSNTSDELIPKKDKKR----SLVELEREKYASQK----------RKKMKKG-------DDTDVFNKLMSFQKKLSTAKED---EDAAAAKRDQPP-------------------CEIHGIPGCESCRDTTQ----- 420
 SpCyp7 ----LDTKLNSSSKSKVQEEISRLKSELRDLEKSGGSSNSKPVVVRPKKR----NILTEELEKYKKSK------------KVVLGKRKNLENDEESTLRALSSFQSKIRNAEDEDVMDSQYGSKIEDTP-------------------CSLHNVPGCFSCFDRLG----- 408
NcCwc27 KKHYSEHSSPEPEEPKKKSLLEKTNEEIAALKASMKRTIHSEPVKQEKKKSALEQLIPDTAIRGRKRRP---------GASSN------PTREEQEALDLLKSFKAKIETAP----PEKNAAQPAVNPD---VEDGEQDGQADEE-KVCDLHFIANCQSCKAWDK----- 464
GzCWC27 VKMEIEDESPEPEAPR-KTALERANEEMAALKASMRRTIHSEEPVKEKKKSALESMIPETSMRGRKRRP---------GAANT------SAADDAKALRMLKAFQSRLEKAP----PEK-ENEPAARET---TKDGEDAQAGDEEAELCDLHFIANCQSCTSWDK----- 456
AnCwc27 ---ESPERSP--SPPPKQSFLSRTNAEIENLKASMRRTAHAPAAET-KPKSALEAMIPQTAIRGRKRPPPGSVSAS--TSAPNGITGFSSNSAEAEALKMFNAFRAKLESSDSQPTAAAAKRLSVAKTD--AAEERKETPDEDEESQLCDLHFIANCQSCKSWDNDVEGG 467
AfCwc27 ---ESPARSPPQSPPARQSILSRTRAEIENLKASMRRTVATGPADTGRKKSALEAMIPETAIRGRKRPPPGTVNGAGRGSSTNGVTGFSS-AAEDETLRMFNAFKAKLESADAKSGPHGKTSISASDTTKYTSQAKSNTEPEDEEAQLCDLHFIANCQSCKSWDD--TGT 472
UmCwc27 -------SSSSTTPPAVTKQASKDGTSDAPHPTATLKSEHSGPSDQKASSSTGRDFLASQRAKYLSSSHP------------------STAKQDDSYSALLSFQSRLRTRP-------SSTTPIAKPLP-----------------------SVGVDEVEEEAG----- 388
DhCwc27 --------------RPDYKGSEEIENETKITSSENMNHETKQDKPNYKAKLDRNPNIDSDYDSDLDLSS-------------------------SESIDLFAFKQSNFQSS----------------------------------------------------------- 327
ScCwc27 --------------IKLDASLDQPQEALIREKTELHDNVDEATTKETESQENIKEEPMDKRER--------------------------------ETLAMLSKFQERIKNKNILK------------------------------------------------------- 301
EgCwc27 -------------PPPRPAEAPRGSDSAPCVASPDRRAEAAPPTDGPPSRCASLAGPAPASPGPVTDLAP------------------QLSAREQGTLSSLAEFRCKRGGKNPLLG------------------------------------------------------ 303
YlCwc27 -----------------KVEASAEQPEPAAVQATTTHHVQDAEDMTTEVDTVVSERLEKFRNMSRTKP-------------------------DTKPKSMLISQEDRIRRRLGLGPDEDIPSDASDPSS----------------------------------------- 326
  ruler .......350.......360.......370.......380.......390.......400.......410.......420.......430.......440.......450.......460.......470.......480.......490.......500.......510

CnCwc27 ---GGIEVDDDVGWLTHKLKFQVDDK--ELTRR--A---EDEYAVIDPR----------------------AKARDLLGKPDKKKLKGNPNR--RTVRNSGRNR------ 491
RoCwc27 ---DAEEDVSDAGWISHKLIFEKDLKGKDLMKRRET---VDDYVVIDPRDREAKAKQEEYERKKGIKSSSSSQRRDRMDHDDKRYRTEKRSRSDRHRRSRSRDRYDDRRR 524
 SpCyp7 ---EKNITETNSNWFAHRLVAENDPT----------------------------------------------RRTEELRIQRAEELKDVPSARPKKLLMKRDII------ 463
NcCwc27 --VEDNEDSGDEGWMSHKLSFAADKLGKDLSYRKKA---EEELVVIDPLAKARTLKEEKK----------ATRDAKTGGSSRAWDRGRRDRH------------------ 541
GzCWC27 --QEKDE-SDDEGWMSHALSFAADKLGKDLSNRRKA---EEELVVIDPREKARTLKDEKK----------AARDARQGNSGRAWDQARDAARNAKMAQAASLAGRGAK-- 548
AnCwc27 ANNGADDDVNDSGWLNHQLRFGKDTLGKDLNWKREH-QDVDTLMVIDPREKEKELADSSRP---------GTKRVKGRGLERDREREKKGRVGDLEWSKR--------- 558
AfCwc27 AEEAPDDDDRD--WLTHELRFGKDMLGKDLQWKREHPDDVDSLVVIDPREREKEFVGGKR-----------------RGLERDRERDRKRERVGDQEWDRRRREKP---- 559
UmCwc27 ---EYGASDDDDDWRSHRLDAGGQPLVAGQNAGKDT---LEDYEVLDPRDHTDRERNPKAE-----SSRDGKRGRDWVEHDRKYQNDRSRRHREHDKHPQQRRQRSII-- 485
DhCwc27 -------------------------------------------------------------------------------------------------------------- 327
ScCwc27 -------------------------------------------------------------------------------------------------------------- 301
EgCwc27 -------------------------------------------------------------------------------------------------------------- 303
YlCwc27 ------DEDDDFDIFKHKFICPEDDKAEDSLITLGA-------------------------------------------------------------------------- 356
  ruler .......520.......530.......540.......550.......560.......570.......580.......590.......600.......610.......620
```

Cyclophilin - Group M

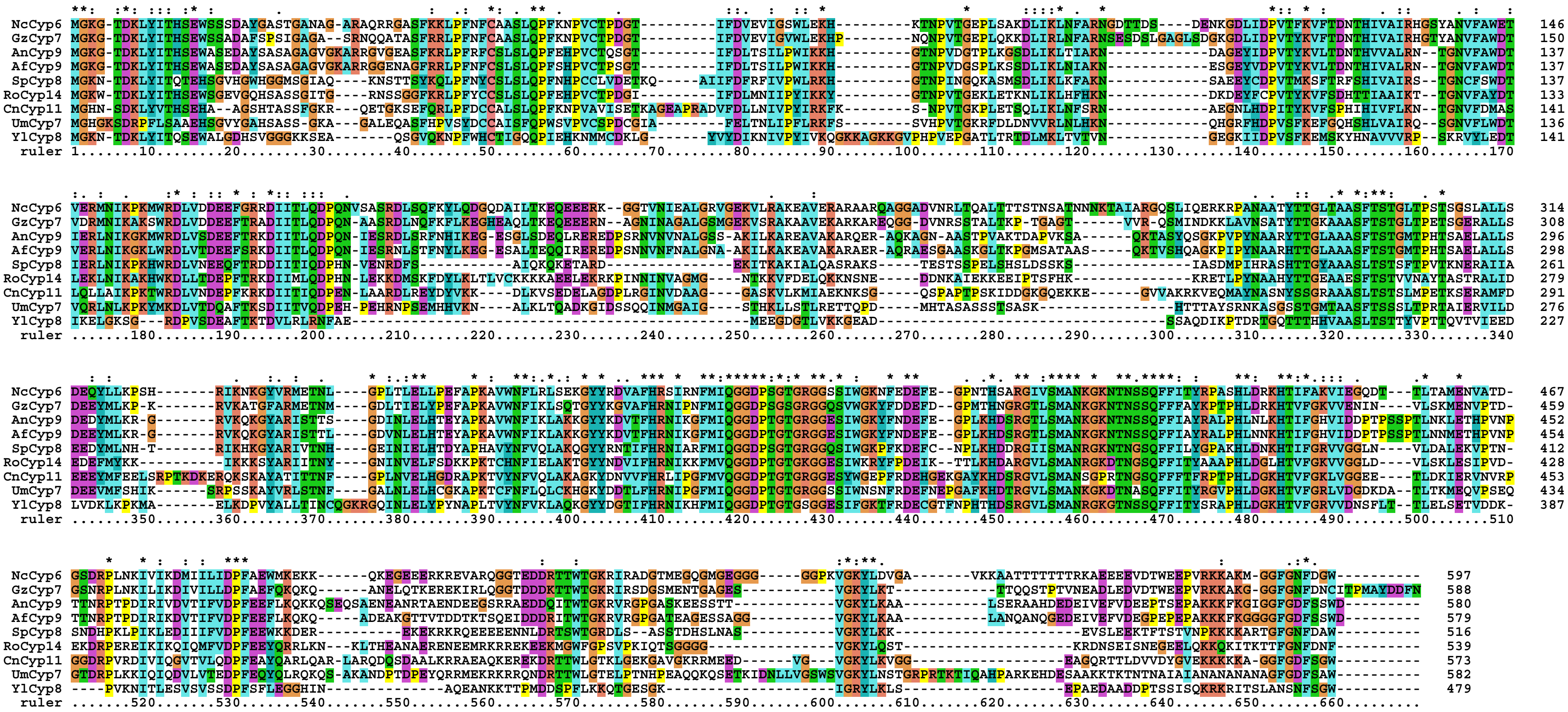

Cyclophilin - Group N

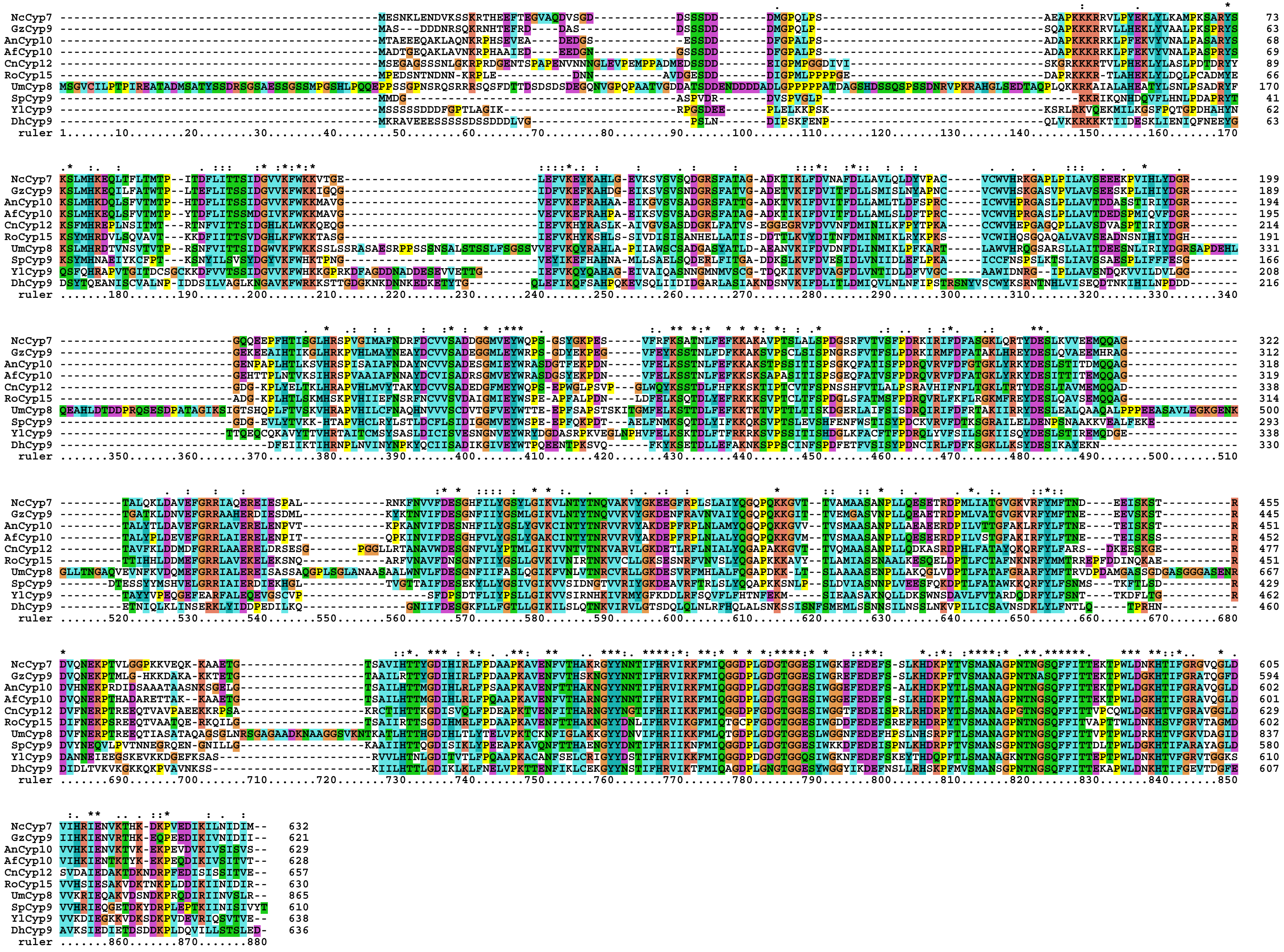

FKBP - Group A

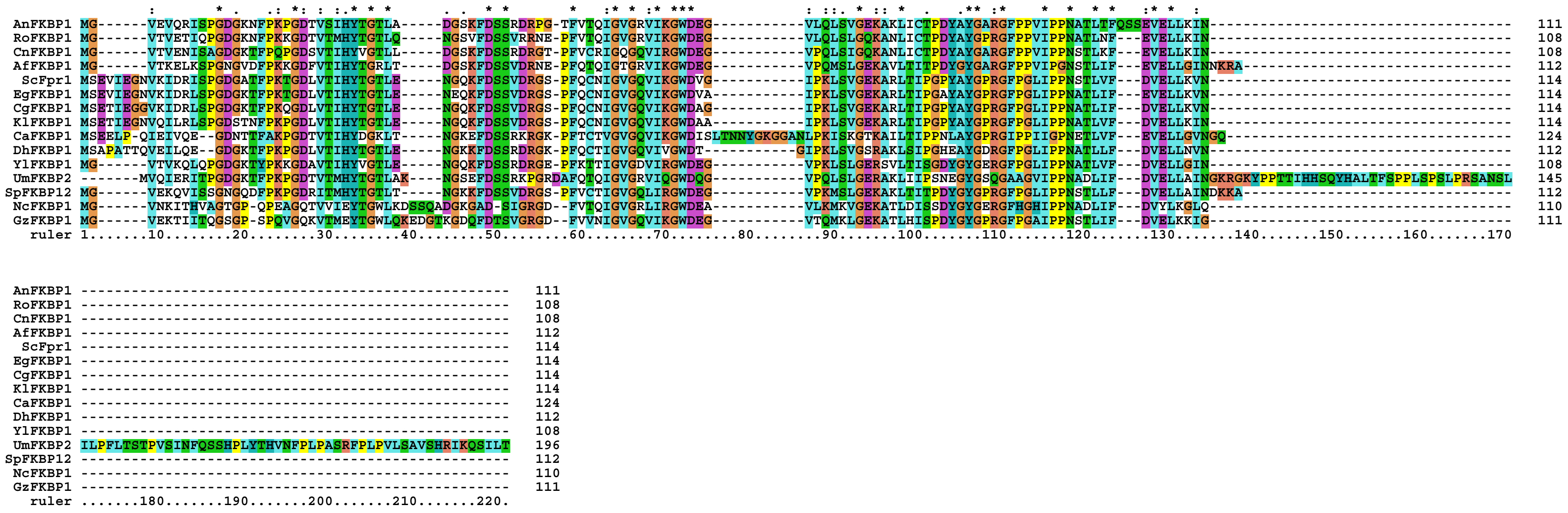
AnFKBP1
RoFKBP1
CnFKBP1
AfFKBP1
ScFpr1
EgFKBP1
CgFKBP1
KlFKBP1
CaFKBP1
DhFKBP1
YlFKBP1
UmFKBP2
SpFKBP12
NcFKBP1
GzFKBP1
ruler

FKBP - Group B

```
              *: *  ** ***:* *:.**.* ::*******::  : ::. *.:**:*: *****: **.*.** ***:**:**:********:*.: .**::********::.**: *        *
AfFKBP2 ----MGLEKQTLRMGNGKDHPQPGDPVELNYTGYLYDES--NPDHHKGKEFDSSKRRGPLKATIGAGDVIRGWDEGVRQMSLGEKAILTMSGEYAYGEKGFPGLIPPNASLVFEVELLKIKDHGLD   120
AnFKBP3 MNPPQGVTKTILRPGNGRDSPHTGDTVIIDYTGYLYDDTRGENEYFMGTQFDTSQGRGPLKTEIGVGKVILGWDKGVQQMTLGEKAILTISSDNGYGKRGFPGLIPPDSGLVLYVCSP----AG--   120
  ruler 1.......10........20........30........40........50........60........70........80........90.......100.......110.......120......
```

FKBP - Group C

```
               .                                   ::  .. : :** . :      *:::  *  *: . :* ..:: * :.*:  *  .* * * *..  .**  .    :*  : : .  :
KlFKBP2 -MKFTTGLSVLLFFVLQVFA--EKLTELVVGVTK--EPVDCKIKASKGDVVSVHYTGKLRDSGEIFDSSYNRGVPIQFKLGYSQVISGWDQGILGMCIGEGRTLHIPSELGYGSRGAGSVIPPDADLIFETELVDIQREAVDDEL-------------------------   140
YlFKBP2 MARIIVLIVAFMALIAGVFATEEKLAKLQIGILKKISPEECTQKARKGDTVSVHYTGKLED-GTVFDSSVERGQPIQFPLGTGRVIPGWDQGILGMCVGEKRKLTIPPHLAYGKQGAGRVIPPDSTLIFTTELVSIDNDGDRDEL-------------------------   144
AnFKBP4 -------MRVPIITTLLTLAL-TGLSQAASLGVETTHGVECNRKTTKGDTVKMHYRGTLAEDGSQFDASYDRGTPFKFKLGAGRVIKGWDEGLLDMCVGEKRTLTIPPEYGYGDRGIG-PIPGGATLIFQTELLEIEGVPKDEL--------------------------   135
AfFKBP3 -------MRILLLSALF-LSL-TTLVLSADLGIEKTHVVECNRKTTKGDTVHMHYRGTLAADGSEFDSSYGRNQPLKFKLGAGRVIKGWDEGLLDMCVGEKRTLTIPPEYGYGERGIG-PIPGGATLIFETELVQIEGVNNDEL--------------------------   134
NcFKBP2 -------MTIPQLDGLQ---------------IEVQQEGQGTRETRRGDNVDVHYKGVLTS-GKKFDASYDRGEPLNFTVGQGQVIKGWDEGLLGMKIGEKRKLTIAPHLAYGNRAVGGIIPANSTLIFETELVGIKGVQKGE---------------------------   120
UmFKBP1 -MKFCTGVVVCTLLASAVRAD-TRLSDKLQVGVKYRP-EVCDDKSQAGDLLAMHYTGTLA-DGKKFDSSLDRGQPFEFTLGIGQVIKGWDKGLRDMCVGEKRKLKIPPSEGYGSAGAGGVIPPNAHLIFEGKSAPCEPCVAQPHTY------------------------   142
CnFKBP2 -MYHPKALIIALLFSLSLILA-AKSAEQLQIGVKYVP-EECPVKSRKGDRLSMHYTGTLAKDGSKFDSSLDRNRPFEFTLGAGQVIKGWDQGLLDMCISEKRKLTIPSHLAYGERGHPPVIPPQSTLVFEVELLGIKNRHVDEL--------------------------   141
DhFKBP2 -------MLLKSLFLLFLTAI-AFASELQIGILTSVPDDKCKVKSKPGDLISVHYEGKLE-DGTVFDSSYSRGQPISFQLGIGQVIQGWDQGLTRMCIGEKRKLTIPSHLAYGDRGVG-PIPAKATLGMFAELVDIAGSSKHDEL-------------------------   135
 ScFpr2 -----MMFNIYLFVTFFSTILAGSLSDLEIGIIKRIPVEDCLIKAMPGDKVKVHYTGSLLESGTVFDSSYSRGSPIAFELGVGRVIKGWDQGVAGMCVGEKRKLQIPSSLAYGERGVPGVIPPSADLVFDVELVDVKSAA------------------------------   135
CgFKBP2 ---MLSQIWILFTFMVCVIASKSKSDGLKFEITKKIPISKCKLKALPGDMVSVHYTGSLAENGKVFDSSLRRNEPIQFKLGAGQVIAGWEQGITGMCLGEKRTLHIPPELAYGSRGAGGVIPPNAVLDFDVELVDIARN-------------------------------   136
RoFKBP2 -------MFSHVICKFLLTLSFITIIYAAKSESTINKPEKCGLKASSSSTVRIHYRSRVWGQEEYFESTYIREAPLEVKLGNGNLLKGIEDGIHGMCTGEIRRLLIPPNQAYGAIGIPNLVPPNTAIVVDVEMVNVNSPFSLWFWISGLILFSAFLLFGRKPIKGDTSNI   163
  ruler 1.......10........20........30........40........50........60........70........80........90.......100.......110.......120.......130.......140.......150.......160.......170

KlFKBP2 ----   140
YlFKBP2 ----   144
AnFKBP4 ----   135
AfFKBP3 ----   134
NcFKBP2 ----   120
UmFKBP1 ----   142
CnFKBP2 ----   141
DhFKBP2 ----   135
 ScFpr2 ----   135
CgFKBP2 ----   136
RoFKBP2 KKKE   167
  ruler ....
```

FKBP - Group D

```
        **: :: ** ***::***:* *** ****:** *:***:*** :::******:..*:***********:***:*.***********:********.**:**.:*****:**********:*****:*********                 :* **.*.::.*.  *::
NcFKBP3 MKSIFLSLSLLASATVGVLAAEELGIDVTVPVECDRKTRKGDKINVHYRGTLQSNGQQFDASYDRGTPFSFKLGGGQVIKGWDEGLVDMCIGEKRTLTVPPSYGYGQRSIGPIPAGSTLIFETELIGIDGVPKPESIVYKQAAEKAEEAASAVEEKVAEATDKAGGKIAD   170
GzFKBP2 MKAALF-LSALASTAVGVVA-EELKIDVTLPVICERKTQKGDGVHMHYRGTLKDSGKQFDASYDRGTPLSFKVGAGQVIKGWDEGLLDMCIGEKRVLTIPPEFGYGQRAIGPIPAGSTLVFETELVGIDGVPKPE----------------KIETKVVEGAESAAEAISE   152
  ruler 1.......10........20........30........40........50........60........70........80........90.......100.......110.......120.......130.......140.......150.......160.......170

        **    * *  ** :*. ***..: .**:*.**::*****. **:**
NcFKBP3 ATKKVEEKAEEASANVVEKVASVVSGAAEAVKTVVADTDDVQEHNEL   217
GzFKBP2 AT----EAAATASQKVAGKVAEAIVDAAKAAKTIIADTDDAPEHEEL   195
  ruler .......180.......190.......200.......210.......
```

FKBP - Group E

```
                .:.* :   .           *  .  . :    . :. . :                     ::                                                  .**:*   .  :  ::: :   : : *. .*:  :.* **::   :
 UmFKBP3 MSATPIGFFGLKLVPGKVHRMDVSRDFKITNVSYADQPKS-NTKTLVKIHYTHVPG----FEDDYDEEEQEKEPKSTD---------------------EEEEIEEK-------------VYTLCSLNGANKDHAVVDLQFCQEELIGFSITGDAPVDLVGNYVAPPDFF   131
 CnFKBP3 -MPLAMNLWSLTLLPGQQYPTYVRRDFQITNAALGEELRSKDGRSVVKVTHNPISQSMLESDDEWSDEDEDEEILSEEDDGEMEVEEVKQKKGKKAEKVEEEDSEEEDEDESDFEDELEETNVLCSLTAGKTEQASLNLTFVRGEVVVFEVTGDNVVHLMGNYIQQDEDS   169
 RoFKBP4 --MSVQGFWGLQLVPGKTYSQVVSAPFRITMASLAADAEA-GKRTSVSVLVD------------------EKE------------------------------------------------FVLCTLVPNKIEQQPLDITFVEGEEVTFSAKGQNNIHLTGNYVFQDDED   101
SpFKBP39 -MSLPIAVYSLSVKGKDVPAVEESTDASIHLTMASIDAGEKSNKPTTLLVKVRPRIPVEDEDDEELDEQMQELLEESQ-----------------------------------------REFVLCTLKPGSLYQQPLNLTITPGDEVFFSASGDATIHLSGNFLVDEEDE   128
   ruler 1.......10........20........30........40........50........60........70........80........90.......100.......110.......120.......130.......140.......150.......160.......170

         : : . .    .  ::                           : .. *.:   :          ..              :  .     .*  .  :   :   :  .   :   .       . *                           ::    .  . . .:
 UmFKBP3 DQDPSDSELYSDDEDDDED-----------------MYGLDS-DDFDDDDDDDMDEDPDRFEELVES-------SASKPKKAIEAAPLADSKKRAAEKPVKETAAKKLKADASAASAASTPTKAIETKGE---KQTKGAKDTKPKSETVEKKTVDKSTSKMTTTK-----   268
 CnFKBP3 D-DESDSDF--DGEDDYSE-----------------LYGSD--DDLELDSEE--EAAVAKITEIPDE-------PTPKTKKALPAA---DKKPVPEAKPAQKRKAEELESPAKEDAALSKAQKKKLAKKA---KVEGEKAEEKPAAAAVAEKPATKKEAKAPQKKT----   298
 RoFKBP4 D-EMGASMIDSDEEDNVEDFLKKLPPNASKEDINKALLGLEVDEEIESDEEVESDEEIESDEEIESEEEEEEPVPVSKKRPAEEVKEIASKKQKAEKKEQPKKEKSKKEEPKKEEPKKEQPKKEEPKKKEEPKKKEEPKKKEEPKKKEEPKKKEEPKKKEEPKK   270
SpFKBP39 EEEESDEDYDLSPTEEDLV------------------------ETVSGDEESEEESESEDNSASEED-----------ELDSAPAKKAQVKKKRTKDESEQEEAASPKKNNTKKQKVEGTPVKEKKVAFA-----EKLEQGPTGPAAKKEKQQASSNAPSSPKTRT----   254
   ruler .......180.......190.......200.......210.......220.......230.......240.......250.......260.......270.......280.......290.......300.......310.......320.......330.......340

             * .*::: : . *.*. .. *::: ***:*** *** **  ..**** * **:****:*** *: **  *.**::* *. :***::   *.** *:** *:***: ::
 UmFKBP3 ----LPSGLVIEEKSAGSGPPCKAGQKVGMRYVGKLTNGKVFDQCTSGKPFYFKLGKGEVIKGWDEGVKGMRVGAERRLTCPPKLAYGNQ-KIPGIPANSTLVFDVKLVEIK   375
 CnFKBP3 ----LPSGLIIEDIKIGDGPVAKTGKRLGMRYIGKLTNGKQFDANTSGKPFSFVLGKGEVIRGWDEGLAGMAVGGERRLTIPAALAYGNQ-KIPGIPKNSTLKFDVKLVSIN   405
 RoFKBP4 KITKLPNGLIIEDIKMGEGASCKNGQRVGMRYIGKLTNGKVFDKNVSGKPFSFLLGRGEVIKGWDLGIAGMKAGGERKLTIPAPLAYGKRGAPPDIPKNATLVFDVKLLSMK   382
SpFKBP39 ----LKGGVVVTDVKTGSGASATNGKKVEMRYIGKLENGKVFDKNTKGKPFAFILGRGEVIRGWDVGVAGMQEGGERKITIPAPMAYGNQ-SIPGIPKNSTLVFEVKLVRVH   361
   ruler .......350.......360.......370.......380.......390.......400.......410.......420.......430.......440.......450..
```

FKBP - Group F

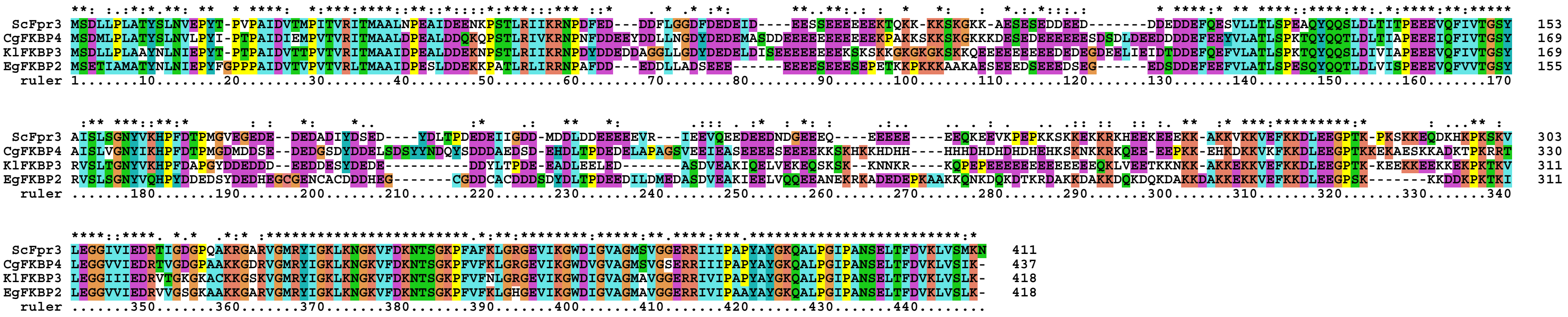
ScFpr3
CgFKBP4
KlFKBP3
EgFKBP2
ruler
153
169
169
155
303
330
311
311
411
437
418
418

FKBP - Group G

```
        ******** *:******: ***: :. ***:*************:**********:***  *  **  ::.  : ** **: .: :     .  . :..:.: *::.:.:*.**: : :**:****::****: : ***:************:*******************
ScFpr4  MSDMLPLATYSLNVEPYSPTPALNFKIPVTIRITMAAIDPEPFDDDKKPSTLRIIKRNPE-LTRGEYYNQDNNDGLEEDESESEQEADVPKRSVKSKKGKAVEQSESEDSEDENEIDDEFEECVLLTLSPKGQYQQALDITIAPEEDVQFVVTGSYTISLTGNYVKHPFD   169
CgFKBP3 MSDMLPLAMYALNVEPYTHTPAVLLDTPVTVRITMAAIDPEPFDEDKKPSTLRIIRRNPVYLDAGEVDEEKLIEELEGDEAAEDGDEASDDDKEEEDEDEDVDEDDEDDDEDD-DGEDEYEECVVVTLSPETRCQQAIDITIAPEEDVQFLVTGSYTISLTGNYVKHPFD   169
ruler   1.......10........20........30........40........50........60........70........80........90.......100.......110.......120.......130.......140.......150.......160.......170

        :. :   ****.*:* .*      **..: :.:* ..**.**:      :*.**:*:*:    * ::**:*::*::*: **:***       **:*: :*:   *  **....**:*  *..:.*:   * *:::****:*****  *:**:****::*****:******
ScFpr4  NSSD---SDEDEEDYYSD------EESSNGEEEEEEEEEDDEE------LSSGDDDLDD----LVDASDIESRLDELVKKDEKKKNNKKDSKRKHEEDEEESAKPAEKKQTTKKDKKAEKVKDSEESKPKPKTKLLEGGIIIEDRVTGKGPHAKKGTRVGMRYVGKLKNG   320
CgFKBP3 EPLEDLYSDEDSEEYSDDELDQEIEEDDELDHDEASSEESDEDQEFYDAISEGDEDIDEQLAKLEETSDVEAHLEDLIAKDNKKK-------RKQEQLDEQ---PETKKSKKTKDEKNTKATENEK---KNKAQVLEGGVIIEDRKIGEGPKAKKGSKVGMRYIGKLKNG   326
ruler   .......180.......190.......200.......210.......220.......230.......240.......250.......260.......270.......280.......290.......300.......310.......320.......330.......340

        *******.**** *** :***********:***:*****************:******************:*
ScFpr4  KVFDKNTKGKPFVFKLGQGEVIKGWDIGVAGMAVGGERRIVIPAPYAYGKQALPGIPANSELTFDVKLVSMK   392
CgFKBP3 KVFDKNTSGKPFYFKLHRGEVIKGWDIGVTGMAIGGERRIVIPAPYAYGKQTLPGIPANSELTFDVKLVSLK   398
ruler   .......350.......360.......370.......380.......390.......400.......410..
```

FKBP - Group H

```
        **.* **:*********:** **:*::*::**:***::****.*******:**:**:** *: **   ***  : * :* ****.******.:  *..**.* ** .:::***:*:* : *...*:**::****::******* *:***:*******************:
DhFKBP3 MSSLIPISTYNLALQPFNPVQAIDDEYPVTIRLTLAAVDPEAVDDKAEPSTLRLLKRSN-LFVDDEDLDDDLLDIEAEEADELDSEEEEEEVKP-KNKKKQNKKKVEEEEEDEDEEDLDIDGSSDEEDDEDVSEFVVCTLSPKVQFQQTIDLTITPDEEVYFVVTGSYPV   168
CaFKBP3 MSNLTPIATYNLALQPFQPVPAIEDDFPISIRITLASLDPEAADDKAEPSSLRILKKSNSLLSDDYFEDDDDDEEEDDEEDELDDEEEEEEAEEEKSSKKSNGKKSSKKDEDEEEDDEEEDDEDDDEDEDDVSEYIVCTLSPKHQYQQTLDLTITPDEEVYFVVTGSYPI   170
  ruler 1.......10........20........30........40........50........60........70........80........90.......100.......110.......120.......130.......140.......150.......160.......170

        ******:*****:*.*  *: *** :*:*:*:******* *   : : ** *::.:*   ****:*** :  *:   *.    .* .**********::******:*:************:..         **  *.*:       *** * *:: .*:   *******
DhFKBP3 HLTGNYVEHPADEDSE--DEYDEDSEDDYNLTPDEDEIIGGE--EYDLDDLEDASDE--NKIEELVEEAQGSKKRNAEE----PEAPTSKKSKKAKKEDKKSVQFTKDLEQGPTGSTLVEEKTEKKGKKEKAKKEEPKKEEPKKEQPKKEQPKKEQPKKEEASKKFPTKT   328
CaFKBP3 HLTGNYIEHPADQDEEDYDDEDEDYDDEYDLSPDEDEIIYGAPLDDEYDDEEESEEEGTPKIEEIVEEKEKVKESPKESKKRVAEESTSKKSKKAKKDEKKSVQFSKELEQGPTGSTLVEKDN---------KKATPTKD-------KKETPVKDDGDKK---KKFPTKT   321
  ruler .......180.......190.......200.......210.......220.......230.......240.......250.......260.......270.......280.......290.......300.......310.......320.......330.......340

        *****:***** *.* ***** ************************* * **************:**********:**************:*************:*
DhFKBP3 LLGGVVTEDRKTGKGQTAKSGNKVGIRYIGKLKNGKVFDKNTSGKPFVFGLGKGECIKGFDLGVAGMAVGGERRVVIPPKMGYGSQALPGLPANSELTFDIKLVSIK   435
CaFKBP3 LLGGVITEDRKIGSGATAKSGAKVGIRYIGKLKNGKVFDKNTSGKPFSFKLGKGECIKGFDLGVTGMAVGGERRVIIPPKMGYGSQALPGIPANSELTFDIKLVSLK   428
  ruler .......350.......360.......370.......380.......390.......400.......410.......420.......430.......440.......
```

FKBP - Group I

```
             *  .*:.*.**..  *:**. : .*                             ::::***:** : ** :.  . .  **:**:::: .     ::.*   *:* .   .  *    .*:* ******  *::* :: * :*:*    .::.    .*:::   .
AfFKBP4 MSGLLPVAVYALKVPAGGLLIPAVPDAAATVR-------------------------QFRVSMAAIDPDETPEFED--GQTR-PRATLKLIRPPADMDIDESD---DDYEE--DSEEDS---DDEEINGGPSDKEKARKLKEAAALKELEDEDEDDDSEGDDENFDLKAA  134
AnFKBP5 MSGVQPVAVYALRVPADGALVPAVPDAAAMVLE------------------------QFRVSMAAIDPDEAPEFDD--DSSRRPRATLRIIRAPPGLDEEDSD---DDYEDEDDSEDDSE--DDEEVNGGPSDKEKARKLKEAAYLKELEDAMSEDDESDEGEEFDLKAA  139
NcFKBP4 MAPLMPVAVFGLEVPPGEILIPAASEFPA----------------------------IIHITMAALDPTKAPEADGQGNIPALPRSTLKIIK-ATGHDHDDDDEEEDEYLQSLLGGGD----SDDEANGGPSDPSKSKKAKQEAAIKKLMAATQEES----DEEMEDAKP  133
GzFKBP3 -MSAVPGPVYGLEVPPGEILIPAAMEFPASSLSVAGASFATPLVLPAGDLNSNQKILQFRITMAAVDPTEEPEADGEGNIPTVPRSTLRLVKRALPGLEDEDDEIDDEYMKALLAGSDDEEDSDEEANGGPSDPAKAKKQRQAAAIKKLLESAQEES----DEEMEDAKP  165
  ruler 1.......10........20........30........40........50........60........70........80........90.......100.......110.......120.......130.......140.......150.......160.......170

         .*  ***. *:  .:: .* :**    :. :*:: *:****.**: ***::**: .**:*** * *:*::*****:::         *::::  :**:: * .  :      .      *  .*:** *   **: ** ::  ******** :.   *.        .*
AfFKBP4 ISKLVKGKAPAT--DDD-EDDESD----EGLELDEMVVCTLDPERHCQQPLDITVAEGERVFFKVTGTHTVYLTGNYVIPAEEGPSEYDEDEDDEDDEDDYDLSPDEDELVDMGDLLD--DEEEDELDGLA-HPRVTEIESD--EEEAPKLVESKGKNKRTADSDEEMAL  292
AnFKBP5 ISKLVKGKAPAT--DDDDEDAESD----EGLDLDEMVVCTLDPERNYQQPLDITVAEGERVFFKVTGTHTIYLTGNYVMPIDEPRDDYDEDDD--EDEEDYDLSPDEDE-LDMDELMMGEDDESDDLDGLE-NPRITEIDTD--EEEAPKLVDAKGKKKRGAD---EAAL  294
NcFKBP4 NGKKGKGKGKASESDEEESDEESDCCGDDDLQLEDYVVCTLDTERNYQQPINITIGEGEKVFFCVQGTHSVYLTGNFVVPE-------DDEEDSEDDEDESDDEDYDFP---LGGEDDDSDDMSDELDELDGTPRVKEITSEDEEEEAPKLVDTSKKGKKRPAEDDAEGL  293
GzFKBP3 NGK-AKGKAKATEESDDDEDEDSDDDSEEGADLENFVICTLDTERNYQQPLDITVNHGEKVFFVVTGSHTIYLTGNYIMDD-------DEDDEDSEDEDEYDLSPDELE---YGLEGDDSD-ASDDLDGLE-DPRVEEIDTD--EEEAPKLVAANK-GKNKRAAEEAAGL  319
  ruler .......180.......190.......200.......210.......220.......230.......240.......250.......260.......270.......280.......290.......300.......310.......320.......330.......340

        :   :*:.            ******* * ::.:      :                  *. ************** .   *            .. :  ** *:** :**:.:*.* :.  *: *.:******::** **:**** **:** ***:********: ***
AfFKBP4 DDMMAKDGKAKGADNGEPAPSKKQQKKLKKNNGEAAAVEQKKEAKVAKEGKEGKEGKEAKEAKKVQFAKNLEQGPTPSGQ-KP--------GETTTGTLGVKEVKGVKIDDKKLGKGPAAKAGNTVAMRYIGKLEDGKVFDANKKGKPFTFKLGKGEVIKGWDIGIAGMA  453
AnFKBP5 E---AKDDKAKSAANGE---SKKQQKKLKKNNGEASAVEAKP---------------EQKETKKVQFAKNLEQGPTPSKERKPDEKKPADKAEKTTGTLGVKEVKGVIIDDKKLGKGPAAASGNTVAMRYIGKLENGKVFDSNKKGKPFTFKLGKGEVIKGWDIGVAGMA  443
NcFKBP4 DAMISKDDKKL---------SKKQQKKQKVEEAKKEEPKKET-----------------KSDKKVQFAKNLEQGPTGPAKDKLE---------NKKPTSTVKVVQGVTIDDRKVGTGRAAKNGDRVGMRYIGKLQNGKVFDSNKKGAPFSFKLGKGEVIKGWDIGVAGMA  428
GzFKBP3 DELITKEDAKL---------SKKQQKKLKNNKGE-AVAAEEK-----------------KDAKKVQFAKNLEQGPTGSTTEKPKQ------AKDSKPATGVKVVQGVTVDDRTVGNGRTVKSGDTVGVRYIGKLQNGKQFDANKKGKPFSFKAGKGQVIKGWDIGVIGMA  456
  ruler .......350.......360.......370.......380.......390.......400.......410.......420.......430.......440.......450.......460.......470.......480.......490.......500.......510

        :*****:***.*****.:.:**** **.***********
AfFKBP4 VGGERRITIPPHLAYGKKALPGIPANSKLIFDVKLLEIK   492
AnFKBP5 VGGERRITIPSHLAYGKKGVPGIPGNSKLIFDVKLLEIK   482
NcFKBP4 VGGERRLTIPAHLAYGSRALPGIPPNSTLIFDVKLLEIK   467
GzFKBP3 IGGERRLTIPAHLAYGSRGLPGIPANSTLIFDVKLLEIK   495
  ruler .......520.......530.......540.........
```

Parvulin - Group A

```
        *. . . .   *... .*. * ..  .       ...      * * *:*********..****** *:.:*..*. *********** ******* :* *:  **:.*: ** *:* .**  *.** :*********:*
AnPar1 MG-KGNAKNSGGGDKKSKA-KAGDAKD-----DSKGK---MKGAQSVNVRHILCEKFSKKEEALEKIRNGAKFDEVAREYSEDKARQGGSLGWKSKGELELPFEEVAFSLEQSTTGNPKIGEAKTGYGYHIIMVEGRK   128
AfPar1 MAPKNNAK---GGDKKGKG-KDASEGD-----KGKGGGKGLKPATSINVRHILCEKFSKKEEALEKLRNGAKFDDVAREYSEDKARQGGSLGWKVRGSLNADFEKAAYELEPSTTANPKYVEVKTGFGYHIIMVEGRK   129
GzPar1 MG-KNDKK---GADKGGKA-KGGDKGKDAKDTKDSGSGGKAKGAQSINVRHILCEKHAKKEEALAKLNDGVKFDEVAREYSEDKARQGGSLGWKTKGSLDPKFEEVAFALETSTTNSPKFVEVKTGFGYHIIMVEGRK   133
NcPar1 MGKDKKAS---GSGSGSKGGKDAGNKD-----AGKDAGKASKGAQSINVRHILCEKHGKKEEALAKIRDGADFGAVAREYSEDKARTGGSLGWKQKGTLDPEFEKVAFALETSSTSSPKIGEVKTQFGYHIIMVEGKK   130
 ruler 1.......10........20........30........40........50........60........70........80........90........100.......110.......120.......130........
```

Parvulin - Group B

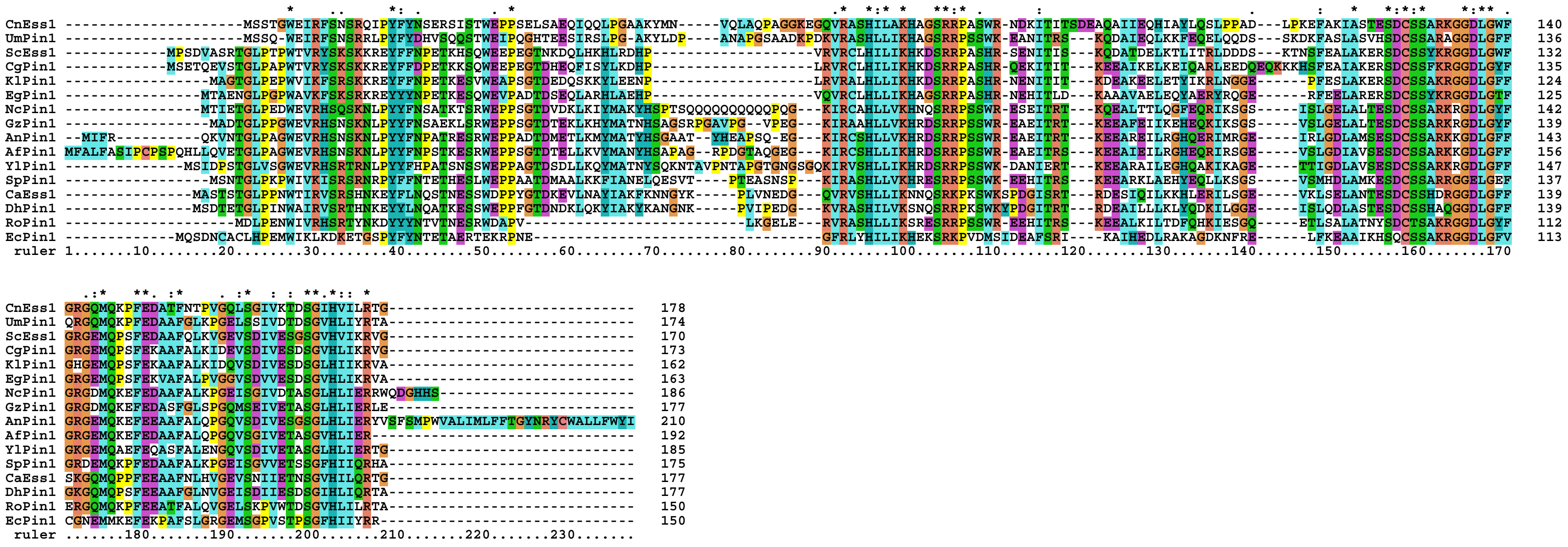

Supplement: Additional File 8 — Multiple sequence alignments of the different cyclophilin, FKBP and parvulin groups. Multiple sequence alignments of the different cyclophilin, FKBP and parvulin groups that were identified by BLAST analysis of their sequences. All alignments were constructed with ClustalX. [file 1471-2164-7-244-S8.pdf]
